# Expanding signaling-molecule wavefront model of cell polarization in the *Drosophila* wing primordium

**DOI:** 10.1371/journal.pcbi.1005610

**Published:** 2017-07-03

**Authors:** Juliana C. Wortman, Marcos Nahmad, Peng Cheng Zhang, Arthur D. Lander, Clare C. Yu

**Affiliations:** 1 Department of Physics and Astronomy, University of California, Irvine, Irvine, California, United States of America; 2 Center for Complex Biological Systems, University of California, Irvine, Irvine, California, United States of America; 3 Department of Developmental and Cell Biology, University of California, Irvine, Irvine, California, United States of America; 4 Department of Biomedical Engineering, University of California, Irvine, Irvine, California, United States of America; Lehigh University, UNITED STATES

## Abstract

In developing tissues, cell polarization and proliferation are regulated by morphogens and signaling pathways. Cells throughout the *Drosophila* wing primordium typically show subcellular localization of the unconventional myosin Dachs on the distal side of cells (nearest the center of the disc). Dachs localization depends on the spatial distribution of bonds between the protocadherins Fat (Ft) and Dachsous (Ds), which form heterodimers between adjacent cells; and the Golgi kinase Four-jointed (Fj), which affects the binding affinities of Ft and Ds. The Fj concentration forms a linear gradient while the Ds concentration is roughly uniform throughout most of the wing pouch with a steep transition region that propagates from the center to the edge of the pouch during the third larval instar. Although the Fj gradient is an important cue for polarization, it is unclear how the polarization is affected by cell division and the expanding Ds transition region, both of which can alter the distribution of Ft-Ds heterodimers around the cell periphery. We have developed a computational model to address these questions. In our model, the binding affinity of Ft and Ds depends on phosphorylation by Fj. We assume that the asymmetry of the Ft-Ds bond distribution around the cell periphery defines the polarization, with greater asymmetry promoting cell proliferation. Our model predicts that this asymmetry is greatest in the radially-expanding transition region that leaves polarized cells in its wake. These cells naturally retain their bond distribution asymmetry after division by rapidly replenishing Ft-Ds bonds at new cell-cell interfaces. Thus we predict that the distal localization of Dachs in cells throughout the pouch requires the movement of the Ds transition region and the simple presence, rather than any specific spatial pattern, of Fj.

## Author summary

In the tissues of a developing organism, specialized proteins can control cell growth and give cells a sense of direction, e.g., which way is the head or the tail, by having their concentration vary throughout the tissue. In cells of the developing fruit fly wing, a protein called Dachs localizes on the side of the cell closest to the center of the tissue, indicating a directionality. The localization of Dachs is determined by the spatial distribution, around the periphery of a cell, of intercellular bonds of the proteins Fat and Dachsous between adjacent cells. Here we asked how this cell directionality is affected when cells divide and when the concentration of Dachsous changes over time. We use a computational model to show that as the circular step-up region of the Dachsous concentration profile sweeps radially outward, like rings radiating outward from where a pebble was dropped in a pond, it leaves polarized cells in its wake. Our model also shows how cells can naturally recover their directionality after cell division.

## Introduction

**Fig 1:**
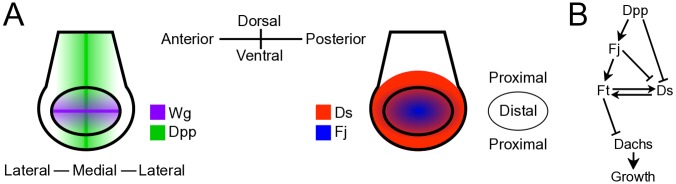
Visualization of components of the Ft pathway. (A) The approximate expression patterns of the morphogens Decapentaplegic (Dpp, green) and Wingless (Wg, purple), the protocadherin Dachsous (Ds, red), and the Golgi kinase Four-jointed (Fj, blue) in a late third-instar wing disc. The inner oval (distal region) corresponds to the wing pouch or wing primordium, while the tissue outside the oval (proximal region) will form the wing’s hinge in the adult. Dpp and Wg are produced along the anterior-posterior and dorsal-ventral boundaries respectively, and form graded concentration profiles along the axes orthogonal to the source boundaries [4, 13]. (B) The Fat (Ft) pathway. Increased Dpp activity is in general associated with more Fj and less Ds expression (although the relationship is not directly proportional and is likely affected by other factors such as Vg [5]). Ft and Ds form bonds with one another between adjacent cells. Phosphorylation by Fj makes Ft more likely and Ds less likely to form bonds. Dachs localizes to cell-cell interfaces where there is less bound Ft. Increased Dachs localization is associated with faster cell proliferation.

The growth and patterning of developing tissues are inextricably intertwined; epithelial cells typically become polarized along a body axis as they proliferate. This polarization is regarded as being dependent upon spatial variations in the concentration of morphogens. However, there are cases where cells become polarized even where there is little spatial variation in local signaling profiles. Furthermore, this polarization is maintained over time despite ongoing cell proliferation. A good example of this can be found in the *Drosophila* wing imaginal disc, a larval tissue that develops into the adult wing where cell polarization is manifested in the orientation of trichomes [1, 2]. In the wing disc, pattern formation is orchestrated by the morphogens Decapentaplegic (Dpp) and Wingless (Wg) whose concentration gradients are sketched in Fig 1A [3, 4]. Dpp and Wg signaling regulate both growth and polarization via the protocadherins Ft and Dachsous (Ds) that form heterodimers between adjacent cells (Fig 1B)[1]. In particular, Dpp and Wg signaling activate the transcription factor Vestigial (Vg), which in turn transcriptionally activates expression of the Golgi kinase Four-jointed (Fj) and represses Ds expression [5]. The asymmetric arrangement of Ft-Ds heterodimers around the periphery of each cell defines the subcellular localization of the unconventional myosin Dachs that determines the extent of the cell’s polarization. (In this paper, we use the term “Dachs localization” to refer to the direction within a cell, typically toward the distal part of the wing pouch where Dachs tends to reside. We use the term “Dachs polarization” primarily to describe the magnitude of this Dachs localization.) In particular, Dachs normally localizes to the side of the cell with the least amount of bound Ft [6–9]. On the other hand, the Golgi kinase Four-jointed (Fj) influences the magnitude of the polarization by affecting the binding affinities of Ft and Ds [10–12], i.e., phosphorylation by Fj makes Ft more likely and Ds less likely to bind.

Recent modeling studies have asked whether cell polarization patterns in the wing disc are established by the Fj gradient. A recent model by Jolly *et al*. [14] showed that when Fj has a spatially uniform slope or gradient as seen experimentally [12], i.e., a linearly sloping profile, the result is a pattern of Ft-Ds bond asymmetry similar to that seen experimentally. Moreover, if the model’s Fj profile is flattened in this model, Ft-Ds bonds become symmetrically distributed. Hale *et al*. [12] used a one-dimensional model to show that a linear gradient of Fj expression produces asymmetry in the cellular distribution of Ft-Ds bonds that is qualitatively consistent with the observed pattern, even when unphosphorylated Ft and phosphorylated Ds are capable of binding. However, these models did not incorporate dynamics, i.e., how cell polarization is affected by a moving Ds expression profile (see below) and how polarization is retained after cell division.

**Fig 2:**
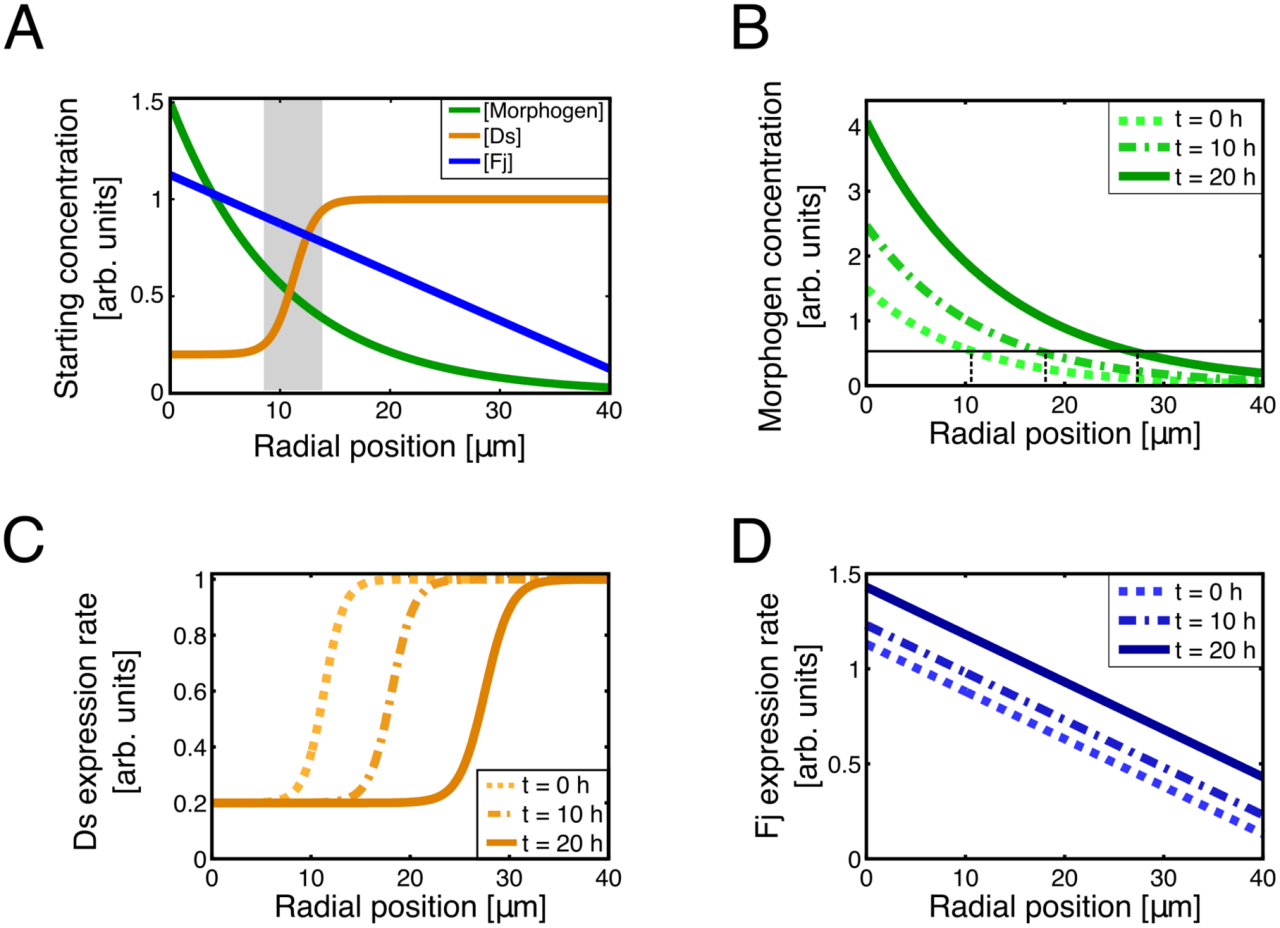
The expression patterns of morphogen, Fj, and Ds in the model change over time. (A) Initial concentrations of morphogen, Fj, and Ds as used in the model. The morphogen is most concentrated in the disc’s center and decays exponentially with radial distance (Eq 1). The Fj concentration is highest in the center of the disc and decreases linearly with radial distance. Ds is expressed in response to the morphogen concentration; it is most steeply graded in a narrow portion of the disc (shaded region). We refer to this as the “transition region.” The horizontal axis is the absolute radial position, measured in μm from the center of the disc. (A typical cell diameter in the model is 3–4 μm.) The initial radius of the disc is about 40 μm. The increasing amplitude of the morphogen profile causes the Ds expression front to move outward over time. (B) The morphogen concentration as a function of position at 0 (dotted light green), 10 (dashed medium green), and 20 hours (solid dark green) from the beginning of a typical run of the simulation. A radial position of 0 corresponds to the center of the disc. The amplitude (i.e., the concentration at the disc’s center) increases with time. Consequently, the position at which the morphogen concentration equals a given value, in this case 0.5 as indicated by the horizontal line, moves radially outward as indicated by the vertical dashed lines. In addition, the morphogen profile scales with the overall radius of the disc, so its decay length increases in time as the cells in the disc proliferate. Because the transition region corresponds to a morphogen concentration of 0.5 on this plot, this mechanism moves the Ds front outward as well. (C) The Ds expression rate profile as a function of position at 0 (dotted light orange), 10 (dashed medium orange), and 20 hours (solid dark orange) hours from the beginning of the simulation. Note that the Ds transition region, where the gradient is steepest, is a ring concentric about the center of the disc that starts with a radius of about 8 μm and moves outward over time. (D) The Fj expression rate profile as a function of position at 0 (dotted light blue), 10 (dashed medium blue), and 20 hours (solid dark blue) hours from the beginning of the simulation. The maximum concentration increases with time but the slope remains constant, so that the difference in Fj between adjacent cells does not vary substantially with time. Note that panels C and D show the expression rates of newly produced Fj and Ds, not necessarily the instantaneous concentration profiles. However, the initial concentration profiles of Fj and Ds have a similar shape.

Experimentally, Ds is expressed at low levels with a fairly flat spatial profile near the center of the wing disc and higher levels at the edge of the wing pouch, with a steep slope in a narrow transition region in between the edge and the center [5, 15–21], which expands radially outward as we sketch in Figs 1A and 2C. (By comparison, Ft expression is fairly uniform throughout the disc [22].) Since cells in the transition region have amounts of Ds that differ substantially from those of their neighbors, these differences can produce an asymmetric distribution of Ft-Ds bonds [7, 9, 11, 23–25]. In contrast, cells located far from the transition region have roughly the same concentrations of Ds as their neighbors. However, cells throughout the wing primordium, not only those near the graded region, still exhibit an asymmetric distribution of Ft pathway components [8, 26]. One could attribute this asymmetry to the linearly graded profile of Fj [12]. However, even in the absence of Fj, there is still a weak asymmetric Dachs polarization within cells [26]. Thus, Dachs polarization cannot simply be a readout of the current local concentrations of Ft, Ds, and Fj.

Mani *et al*. [27] proposed that even very slight cell-cell differences in Ds could lead to an asymmetric distribution of Ft-Ds bonds. By assuming that new bonds form preferentially with the same orientation as existing bonds, they showed that even a very shallow Ds profile can be amplified to produce the bond asymmetry and polarization seen experimentally. However, there is no specific experimental evidence that Ft-Ds binding exhibits cooperativity in this way. In addition this model focuses on the steady-state distribution of bonds, making the assumption that Ft-Ds kinetics occur on a much shorter time scale than other processes. The model thus does not directly address how polarization is retained after cell division.

In the wing disc, cells divide asynchronously approximately every 8 hours [28–30] so new cell-cell interfaces are constantly being created. Because the Ft pathway depends on the formation of Ft-Ds bonds at these interfaces, one might expect Ft pathway activity and the subsequent localization of Dachs to be disrupted by the creation of new interfaces with no Ft-Ds bonds. However, in a growing wing pouch, Dachs is typically localized at the distal side of each cell [26]. To the best of our knowledge, no evidence suggests that Dachs localization is disrupted by cell division. This result suggests that cells have some mechanism to recover or retain their polarization after dividing. For comparison, dividing epithelial cells in *Drosophila* have a reduced level of E-cadherin at the new cell-cell interface, requiring the formation of new adherens junctions (reviewed in [31]).

A model of cell polarization should take into account the growth of the wing pouch and the time evolution of the Fj and Ds expression profiles. In particular, the Fj expression domain grows as the pattern of Vg expands, pushing the Ds domain to the edge of the wing primordium [5]. Although the mechanism governing the movement of the wing pouch boundary is little understood, it involves increasing Dpp signaling [32] combined with the tendency of Dpp signaling to affect Fj and Ds expression [7].

Aegerter-Wilmsen *et al*. [33] have proposed an intriguing model in which differences in mechanical compression induce Vg and inhibit Ds activity, thus regulating cell growth. This model accounts for the experimentally observed patterns of cell proliferation and changes in Fj and Ds expression over time. However, this model does not explicitly include Ft-Ds binding at cell interfaces, instead giving each cell overall Fj and Ds concentrations. It then uses the extant Fj and Ds gradients in the tissue, rather than the arrangement of Ft-Ds bonds around a cell, to determine Dachs localization. This approach has the advantage of showing that Dachs localization can be polarized as long as either Fj or Ds is graded across the tissue. However, since it does not have explicit Ft-Ds bonds at interfaces, it cannot address the question of how cells respond to their history of Ds and Fj expression, or how cells retain their Ft-Ds bond arrangements after they divide and create a new interface.

The Ft pathway is associated not only with polarization but also with growth. In particular, greater asymmetry in the distribution of Ft-Ds bonds around a cell is associated with both stronger Dachs polarization and faster cell proliferation [7, 9, 34]. Finding the origin of distal Dachs localization throughout the wing pouch, then, would help to explain the rough spatial uniformity of cell proliferation that is observed experimentally [28, 35, 36]. However, cell proliferation can in turn affect the distribution of Ft-Ds bonds and Dachs localization, because it involves the creation of a new cell-cell interface between the two daughter cells.

In summary, there are two questions that have not been addressed by previous models. First, how is polarization (in the form of the Ft-Ds bond distribution and Dachs localization) affected by changes in the expression of Ds over time? Although the graded expression of Fj is one potential polarization cue, boundaries where Ds expression changes markedly also affect the polarization of nearby cells [8, 34] and the region of steeply graded Ds expression has been shown to move with respect to individual cells [5]. Second, how do cells retain or recover their polarization after cell division? To answer these two questions, a more realistic model of asymmetry in the Ft-Ds bond distribution is needed that incorporates Ft-Ds binding dynamics and cell proliferation.

Our model implements the changing expression profile of Ds and simulates the dynamics of Ft-Ds bond formation. The model gives rise to a consistently distal polarization direction throughout the pouch that arises from each cell’s history of Fj and Ds expression and naturally preserves this polarization after cell division.

This paper is organized as follows. We present a computational model in which cell proliferation depends on the rate of change of the morphogen concentration as well as on the asymmetry of the spatial distribution of Ft-Ds bonds around the periphery of a cell. The greater the asymmetry of the distribution is, the greater the polarization and growth rate of the cell. We show that cells near the edge of the wing pouch have an asymmetric distribution of Ft-Ds bonds around their periphery as a result of the steep transition in the concentration of Ds. This model reproduces the distalward polarization of cells in the wing pouch by taking into account the dynamic expansion of the wing pouch boundary. Ds-expressing cells in the periphery of the disc gradually become part of the Fj-expressing wing pouch, so that the wing pouch becomes an increasing fraction of the overall disc [5]. As the transition region sweeps outwards, it leaves in its wake polarized cells. Our model explains how dividing cells and their progeny retain this polarization after the transition region has swept over them.

## Models

### Model motivation: How is cell polarization affected by changing signals?

Cells throughout the wing primordium have asymmetrically distributed Ft-Ds bonds leading to cell polarization [8, 26]. How does this pattern of asymmetry arise? Certainly the linearly graded profile of Fj plays a role [7, 12, 14, 27]. However, the cell polarization pattern does not necessarily mirror the local Fj and Ds concentration profiles. For example, inducing a steep boundary of Ds expression can affect the polarization of cells some distance away [8]. The edge of the wing pouch, which is itself a steep boundary of Ds expression, sweeps over a large fraction of the disc during development [5]. This raises the question as to whether cells retain a memory of the history of the Ds expression levels over time. Specifically, we ask: what role do temporal changes in the expression of Ft pathway components play in Dachs polarization?

### How do cells retain their polarization after cell division?

Once polarization is established, how do cells maintain the polarization of their Ft-Ds bonds over multiple cell cycles? Cells in the disc divide several times during the larval phase, forming new cell-cell interfaces. Since polarization depends on the arrangement of Ft-Ds bonds around the periphery of a cell, how is the polarization of a cell retained after subsequent cell divisions?

In the next section, we describe the model that we have developed to address these questions.

### Model overview

Here we give an overview of our model; a more detailed description, including equations, is in Sections 2–3 of the Supplemental Text. We model the wing disc as a two-dimensional, roughly circular, cluster of cells (S1 Fig), because the wing pouch, our region of interest, consists of a single layer of columnar epithelial cells, bounded by the peripodial membrane. The cluster of cells is surrounded by unbounded free space. Each cell can grow and divide. Bulk forces prevent adjacent cells from overlapping or separating (S2 Fig) but have no other effect. The program keeps track of the location, size, and neighbors of each cell. Every cell also has certain amounts of morphogen, Ft, Ds, and Fj. Ft and Ds are distributed homogenously on the cell surface and form as many bonds as possible with Ds and Ft molecules in neighboring cells. Cell polarization and growth rate are functions of the asymmetry of Ft-Ds bonds (S3 Fig). The simulation progresses in discrete time steps with each time step corresponding to one minute of real time. (We obtain similar results when the length of the time step varies, which we discuss in Section 8.7 of the Supplemental Text.)

### An increasing morphogen profile determines the expression pattern of Ds

Experiments show that the amplitude of the Dpp concentration profile increases over time, due to some combination of increasing proliferation of Dpp-expressing cells and decreases in the Dpp degradation rate [32]. In addition, each cell in the disc experiences an increase in Dpp signaling over time even though, as the disc grows, individual cells move away from the Dpp source [32]. Experiments suggest, and previous models assume, that the Dpp profile scales with the size of the disc [4, 32, 33, 37]. This scaling may be the result of another spatially graded signaling factor, e.g., Magu (Pentagone), that affects the rate of Dpp binding or degradation [38, 39].

In our model we include a generic morphogen that can be thought of as a rough superposition of Dpp and Wg. (Although the Dpp and Wg profiles are not necessarily formed via the same mechanism, we simplify this aspect of the model for the sake of symmetry and to focus on the interaction between Ft, Ds, and Fj. Past models of wing disc growth and patterning [37, 40] have also made a similar simplification.) When the simulation starts, each cell has an initial amount of morphogen that depends on its distance from the center. The morphogen concentration is radially symmetric and decays exponentially with radial distance *r* from the center with a decay length that scales with the size of the disc (see Fig 2). As Fig 2B shows, the amplitude of the morphogen concentration increases exponentially with time as does the morphogen concentration in every cell. Thus the morphogen concentration [*M(r*,*t)*] at a given radial position *r* in μm and time step *t* in minutes is given by

$$\left[M\left(r,\;t\right)\right]=\;C_{0}\text{ exp }\left(\frac{t}{t_{0}}-\frac{Ar}{R}\right)$$ (1)

where *C*_*0*_ is the initial morphogen concentration in the center of the disc, *A* is a unitless constant relating the decay length of the morphogen profile to the size of the disc, *R* is the radius of the disc in microns at that time step, and *t*_*0*_ is the characteristic time over which the morphogen concentration increases by a factor of *e*, set to 1200 minutes. This value of t_0_ is based on the experimentally measured rate of increase of the Dpp concentration [32].

Fig 2 shows an example of the initial Fj, Ds, and morphogen expression patterns used in our model. Notice that the shape of the Fj and Ds profiles differs from the morphogen profile that drove them. The initial Ds concentration as well as the Ds expression rate (i.e., the number of proteins produced per time step) in a cell are Hill functions of the local morphogen concentration. This produces a sigmoidal profile with two plateaus connected by a region with a steep slope. The ring, i.e., the radial position, where the morphogen concentration is equal to the threshold value corresponds to the inflection point of the Ds profile. Over time, the morphogen concentration at all positions increases exponentially as described in (Eq 1), moving the location of the threshold concentration radially outward with respect to individual cells. The Fj profile, by contrast, is a linear function of radial position. Over time, its intercept increases, but its slope remains constant.

### Algorithm that determines the amount of Ft-Ds binding around a cell

Here is an outline of the steps followed in our simulation leading to the establishment and maintenance of Ft-Ds bonds around the periphery of a cell. (A full description can be found in Section 3 of the Supplemental Text.) The model begins at time step 0 with a population of 1000 cells, which is roughly the number of cells in the disc at the beginning of the third instar [28, 35] when the Vg-expressing wing pouch begins to differentiate from the rest of the disc [41]. (The periphery of the simulated tissue is not recruited into the pouch because the model is not intended to make specific predictions about the prospective hinge region.) Roughly 150 cells of the starting population of cells lie within the initial Ds front and represent the growing wing pouch. We assume that the Ft concentration at time step 0 is the same for all cells, and that the Ft expression rate is constant in time. The Ft and Ds in a cell are separated into phosphorylated and unphosphorylated pools based on the amount of Fj in the cell. (The more Fj that there is in a cell, the more Ft and Ds that will be phosphorylated in the cell.) Since phosphorylation by Fj makes Ft more likely and Ds less likely to form heterodimers [10, 11], the binding probability of any given Ft or Ds depends on its phosphorylation state. At each time step, a cell partitions its available Ft and Ds evenly among all its adjacent neighbors and forms Ft-Ds bonds at each interface, with probabilities weighted by the phosphorylation states of the individual Ft and Ds proteins involved. (Experimental data show that E-cadherin at mature adherens junctions is replaced on time scales on the order of minutes [42], so we assume that Ft-Ds binding takes place on similarly fast time scales.) Free, i.e., unbound, phosphorylated Ft and Ds can become unphosphorylated. However, unphosphorylated Ft and Ds at the membrane do not become phosphorylated, because we would not expect Fj in the Golgi to act upon Ft and Ds at the cell membrane. Ft-Ds bonds can dissolve, and only free Ft and Ds can degrade.

### Growth rate and polarization of individual cells

In our model the cellular growth rate depends on three factors. First, experiments find that Dpp is associated with cell proliferation [43–45] so, in our model, cells proliferated faster when the local morphogen concentration was higher. Second, *ft*^-^ *ds*^-^ double mutant discs are known to overgrow to a greater degree than either *ft*^-^ or *ds*^-^ single mutants [46]. Since a disc lacking either Ft or Ds would have no Ft-Ds bonds, the single and double mutants should differ from one another only in their number of free protocadherins, suggesting a link between the level of free Ft or Ds and slower proliferation. (However, *ft-* and *ds-* single mutants still overgrow compared to wild-type discs [46] despite having more free Ds and Ft, respectively. This is because mutants lack Ft-Ds bonds, causing the model to regard them as having a very high degree of bond asymmetry which produces overgrowth compared to wild-type. In other words, there are other factors that can outweigh the penalty of free Ft and Ds to the growth rate.) In our model, each cell’s growth rate was penalized based on the cell’s number of free Ft and Ds, whether phosphorylated or not. Third, experiments find that clones of cells that overexpress Fj or Ds exhibit increased cell proliferation near their boundaries, particularly when the surrounding wild-type cells express comparatively little Fj or Ds [7, 34]. Experiments also show that increased spatial uniformity in Fj or Ds expression leads to slower cell proliferation [7]. These results suggest that greater differences in Fj and Ds expression between adjacent cells are associated with faster cell proliferation, likely via the asymmetry of the Ft-Ds bond distribution around each cell. So in our model, cell growth is enhanced when there is more asymmetry in the spatial distribution of Ft-Ds bonds around the boundary of a cell. We measure this asymmetry on a scale from 0 to 1, using the “minimum fraction” method described in Section 5.2 in the Supplemental Text.

We describe the instantaneous growth rate of a given cell with index *i* in terms of the ratio of its radius r_n,i_ at one time step to its radius r_n-1,i_ at the previous time step using the following formula:

$$\frac{r_{n,\;i}}{r_{n-1,\;i}}=1\;+\;\;G_{0}\frac{\left(1+C_{Ft}x_{Ft,i}\right)\left(1+C_{Ds,i}x_{Ds,i}\right)\left(1+C_{M}x_{M,i}\right)}{1+U_{i}}$$ (2)

In (Eq 2), *G*_*0*_ is a constant scaling factor and *x*_*M*_ is a measure of the morphogen concentration. *x*_*Ft*,*i*_ and *x*_*Ds*,*i*_ reflect the asymmetry of the distribution of bound Ft and Ds protocadherins around a given cell with index *i*, respectively, such that the asymmetry of bound Ft and Ds varies between 0 (equal number of bonds on all neighbors) and 1 (at least one neighbor with no bonds). We then apply an adaptation mechanism in the form of integral feedback [47], as outlined in Section 5.1 of the Supplemental Text, to obtain the values of *x*_*M*,*i*_, *x*_*Ft*,*i*_, and *x*_*Ds*,*i*_. (Note that this feedback affects only the growth rate, and not the bond asymmetries or morphogen concentrations themselves. Thus it should affect the distribution and retention of Ft-Ds bonds only inasmuch as it affects the rate of cell division.) The constant coefficients *C*_*M*_, *C*_*Ft*_, and *C*_*Ds*_ represent the relative strengths of the effects on growth of the Ft-Ds bond distribution asymmetry and morphogen signaling. (Since *C*_*M*_ *x*_*M*,*i*_, *C*_*Ft*_ *x*_*Ft*,*i*_, and *C*_*Ds*_ *x*_*Ds*,*i*_ are all less than unity, the numerator could be represented by (1+*C*_*Ft*_ *x*_*Ft*,*i*_ + *C*_*Ds*_ *x*_*Ds*,*i*_ + *C*_*M*_ *x*_*M*,*i*_*)* with only a modest effect on the overall growth rate.) These effects are then divided by (1+*U*_*i*_) where *U*_*i*_ is proportional to the number of free (i.e.,unbound) Ft and Ds in cell *i*. This equation has the desired traits as can be seen by taking various limits. For example, if there were no morphogen, no asymmetry in the distribution of bound Ft and Ds and no unbound Ft or Ds in a cell, i.e., if *U*_*i*_ = *x*_*M*,*i*_ = *x*_*Ds*,*i*_ = *x*_*Ft*,*i*_ = 0, then the radius of the cell would grow at a (small) constant rate *G*_*0*_, i.e., (*r*_*n+1*,*i*_*/r*_*n*,*i*_) = 1 + *G*_*0*_. Notice that the “1” means that the cell cannot shrink. If there is morphogen present (*x*_*M*,i_ > 0) and/or asymmetry in the distribution of Ft-Ds bonds (*x*_*Ft*,*i*_ > 0; and/or *x*_*Ds*,*i*_ > 0), then the cell grows faster. The more free Ft and Ds there is, i.e, the larger *U* is, the slower the rate of growth.

Dachs is localized on the cell edge which has the least amount of Ft bound to the Ds of an adjacent cell. We represent Dachs polarization by a vector that points toward this edge. This vector has a magnitude equal to the cell’s bond asymmetry, a quantity between zero and unity. (This metric for bond asymmetry, its use in calculating *x*_*Ft*_, and the “grace period” for cell adjacency mentioned above, are described in detail in Section 5.2 and Eqns. S26 and S27 of the Supplemental Text.)

A cell’s probability of dividing in a given time step rises sharply as its radius approaches a given threshold. (Note that we do not account for the rapid increase in apical surface area often observed in mitotic cells in the wing disc [31]; the “cell size” we use corresponds more closely to a cell’s overall volume which tends to increase steadily over time.) More details, including mathematical expressions, values of these quantities, and division probabilities, are given in Section 2.2 of the Supplemental Text.

## Results

### Changes in the morphogen profile over time lead to an expanding Ds expression front

Zecca and Struhl [5] have noted that the expression patterns of Fj and Ds are not static. Rather, over time, the boundary of the wing pouch expands radially outward as Ds-expressing cells are recruited into the Vg-expressing population of cells. In our model, as the amplitude and the length scale of the morphogen profile grew with the disc (Eq 1), the steeply graded Ds expression front (transition region) moved outward, causing cells behind the front to express Ds at a lower rate (see Fig 2). Over the course of a run, the transition region swept over a large fraction of the disc.

### A moving front of Ds expression produced Dachs polarization throughout the wild-type wing pouch

**Fig 3:**
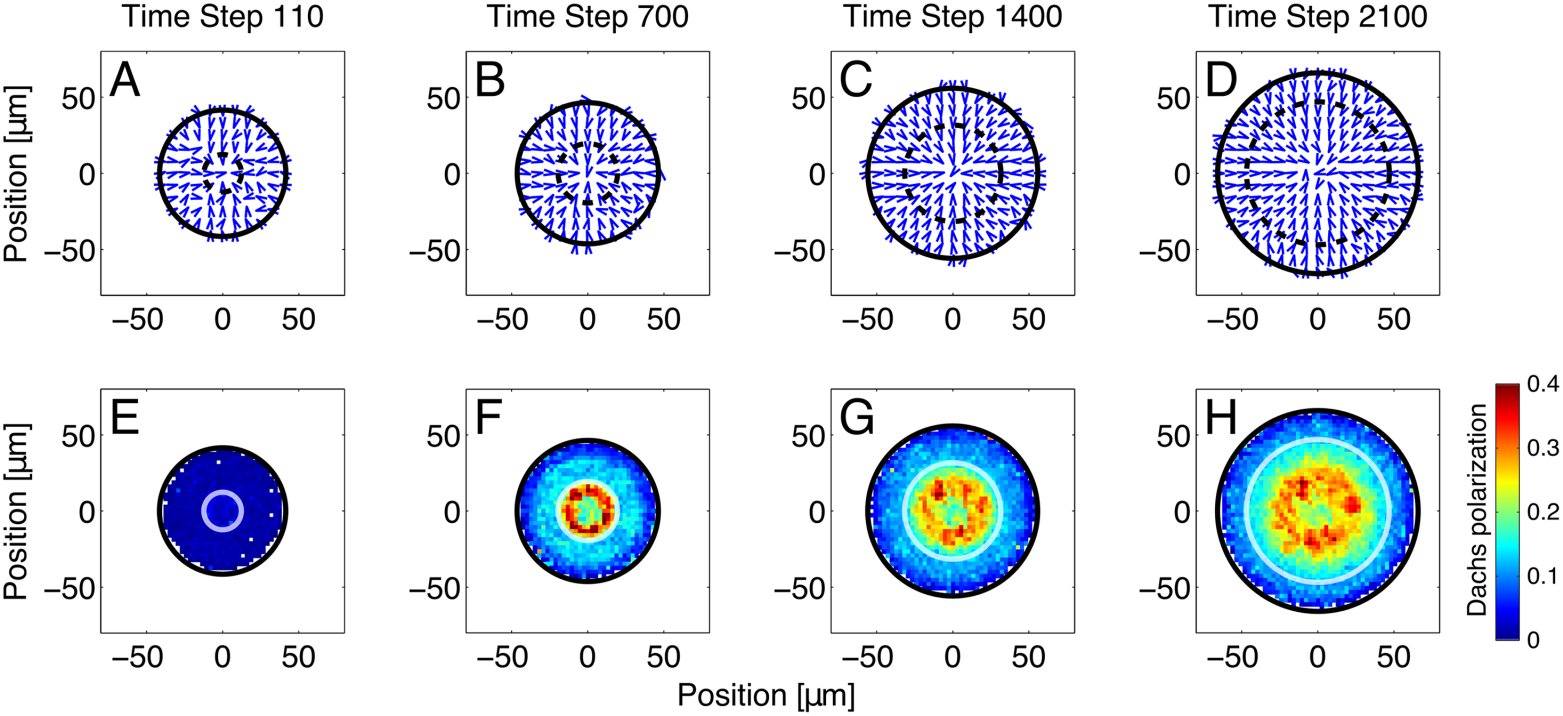
The Dachs polarization produced by our simulations of a moving front of Ds expression. Dachs polarization reflects Ft-Ds bond asymmetry. The direction (panels A-D) and magnitude (panels E-H) of Dachs polarization at 110 (A, E), 700 (B, F), 1400 (C, G) and 2100 (D, H) time steps (minutes) after the beginning of a single run are shown. (Because the simulation only begins to calculate Dachs localization after time step 100 and records data every 10 time steps, we display the state of the disc at time step 110.) The arrows in panels A-D represent spatial averages of cell polarization; they point to the side of the cell with the least amount of bound Ft. Panels E-H show the magnitude of Dachs polarization in the wing disc; blue represents weaker polarization, red stronger polarization. Each dot represents the Dachs polarization averaged over nearby cells. Dachs localization is a vector whose magnitude varies from zero, for an isotropic distribution of Dachs, to unity, if Dachs is all on one side. More details on simulated Dachs localization are given in Section 6 of the Supplemental Text. The solid black circle is the approximate edge of the entire disc, while the dotted black circle (A-D) and white circle (E-H) represent the Ds expression front. This front moved radially outward over time, encompassing an increasing fraction of the whole disc. Cells that had had this front pass over them (i.e., those inside the front) retained their elevated bond asymmetry for some time, leading to Dachs polarization throughout the wing primordium that was elevated compared to the hinge region. Note that cells within the Ds expression front tended to strongly favor having their polarization point inward; i.e., their bound Ft was predominantly on the outer (proximal) side, consistent with the localization of Dachs observed experimentally [7, 8, 26, 48]. Cells outside the front had weaker and less consistent Dachs localization. The Dachs localization in this outer region was primarily due to the Fj gradient. (S15 Fig in the Supporting Information shows similarly weak polarization when Ds is uniformly expressed).

In our model, Fj is graded and decreases linearly from the center to the edge of the disc, producing spatial variation in the Ft-Ds binding affinity from cell to cell. In addition, the Ds expression front expands outward through the disc. As a result, cells in the disc had an asymmetric distribution of Ft-Ds bonds and Dachs localized to the distal side of each cell, as observed experimentally [6, 7, 9]. (In our model, cells that had only recently become adjacent, e.g., via cell division, did not count as neighbors as far as Dachs localization and the minimum fraction were concerned. Otherwise, newly adjacent cells would have had very little bound Ft, and Dachs would simply have tended to localize toward a cell’s newest neighbor.) The general algorithm to determine cell adjacency is described in Section 7 of the Supplemental Text, while the special case of cells that recently became adjacent is discussed in Section 5.2 of the Supplemental Text.) Fig 3 shows the direction (panels A-D) and magnitude (panels E-H) of Dachs polarization at four different times during a simulation run. Near the start of a run (Fig 3, panels A-B and E-F), only cells close to the front had strongly polarized Dachs, while cells outside the front had Dachs vectors that are randomly oriented. As the simulation continued and the front passed over more cells, cells that had been recruited into the wing pouch due to movement of the Ds front also began to display Dachs polarization (Fig 3, panels C and G). However, changing Ds expression rates took some time to affect Ds concentrations and, in turn, Ft-Ds bond populations, so the region of strongest Dachs polarization lagged somewhat behind the location of the Ds expression front. After 35 hours, nearly all of the cells had Dachs localized to their distal, or center-facing, side (Fig 3, panels D and H) in agreement with experiments [7, 8, 26, 48]. Note that the region nearest the center of the disc, whose cells have never had the front pass over them, remained weakly polarized for the duration of the simulation.

**Fig 4:**
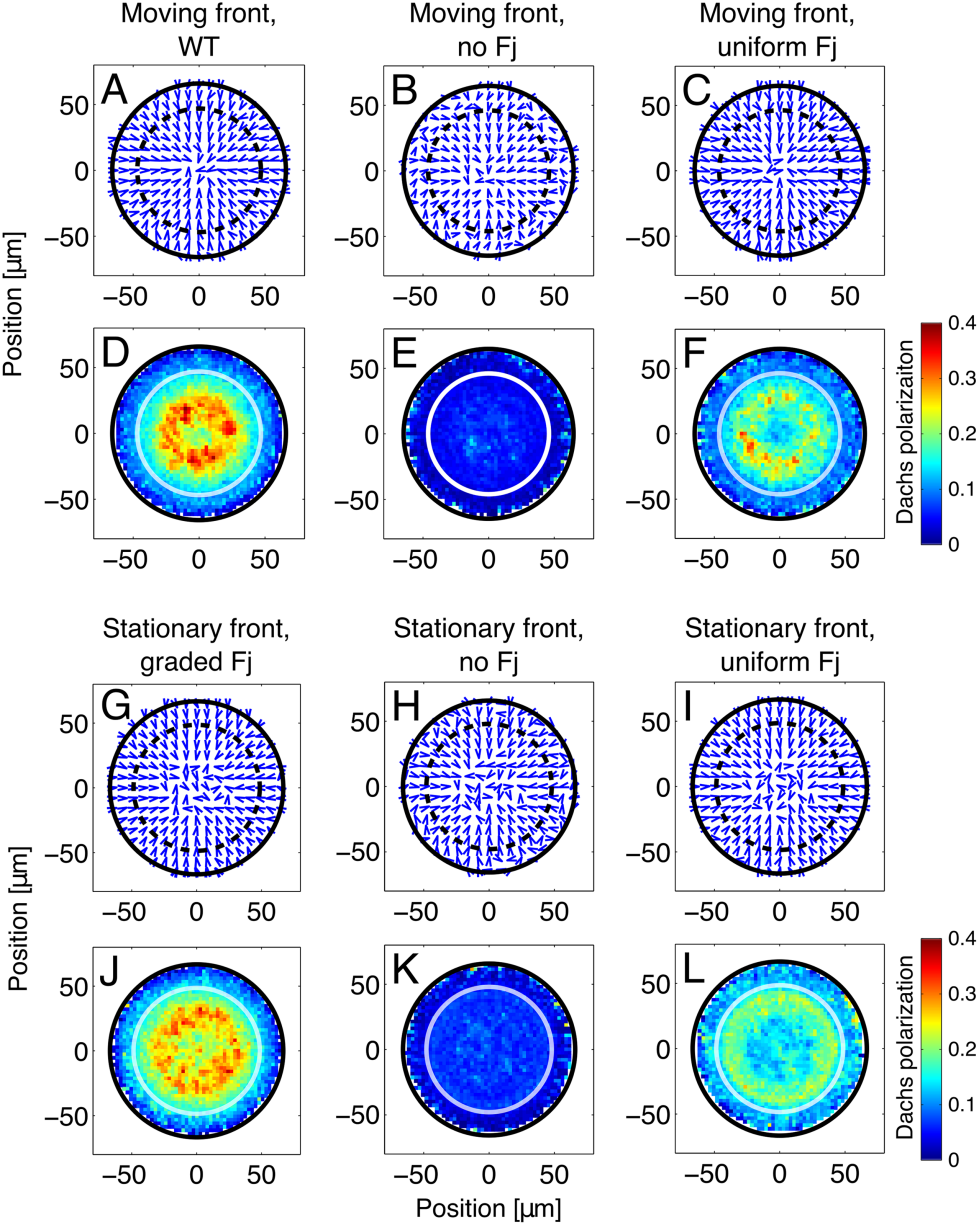
Dachs localization under different conditions of Fj and Ds expression. We show the direction (panels A-C, G-I) and magnitude (panels D-F, J-L) of Dachs localization at minute 2100 with a moving (A-F) and stationary (G-L) Ds front, and with wild-type (first column), absent (second column), and uniform (third column) Fj overexpression by roughly a factor of 10 times more than wild-type. Note that Dachs near the front is distally localized in all cases, but this polarization is stronger when Fj is present and stronger still when it is graded. Furthermore, when the Ds expression front is moving, cells in the wake of the front exhibit stronger Dachs polarization than when the front is stationary and has not passed over these cells. When the Ds front is stationary, cells near the center of the disc have randomly oriented Dachs, regardless of the Fj profile (panels G-I). However, the overall degree of polarization is dependent on the Fj profile (panels J-L).

To differentiate the effects of the Fj gradient and the moving Ds front on Dachs localization, we also ran simulations in which either the Fj or the Ds expression pattern was spatially uniform. In the absence of Fj but with a moving Ds front, Dachs was still distally localized but with a drastically reduced average magnitude (Fig 4, panels B and E and S17 Fig in the Supporting Information), in agreement with experimental results [26]. Similarly, when Fj was uniformly expressed at a high level (Fig 4, panels C and F and S14 Fig in the Supporting Information), Dachs was distally localized in the wing pouch but at a lower magnitude than in wild-type. Thus, we predict that Dachs polarization, like Ft-Ds bond asymmetry, originates primarily from the Ds front and is amplified by the Fj gradient.

### Maintaining a stationary front of Ds expression leads to increased Dachs polarization only near the front

Next, we considered a disc whose Ds expression front was held stationary in terms of its relative position between the disc’s center and edge, rather than being determined by a critical value of the morphogen concentration. In this case, individual cells primarily expressed either high or low levels of Ds for the entirety of the run without the front passing over them. Even though the front was stationary, the morphogen and Fj profiles were the same as with the moving front; i.e., the amplitude of the morphogen profile still increased with time and Fj had a linearly sloping profile that decreased with increasing radial distance from the center. (The results were essentially unchanged if the morphogen amplitude was constant in time.)

**Fig 5:**
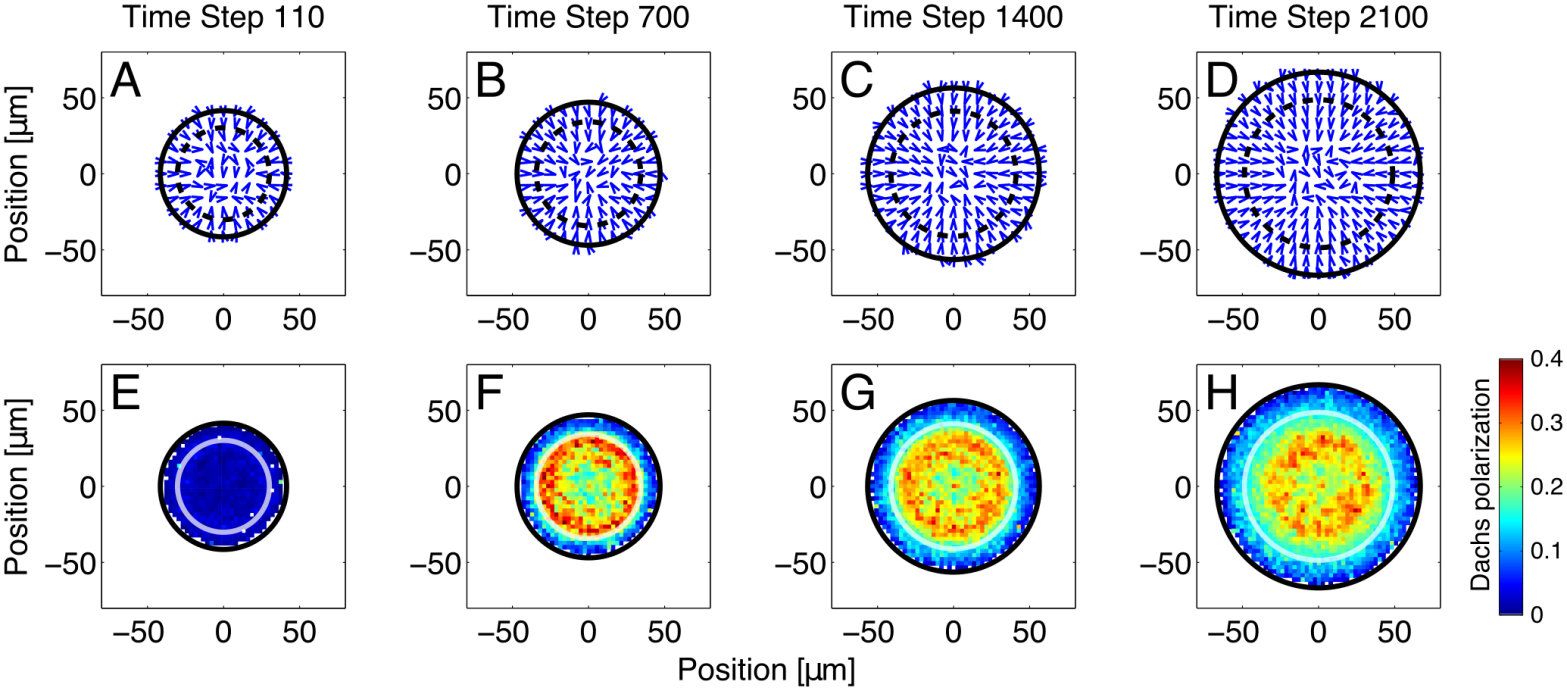
The annulus of Dachs polarization produced by a stationary Ds expression front. Again, the direction (panels A-D) and magnitude (panels E-H) of Dachs localization in the disc in a single run are pictured. Dachs localization was a vector whose magnitude varies from zero, for an isotropic distribution of Dachs, to unity, if Dachs was all on one side. More details on simulated Dachs localization are given in Section 6 of the Supplemental Text. The arrows in panels A-D represent spatial averages of the direction of Dachs localization; panels E-H show the magnitude of Dachs polarization. Blue represents less asymmetry, red represents more. Images are taken at 110 (A and E), 700 (B and F), 1400 (C and G), and 2100 (D and H) minutes into the third instar. Because the same set of cells was near the Ds expression front (black dashed circle in panels A-D, white circle in panels E-H) throughout the run of the simulation, this set of cells experienced elevated Dachs localization (red ring in panels E-H). Dachs localization near the front pointed predominantly inward due to the steep local slope of Ds, and was weaker and less directed near the disc’s center and edge, far from the front (green/blue markers in panels E-H). The Dachs polarization in these regions away from the front was primarily due to the Fj gradient. (S15 Fig in the Supporting Information shows similarly weak polarization when Ds is uniformly expressed.)

When the front did not sweep over a large fraction of the disc, only cells close to the front where the local gradient of Ds remained relatively steep had asymmetrically distributed Ft-Ds bonds and asymmetrically localized Dachs. Furthermore, cells near the disc’s center and edge consistently experienced spatially uniform Ds concentrations, leading to more symmetric bond distribution and less polarized Dachs. Fig 5 shows the direction (panels A-D) and magnitude (panels E-H) of Dachs localization in a disc with a stationary front. (Note that individual cells could still move away from the front as they and their neighbors proliferated, but the spread of polarization was less pronounced when the front’s location was fixed.) In contrast to the polarization pattern observed with the moving front in Fig 3, the case of the stationary front produces a polarization pattern that is strongly correlated to the spatial expression pattern of Ds.

To differentiate the effects of Ds and Fj on Dachs localization, we also examined polarization patterns with a stationary front with uniformly expressed Fj and in the absence of Fj (Fig 4, panels H, I, K, and L and S20 and S21 Figs in the Supporting Information). With uniform Fj and a stationary Ds front, cells exhibited a broadly similar but weaker polarization pattern than that shown in Fig 5. However, when Fj was absent, Dachs polarization was very weak throughout the disc. This suggests that the Ds front is sufficient to induce distal Dachs localization in nearby cells, but the degree of Dachs polarization is greater when Fj is present and greater still when Fj is spatially graded.

Both a stationary and a moving front produced a pattern of Dachs localization near the front that is broadly similar to that seen experimentally in wild-type discs [7, 26] (Figs 3 and 5). The average magnitude of Dachs polarization showed little dependence on the movement of the front (S8 Fig in the Supporting Information). In both cases, Dachs in cells near the front tended to localize toward the distal side of each cell. However, in the case of a moving front, the average direction of Dachs localization in cells near the center of the disc was consistently distal, while Dachs was randomly localized in the center of a disc with a stationary front (Fig 5A–5D). Our model then predicts that the Ds front is a stronger polarizing cue for Dachs than the Fj gradient, and that cells can retain their Dachs polarization well after this front has moved away.

### The moving Ds front is sufficient to induce distal Dachs localization in the wing pouch

To differentiate further the effects of the graded Fj profile and the moving Ds front, we compared Dachs polarization at minute 2100 under a variety of conditions of Fj and Ds expression (Fig 4). In particular, we examined moving and stationary Ds fronts, and Fj that was either graded, absent, or uniformly overexpressed. With a normal (wild-type), graded Fj profile (Fig 4, left column), Dachs localization was the same as in Figs 3 and 4. When Fj was absent (Fig 4, center column), the overall magnitude of Dachs localization was much lower, but still distally oriented, in agreement with experimental results which found very weak Dachs asymmetry in a Fj mutant disc [26]. With uniformly overexpressed Fj (Fig 4, right column), the overall pattern of Dachs localization was similar to that with graded Fj, but with a slightly smaller average magnitude. In all cases, Dachs was distally localized in cells near the front. However, when the front moved, Dachs was also distally localized in the cells near the center of the disc that the front had previously passed over. These results suggest that a moving Ds front is sufficient to induce distal Dachs localization in the entire wing pouch, but the presence of Fj amplifies this effect, and a gradient of Fj amplifies it more. S14, S17, S18, S19, and S20 Figs in the Supporting Information examine the cases of uniform and absent Fj with moving and stationary Ds fronts in greater detail.

The Fj gradient alone is also sufficient to give rise to weak polarization. S15 Fig shows that when Ds expression is uniform and Fj is graded, some weak distal Dachs localization is still observed. However, when both Fj and Ds are expressed uniformly as in S16 Fig, Dachs localization is randomly oriented. Thus, the gradient (slope) of the Fj concentration is less important for polarization. We investigate the Fj profile’s effect on Dachs localization in finer detail in S18 Fig in the Supporting Information.

### Model for growth and polarization is consistent with experimental results

**Fig 6:**
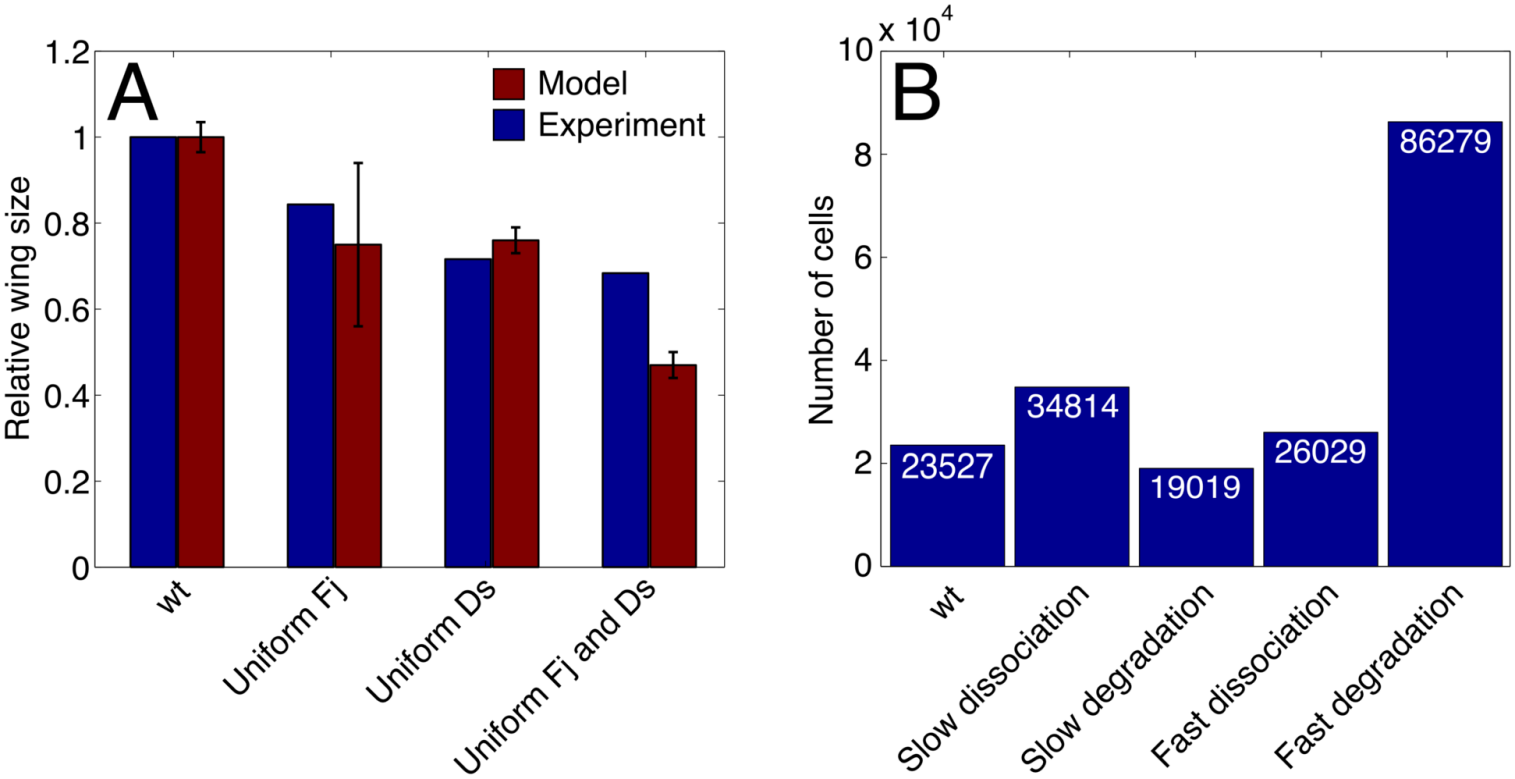
The number of cells in the wing disc under various conditions and parameter values. In each case, the same starting set (in terms of cell sizes and locations) of 1000 cells was simulated for 2880 time steps, corresponding to 48 hours of real time, the approximate length of the third instar. (A) Each bar marked “Model” represents the relative size of the disc compared to wild-type (the number of cells in that disc divided by the number of cells in the wild-type disc) averaged over 20 runs with the same conditions. (Because the size at which a cell divided and the rates at which it formed and dissolved Ft-Ds bonds were not completely deterministic as described in Section 4 of the Supplemental Text, simulation runs under the same conditions were not identical.) These relative sizes were compared to the corresponding experimental results (red bars) on the sizes of adult wings relative to the size of the wild-type wing [7]. Here, “uniform Fj”, “uniform Ds”, and “uniform Fj and Ds” are discs that uniformly express high levels of Fj, Ds, or both. Other than the stated variations, each run used the same set of parameter values as described in Section 10 of the Supplemental Text. The error bars on the simulation results are less than 1% of the height of the bars shown in the plot and therefore are not shown. The error bars on the experimental results correspond to one standard deviation. (B) The number of cells in the wing disc using different parameter values. As in panel A, the same set of 1000 cells was simulated for 2880 time steps, or 48 hours. Each bar represents the average number of cells in the disc over 20 of these runs. In “slow dissociation”, the dissociation rate of Ft-Ds bonds was decreased by a factor of 10 to 8x10-3 per time step (from the wild-type value of 0.08 per time step). In “slow degradation”, the degradation rate of free Ft and Ds was decreased by a factor of 10 to 8x10-3 per time step (from the wild-type value of 0.08 per time step). Similarly, in “fast dissociation”, the dissociation rate of Ft-Ds bonds was increased by a factor of 10 to 0.8 per time step. In “fast degradation”, the degradation rate of free Ft and Ds was increased by a factor of 10 to 0.8 per time step. As in panel A, each run used the same (wild-type) set of parameter values other than the stated variations.

Cell division and proliferation are important components of our model so it is worthwhile to check the model by comparing the growth of the wing disc to experimental outcomes under a variety of conditions. Since a number of experimental results on growth are given in terms of the overall wing disc size, Fig 6A summarizes our results in terms of the number of cells in the disc under various conditions. (Additional results on disc growth and polarization patterns are given in S7 Fig and section 8.1 of the Supplemental Text.) The growth of a wild-type disc was the control. All other parameters were fixed. Recall that in our model we assumed that the more asymmetric the bond distribution was, the faster the growth rate was. Cells in discs with uniformly expressed Fj or Ds tended to have more symmetrically distributed bonds and the disc undergrew, consistent with experiment [7, 18]. A disc that expressed both Fj and Ds uniformly had even more symmetrically distributed bonds and hence, undergrew more than a disc that only uniformly expressed one of the two, consistent with experiment [7]. Additional results that were qualitatively consistent with experiment, including results on the roughly uniform spatial pattern of cell proliferation, are presented in detail in Section 8 of the Supplemental Text.

### Parameter dependence

Several features of this model are dependent upon parameter values. For example, the degree to which cells retain their polarization after the Ds expression front has passed over them depends on the rate at which Ft-Ds bonds turn over (i.e., dissolve and are possibly replaced with different bonds) which is on the order of 1% per time step. While this rate of bond turnover is dependent on several parameters, it depends most strongly on the degradation rate of free Ft and Ds and the rate of dissociation of Ft-Ds bonds (note that only free, unbound, Ft or Ds undergoes degradation in our model, since endocytosis is usually necessary for the degradation of receptors). These rates affect the turnover rate of Ft-Ds bonds at a given interface as well as the ability of cells to respond to changing Ds expression. As a result, they must have a short time scale compared to that of the movement of the Ds front in order for Dachs localization to respond to the movement of the Ds front. On the other hand, they must have a long time scale compared to that of Ft and Ds expression in order to have a stable arrangement of Ft-Ds bonds and retain a memory of the passage of the Ds front.

The overall pattern of Dachs localization is fairly insensitive to changes in these degradation and dissociation rates (see S9–S12 Figs in the Supporting Information). However, the overall growth rate is more sensitive to these changes, as shown in Fig 6B. A detailed discussion of the dependence of bond asymmetry and cell proliferation on these degradation and dissociation rates is available in section 8.3 of the Supporting Information.

In Fig 6A the difference in disc undergrowth (compared to WT) between the “uniform Ds” and the “uniform Fj and Ds” cases is not as dramatic as that seen experimentally. However, relative wing disc sizes will depend in part on the length of the simulation, since growth is roughly exponential. Thus, longer growth times will lead to greater differences between wing disc sizes. In particular, we note that our simulation ends at the end of the third larval instar, while the experiment measures the areas of adult wings [7]. We could have adjusted our parameters to give better agreement with experiment for the growth under conditions of “uniform Ds” and “uniform Fj and Ds”, but this would have resulted in poorer agreement with other experimental results. In particular, many different parameters affect the relationship between Fj and Ds expression and the growth rate that we examine in Fig 6A. These include the rates of Fj and Ds overexpression when Fj or Ds concentrations are uniform, the concentration and spatial slope of Fj, the penalty to the growth rate for excess free Ft or Ds, the relationship between the Fj concentration and the Ft and Ds phosphorylation rates, the relative rates of bond formation for Ft and Ds with different phosphorylation states, and the contribution of the Ft-Ds bond distribution to the growth rate. Parameters are chosen to fit a number of experimental results regarding Ft-Ds binding and the overall growth rate of the disc, and changes would substantially affect our other results.

Some aspects of the model, including the dependence of the proliferation rate on the arrangement of Ft-Ds bonds and the retention of the overall bond arrangement after cell division, are dependent on the kinetics of Ft-Ds bond formation. In particular, these effects require the number of Ft-Ds bonds at an interface to reach a steady state on a time scale much shorter than a cell cycle. While the overall pattern of Dachs localization is once again fairly robust to changes in the time scale of Ft-Ds bond formation, the proliferation rate will be affected when cells frequently have at least one interface that is depleted of Ft-Ds bonds. We discuss this effect, along with the remaining parameters, in detail in Section 8.4 of the Supplemental Text.

## Discussion

### Fj and a moving Ds transition region are needed to produce widespread distalward Dachs polarization

Our simulations show that Dachs is still asymmetrically localized when Fj is uniform and its starting concentration and expression rate are both increased by approximately a factor of 10 compared to wild-type, though the magnitude of this polarization is very weak in the absence of Fj (S14 and S17 Figs in the Supporting Information show the distalward direction of polarization in the upper panels). Thus, our model predicts that the presence of Fj, more than its gradient, together with a moving Ds transition region, results in widespread distal polarization in the wing pouch. This is in contrast to previous models [12, 14] that relied on a linear gradient of Fj to achieve polarization. Furthermore, our model shows that a stationary front of Ds expression with a wild-type Fj gradient also produces asymmetric Dachs localization in much of the wing pouch, suggesting that either the moving Ds front or the Fj profile alone can give rise to the polarization seen experimentally. This redundancy appears to confer some degree of robustness to variations in Ds and Fj expression. To compare our model and previous models, it would be useful to do an experiment that assesses the polarization in a disc with uniform Fj levels.

### The distribution of Ft-Ds bonds around a cell constitutes a “memory” of local expression patterns

In our model the Ft-Ds bonds that each cell forms with its neighbors constitute the cell’s “record” of previous patterning events in the disc. The distribution of these bonds reflects not only current Ft and Ds availability, but also the entire history of Ft and Ds availability in the cell. The Ft-Ds bond asymmetry persists after the transition region or front has moved beyond the cell. For this reason, the growth rate and polarization direction can be maintained throughout the wing pouch to a much greater extent than one would expect from the currently existing expression patterns. Furthermore, because a cell’s Ft-Ds bonds are the result of its entire history, each cell engages in a form of temporal averaging when determining its polarization, reducing its sensitivity to noise in the Ft pathway signaling.

To understand the mechanism underlying this memory, note that all the processes described earlier (Ft/Ds/Fj expression and degradation, bond formation and dissolution, phosphorylation and dephosphorylation) happen at finite rates, with turnover times on the order of hours, comparable to the time scales of the movement of the front and of the cell cycle. This means that the asymmetric arrangement of bonds around a cell can persist well after the cell is no longer near the front, suggesting that the net dissociation rates of the Ft-Ds bonds should be significantly smaller than the rate at which the Ds front propagates. We examine this time scale dependence further in section 8.3 of the Supplemental Text.

### Bonds remain asymmetrically distributed over multiple cell cycles

**Fig 7:**
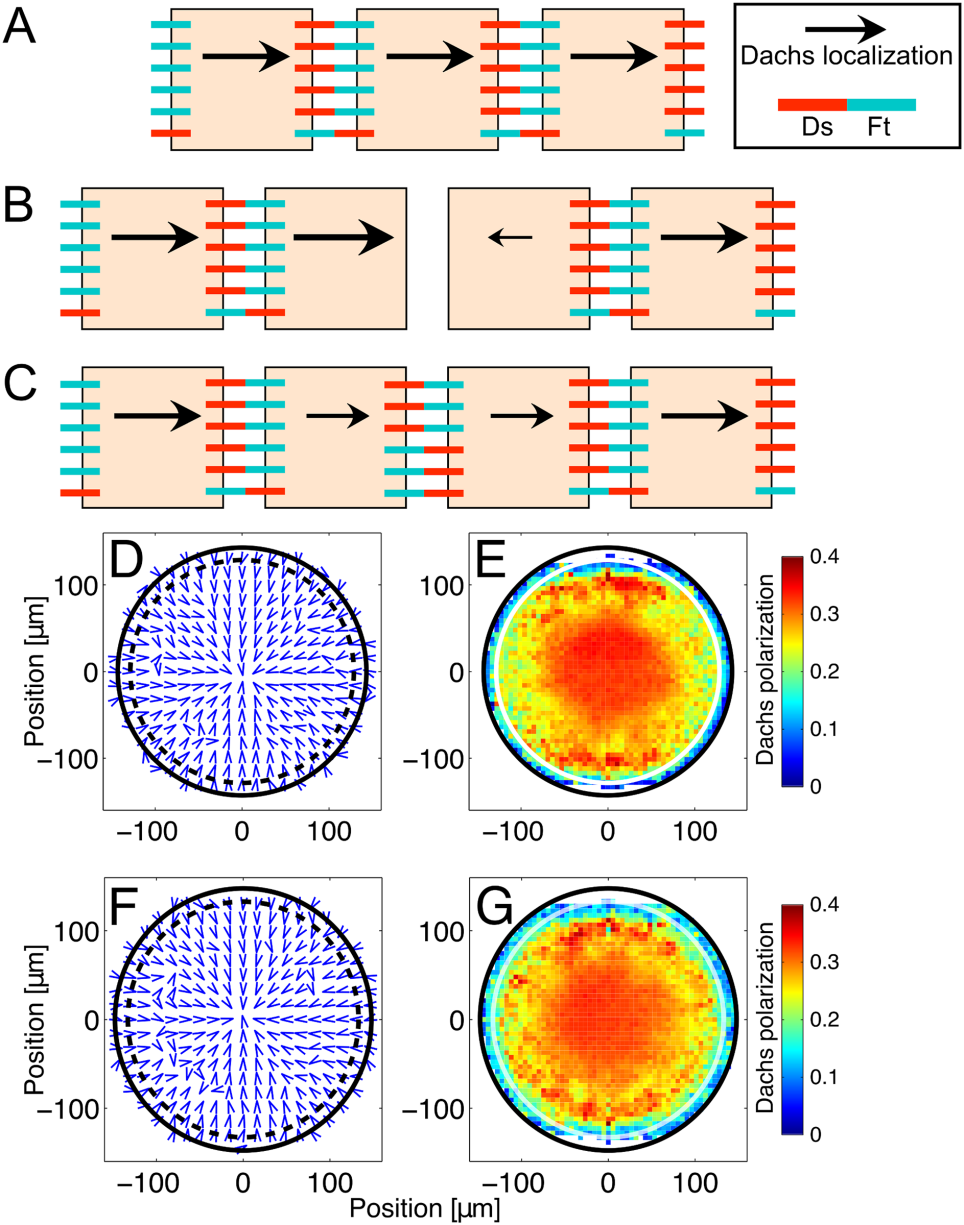
(A-C) Cartoon illustrating how cells can recover their polarization after dividing. Individual cells (squares) are represented, along with their bound Ft (red) and Ds (blue-green). The magnitude and direction of Dachs localization are represented by the length and direction of the arrow (vector) on each cell. (A) Three cells, assumed to have similar rates of Fj and Ds expression, have highly asymmetric distributions of Ft-Ds bonds. Dachs (black arrows) localizes to the side of each cell with the fewest bound Ft, which is the right side in this figure. (B) The central cell divides, forming two cells with a new interface between them. This newly formed interface has no Ft-Ds bonds; the changed bond distribution temporarily disrupts the Dachs localization in the two daughter cells. (C) Over time, Ft and Ds form bonds at the new interface. The two cells express Fj and Ds at similar rates and the formation of new bonds is independent of the polarity of existing bonds, so new Ft-Ds bonds are equally likely to form with either polarity at this interface. However, the overall pattern remains the same; both cells now have less bound Ft on their right interface, so Dachs localizes to the right side of each cell. Note that in this one-dimensional diagram, we depict cells as having only two interfaces, suggesting that a second cell division would disrupt the asymmetry entirely. However, in two dimensions, cells have an average of roughly 6 neighbors [49, 50], which would allow the asymmetry to persist for several cell cycles, longer than the third instar (which lasts roughly 48 hours and 6 cell cycles). (D, E) The direction and magnitude of Dachs polarization in a disc that has been simulated for roughly 120 hours and 10 cell cycles. The Fj profile is graded, as in “wild-type” runs of the simulation. The disc retains its overall pattern of distalward Dachs localization. (F, G) The direction and magnitude of Dachs polarization in a disc with a uniform Fj profile that has been simulated for roughly 120 hours and 10 cell cycles. The Dachs polarization pattern is slightly weaker in some areas but broadly similar to panels D-E, suggesting that the Fj gradient is not necessary for Dachs polarization in the long term. If cell proliferation were substantially faster compared to the rate of Ft-Ds bond formation, we would expect the bond asymmetry to be lost earlier.

How do cells retain their polarization over multiple cell cycles? While Ft-Ds bonds are being formed and dissolved, cells are constantly growing and dividing, forming new interfaces between adjacent cells. In our model, after a cell divides, adjacent cells have new interfaces that initially have no Ft-Ds bonds, so the polarization of recently divided cells is potentially disrupted. Fig 7 illustrates what happens to the arrangement of Ft-Ds bonds around a newly divided cell. Before division (Fig 7A), cells all have similarly polarized Ft-Ds bonds. These cells are assumed to be far from the transition region, and so express Ft, Ds, and Fj at similar rates. When a cell divides, it creates a new interface between the two daughter cells with no bonds on it, temporarily disrupting the polarization of the daughter cells (Fig 7B). Over time, however, new bonds of both polarities form at the interface, and the overall pattern of Ft-Ds bond polarization is restored (Fig 7C). Bonds of both polarities can form at the new interface because the formation of new Ft-Ds bonds in our model is independent of any existing bonds. However, if Ft-Ds binding exhibits cooperativity [27], bond formation in a newly divided cell will tend to follow the pattern of existing bonds on that cell, potentially allowing cells to recover their Dachs localization even faster. We also examined the results of a long run of the simulation covering 120 hours, more than double the duration of the third instar (roughly 48 hours and 6 cell cycles) under otherwise wild-type conditions (Fig 7D and 7E) and using a uniform Fj profile (Fig 7F and 7G). In both cases, the disc largely retains its pattern of Dachs localization, suggesting that the graded Fj profile is not necessary for long term Dachs polarization. However, this result depends on the rate of Ft-Ds bond formation being fast compared to the rate of cell proliferation. When Ft-Ds bond formation is substantially slowed, cells at the center of the disc rapidly lose their Dachs polarization (see S13 Fig in the Supporting Information).

### Experiments that could test the model and measure the parameters

The most direct test of our model’s predictions would be to measure the magnitude and direction of Dachs localization over time. In particular, we would expect the pattern of Dachs polarization to correlate closely with the pattern of Ds expression, with strong Dachs polarization near the boundary of Ds expression (i.e., the edge of the wing pouch) and relatively weak Dachs polarization outside it. We would then expect the degree of Dachs polarization in any individual cell to change over time; for example, a cell might begin the third instar with very little Dachs polarization, have its polarization increase dramatically as the Ds front approaches it, then have its polarization decrease slightly as the Ds front moves on. Moreover, we would expect the overall degree of Dachs polarization to be lessened in the absence of graded Fj or Ds, and undetectable when neither Fj nor Ds is graded [26]. A different result would suggest that factors other than the Fj and Ds profiles play a role in Dachs polarization.

Some of the parameters of the Ft-Ds-Fj interaction described above may be measurable experimentally. For example, to measure the rate of Ft-Ds bond dissociation and investigate the relationship between bond asymmetry and growth rate, one could halt the expression of either Ft or Ds in a clone of an otherwise wild-type disc. The expression of a gene can be induced in a single clone via, for example, treatment with a drug [43]. If this gene coded for an RNA that inhibited Ft or Ds expression (similar to those used in [8]), the resulting clone would have its Ft or Ds expression rates dramatically lowered after the drug was administered. For example, consider such a clone of cells in which Ft expression is suddenly stopped. In our model, this would drastically slow the formation of new Ft-Ds bonds, since no new Ft would be expressed and existing Ft would continue to degrade. We would therefore expect the overall number of bonds to decline over time, but by assumption, not the bond distribution asymmetry or Dachs polarization. According to our model, such an experiment would cause the clone’s growth rate to stay fairly constant (or even decrease due to the new excess of free Ds), until suddenly the growth rate would increase when cells lose all of their bonds. A similar reversal in growth rate is seen experimentally; discs with reduced Ft expression undergrow [27] but discs mutant for Ft overgrow [46, 51–53]. Experimentalists could also potentially alter the degradation rates of free Ft and Ds to examine their effects on the distribution of Ft-Ds bonds and cell growth.

### Considerations that go beyond the model

In our simulations we determined the spatial symmetry of the distribution of Ft-Ds bonds by counting the number of bonds a cell forms with each of its neighbors. An actual cell could detect the spatial distribution of bonds via mechanotransduction. Although bulk mechanical forces between cells do not interact with the Ft pathway in our model, Ft-Ds bonds could be involved in mechanical signaling by mechanically linking the cytoskeletons of adjacent cells. There is evidence that mechanical forces play a role in the Ft pathway. For instance, Dachs is a myosin and so may be directly involved in mechanotransduction. In addition, Yorkie, which is downstream of Dachs in the Ft pathway, is homologous to YAP (Yes-associated protein). Yorkie/YAP is a transcriptional coactivator associated with cell-ECM interactions and mechanotransduction [54, 55]. The Ft pathway may thus be involved in transducing the mechanical stresses that a cell experiences from its neighbors via Ft-Ds bonds. Other models have also suggested that bulk mechanical compression decreases Ds activity and could be responsible for the movement of the Ds expression front [33]. In addition, the counteracting effects of morphogen signaling and mechanical compression on growth could give rise to a negative feedback loop that controls the final size of the disc [33, 37, 40, 56]. The possible interactions between mechanical stresses and the Ft pathway merit further study.

In our model, the movement of the front of Ds expression is driven solely by the increasing amplitude of the morphogen profile. We chose this mechanism largely for its simplicity; it makes few assumptions about other signaling pathways. However, recent studies have shown that discs with spatially uniform Dpp signaling still exhibit a pattern of Ds expression roughly resembling that in wild-type discs, with Ds expressed at high levels near the edge of the disc [57]. In our model, a disc with spatially uniform morphogen expression would uniformly express Ds at a low rate, and not have a transition region. This implies that other mechanisms besides morphogen signaling may underlie Ds expression, such as the recruitment of cells from the Ds-expressing periphery into the wing primordium [5], or differences in mechanical compression such as described above [33]. Fj expression is also affected by the levels of Ft, Dachs and Ds, suggesting the existence of feedback mechanisms within the Ft pathway. In particular, Fj expression is upregulated in Ft mutant clones [6] and downregulated in Dachs mutant clones [6]. Similarly, clones that overexpress Ds reveal nonautonomously elevated levels of Fj near their boundaries, both inside and outside their boundaries [34], providing further evidence for possible feedback mechanisms.

In our model, the magnitude and direction of Dachs localization are outputs that do not directly affect other parts of the model. However, evidence shows that Dachs localization can affect the orientation of cell divisions [48]. (In our model, the orientation of cell divisions has very little effect on the overall results; see section 2.2 of the Supplemental Text.) Although cells in the wing disc prior to pupariation do not substantially rearrange or sort [49], Dachs has been shown to affect the rearrangement of cells in the thorax during the pupal stage [9]. The relationship between Dachs localization, cell division patterns, and cell rearrangement may present an interesting challenge for future modeling studies. Our model also shows that complementary patterns of morphogen and Ft pathway signaling can lead to roughly uniform growth in the wing pouch (i.e., within the wake of the expression front); see S20 and S21 Figs and sections 8.8–8.9 of the Supplemental Text.

## Conclusions

We have developed a model of the *Drosophila* wing disc showing how cell polarization in the form of an asymmetric distribution of Ft-Ds bonds survives cell division. Experimentally, the cells of the *Drosophila* wing have asymmetrically distributed Ft-Ds bonds with their neighbors [8, 26]. Our model indicates that this distribution is due to more than just the graded slope of Fj in agreement with experiment [26] which found that Dachs polarity is lost when Ds is uniform and Fj is absent, but not when just Fj is absent. In our model, it is the movement of the Ds expression boundary with respect to individual cells that is a major contributor to Dachs polarity. Even though Ft and Ds have a fairly flat spatial concentration profile in most of the wing pouch [5, 15–21], the Ds concentration profile is not static. The boundary of the wing pouch (or “transition region”), where Ds has a steep gradient, expands outward over time [5]. In our model, cells in the wake of this expanding front are more strongly polarized than cells that have never had the front pass over them. Furthermore, we propose that cells retain their Ft-Ds bond asymmetry after the front has passed and after cell division by quickly replenishing Ft-Ds bonds at the newly formed cell-cell interface. The movement of the front in our model, together with the presence of Fj, allows cells throughout the wing primordium to experience roughly uniform growth and patterning in the presence of dynamic, nonuniform signals. By integrating this mechanism into a simple model for proliferation, we obtain results consistent with a number of experimental observations of disc size and polarization [7, 8, 18, 26].

We propose that the wing disc is an example of a biological system that depends not only on the current state of gene expression, but also on its history and on multiple interacting factors that change over time. There are other examples of this, e.g., somites, or body segments, of vertebrates are formed by a propagating wave front that passes over cells undergoing oscillatory changes in their gene expression [58]. The fate of each cell depends on its state when the front reaches it with the result being a periodic spatial pattern of somites that persists after the front has passed. Thus, dynamics in tissue size, cell number, signaling levels, and expression patterns are important during development.

## Supporting information

S1 Text In this supplement we describe the methods used to measure the spatial profiles of Ds and Fj. We also discuss the details of the simulation, including the mechanical interactions between cells, the rules governing cell growth and division, and the algorithms for forming Ft-Ds bonds and for determining the neighbor list. Finally, we present additional results on cell polarization and growth.
(PDF)

S1 Fig The starting population of cells with its Delaunay triangulation shown. Individual cells (orange circles) are drawn at roughly 1/2 actual size here, to show the neighbor relationships (blue lines). These neighbors are not all used for purposes of mechanical interactions or Ft-Ds binding; other criteria also apply. Also note that the density of cells varies with radial position because adjacent cells attract, causing the center of the disc to be compressed compared to the periphery. A similar pattern of compression is observed experimentally (Aegerter-Wilmsen *et al*., Development 2012). Note that cells near the edge of the disc may have neighbors in the Delaunay triangulation that are not truly adjacent (i.e., the distance between them is larger than the typical size of a cell). The locations of these wider gaps in the circle’s perimeter are simply due to chance; the overall density of cells depends only on radial position.
(EPS)

S2 Fig The acceleration of a cell as a function of its distance from its neighbor d_ij_/d_eq,ij_. Here, d_ij_ is the distance between the centers of the cells and d_eq,ij_ is the sum of the radii of the cells. Cells attract (negative acceleration) when they have a gap between them (d_ij_ > d_eq,ij_) and repel (positive acceleration) when they overlap (d_ij_ < d_eq,ij_). Note that the acceleration is zero when the cells are just touching (d_ij_ / d_eq,ij_ = 1) and when the cells are very far from one another (d_ij_/d_eq,ij_ → ∞).
(EPS)

S3 Fig Ft-Ds bonds on adjacent cells. The more uneven the arrangement of these bonds, the faster the cell will grow. To calculate the minimum fraction of bound Ds on the central cell, we divide the smallest number of bound Ds shared with any of the neighbors by the total number of bound Ds. In this case, the central cell has one bound Ds (orange) with the bottom cell, the fewest of all its neighbors. Because the cell has a total of 12 bound Ds, the minimum fraction would then be 1/12. We then multiply by the number of neighbors to give an adjusted minimum fraction of 6/12 or 1/2. Similarly, the minimum fraction of bound Ft would be 6/7. Note that in the case of perfectly evenly distributed bonds (for this cell, 2 on each neighbor), this quantity would be equal to 1. Similarly, if the cell lacks bonds with at least one of its neighbors, the adjusted minimum fraction would be 0.
(EPS)

S4 Fig An example of a Delaunay triangulation. The four points are connected by edges (line segments) to form a mesh of triangles. Note that the circumscribed circle of each triangle contains no points besides the three used to construct the triangle: the blue circle does not contain the bottom point, and the purple circle does not contain the top point. Only pairs of points connected by edges are added to the neighbor list. In this example, the top and bottom points would not be considered neighbors, but every other pair of points would be.
(EPS)

S5 Fig The Delaunay triangulation divide-and-conquer algorithm, based on Guibas and Stolfi, *ACM T Graphic* 1985. (1)The base edge AB is chosen (dotted line). Note that all other vertices lie to one side of (in this case, above) this edge. (2) Candidate vertices C and D are chosen (circled in blue). C is the next neighbor of A going counterclockwise from the base edge, and D is the next neighbor of B going clockwise from the base edge. However, the next right-side candidate E lies within the circle (green) circumscribing the triangle ABD. (3) The edge BD is deleted and E becomes the new right-side candidate. The circle circumscribing the triangle ABC does not contain the candidate E (while the circle circumscribing ABE would contain C). (4) The candidate C is then chosen, BC is drawn and becomes the new base edge, and the process is repeated until neither side returns a suitable candidate. (5) The completed triangulation.
(EPS)

S6 Fig Partitioning the neighbors of a newly divided cell. Here, each of the labeled vertices represents the center of a cell, and the line segments correspond to neighbor pairs in the triangulation. Cell A has six neighbors, labeled B through G (left diagram). A divides and gives rise to H. The positions of these two cells, along with those of A's neighbors, are passed to the triangulation algorithm. The resulting neighbors may correspond to the preexisting set of neighbors and be retained (shown in black), or they may represent one of the mother cell's neighbors being transferred to the daughter cell (F, blue) or shared between the two daughter cells (E and G, violet). In addition, there is a chance that the algorithm will consider two non-adjacent neighbors of the mother (B and D) to be neighbors of one another (green), even though they are not adjacent in the context of the entire disc. Such neighbor pairs will be expunged before this set of neighbors is reintegrated into the main neighbor list.
(EPS)

S7 Fig The number of cells in the wing disc under various conditions. In each case, the same starting set of 1000 cells was simulated for 1700 time steps. Each bar represents the average number of cells in the disc over 20 runs with the same conditions. (Because the size at which a cell divides and the rate at which it forms and dissolves Ft-Ds bonds are not completely deterministic, simulation runs under the same conditions are not identical.) Here, “ft-", “ds-", “ft- ds-", and “fj-" refer to discs missing Ft, Ds, both Ft and Ds, or Fj, respectively. “Uniform Fj", “uniform Ds", and “uniform Fj and Ds" are discs that uniformly express high levels of Fj, Ds, or both. “No Ft ICD" means that the discs' Ft has no intracellular domain; it binds to Ds but the resulting bonds are not counted by their cells, and there is no penalty to the growth rate for free Ft. “No Ft ECD" means that the discs' Ft has no extracellular domain; it cannot bind to Ds and is consequently always treated as free. “No morphogen" and “Uniform morphogen" refer to discs with no morphogen and uniformly high morphogen levels, respectively. (In every case, the standard deviation of the number of cells in the disc was less than 1% of the mean, so error bars are not shown.) Other than the stated variations, each run used the same set of parameter values.
(EPS)

S8 Fig The Ft-Ds bond asymmetry profile over time. The movement of the front allows a large portion of the tissue to be patterned. The asymmetry of bonds (vertical axis), averaged over 20 runs, is shown as a function of radial position (horizontal axis) in the case of both a moving (A-D) and a stationary (E-H) front. When the front (vertical black line) is moving, cells that it has passed over have more asymmetrically distributed bonds. (A-D) As the front moves, the region of greater bond asymmetry grows to encompass a larger fraction of the disc. (E-H) When the front is stationary, only cells near the front have asymmetrically distributed bonds, and this peak shrinks over time.
(EPS)

S9 Fig Dachs polarization when Ft-Ds dissociation is fast. k_dissoc_ is increased by a factor of 10 to 0.8 min^-1^, compared to 0.08 min^-1^ in wild-type. (Because the simulation normally only begins to calculate Dachs localization after time step 100 and records data every 10 time steps, we display the state of the disc at time step 110.) All other parameters are the same as in wild-type (Fig 3 in the main text). The arrows in panels A-D represent spatial averages of cell polarization; they point to the side of the cell with the least amount of bound Ft. Panels E-H show the magnitude of Dachs polarization in the wing disc; blue represents weaker polarization, red stronger polarization. Dachs localization is a vector that varies from zero, for an isotropic distribution of Dachs, to unity, if Dachs is all on one side. The solid black circle is the approximate edge of the entire disc, while the dotted black circle (A-D) and white circle (E-H) represent the Ds expression front. This front moves radially outward over time, encompassing an increasing fraction of the whole disc.
(EPS)

S10 Fig Dachs polarization when Ft-Ds dissociation is slow. (A-H) k_dissoc_ is decreased by a factor of 10 to 8x10^-3^ min^-1^, compared to 0.08 min^-1^ in wild-type. The arrows in panels A-D represent spatial averages of cell polarization; they point to the side of the cell with the least amount of bound Ft. Panels E-H show the magnitude of Dachs polarization in the wing disc; blue represents weaker polarization, red stronger polarization. Dachs localization is a vector that varies from zero, for an isotropic distribution of Dachs, to unity, if Dachs is all on one side. The solid black circle is the approximate edge of the entire disc, while the dotted black circle (A-D) and white circle (E-H) represent the Ds expression front. This front moves radially outward over time, encompassing an increasing fraction of the whole disc. (I-P) k_dissoc_ is decreased by a factor of 2 to 0.04 min^-1^, compared to 0.08 min^-1^ in wild-type. Note the slightly decreased Dachs polarization compared to wild-type.
(EPS)

S11 Fig Dachs polarization when free Ft and Ds degradation is fast. k_d;Ft_ and k_d;Ds_ are both increased by a factor of 10 to 0.8 min^-1^, compared to 0.08 min^-1^ in wild-type. The arrows in panels A-D represent spatial averages of cell polarization; they point to the side of the cell with the least amount of bound Ft. Panels E-H show the magnitude of Dachs polarization in the wing disc; blue represents weaker polarization, red stronger polarization. Dachs localization is a vector that varies from zero, for an isotropic distribution of Dachs, to unity, if Dachs is all on one side. The solid black circle is the approximate edge of the entire disc, while the dotted black circle (A-D) and white circle (E-H) represent the Ds expression front. This front moves radially outward over time, encompassing an increasing fraction of the whole disc. Missing arrowheads in panels A-D (darkest blue regions in panels E-H) represent regions where the cells have no bound Ft or Ds and thus no net polarization.
(EPS)

S12 Fig Dachs polarization when free Ft and Ds degradation is slow. k_d;Ft_ and k_d;Ds_ are both decreased by a factor of 10 to 8x10^-3^ min^-1^, compared to 0.08 min^-1^ in wild-type. All other parameters are the same as in wild-type (Fig 3 in the main text). The arrows in panels A-D represent spatial averages of cell polarization; they point to the side of the cell with the least amount of bound Ft. Panels E-H show the magnitude of Dachs polarization in the wing disc; blue represents weaker polarization, red stronger polarization. Dachs localization is a vector that varies from zero, for an isotropic distribution of Dachs, to unity, if Dachs is all on one side. The solid black circle is the approximate edge of the entire disc, while the dotted black circle (A-D) and white circle (E-H) represent the Ds expression front. This front moves radially outward over time, encompassing an increasing fraction of the whole disc.
(EPS)

S13 Fig Dachs polarization when the rate of Ft-Ds binding is reduced by a factor of two. The weight parameters described in Section 3.3 are reduced to 0.025 for Ft-Ds bonds, 0.35 for FtP-Ds, 0.025 for Ft-DsP, and 0.1 for FtP-DsP. (The normal values are 0.05, 0.7, 0.05, and 0.2, respectively.) Other details are the same as in S9 Fig.
(EPS)

S14 Fig Dachs polarization produced when Fj expression is spatially uniform. The direction (panels A-D) and magnitude (panels E-H) of Dachs polarization at 110 (A, E), 700 (B, F), 1400 (C, G) and 2100 (D, H) time steps (minutes) after the beginning of a single run are shown. All other parameters are the same as in wild-type (Fig 3 in the main text). The arrows in panels A-D represent spatial averages of cell polarization; they point to the side of the cell with the least amount of bound Ft. Panels E-H show the magnitude of Dachs polarization in the wing disc; blue represents weaker polarization, red stronger polarization. Dachs localization is a vector that varies from zero, for an isotropic distribution of Dachs, to unity, if Dachs is all on one side. The solid black circle is the approximate edge of the entire disc, while the dotted black circle (A-D) and white circle (E-H) represent the Ds expression front. This front moves radially outward over time, encompassing an increasing fraction of the whole disc. Note that, as with wild-type discs, cells within the front exhibit some distal Dachs localization, while cells outside the front have more weakly localized Dachs.
(EPS)

S15 Fig Dachs polarization produced when Ds expression is spatially uniform. As in S14 Fig, the direction (panels A-D) and magnitude (panels E-H) of Dachs polarization at 110 (A, E), 700 (B, F), 1400 (C, G) and 2100 (D, H) time steps (minutes) after the beginning of a single run are shown. The black dotted line (A-D) and white circle (E-H) represent only the location of the critical concentration of morphogen, rather than any boundary of Ds expression. This radial position increases with time, encompassing an increasing fraction of the whole disc. All other details are the same as in wild-type (Fig 3 in the main text). The arrows in panels A-D represent spatial averages of cell polarization; they point to the side of the cell with the least amount of bound Ft. Panels E-H show the magnitude of Dachs polarization in the wing disc; blue represents weaker polarization, red stronger polarization. Dachs localization is a vector that varies from zero, for an isotropic distribution of Dachs, to unity, if Dachs is all on one side. The solid black circle is the approximate edge of the entire disc. In this case, Dachs is still on average localized to the distal (center-facing) side of cells in the wing pouch, but the magnitude of Dachs localization is smaller.
(EPS)

S16 Fig Dachs polarization produced when Fj and Ds expression are spatially uniform. The direction (panels A-D) and magnitude (panels E-H) of Dachs polarization at 110 (A, E), 700 (B, F), 1400 (C, G) and 2100 (D, H) time steps (minutes) after the beginning of a single run are shown. In this case, the black dotted line (A-D) and white circle (E-H) represent only the location of the critical concentration of morphogen, rather than any boundary of Ds expression. This radial position increases with time, encompassing an increasing fraction of the whole disc. All other details are the same as in wild-type (Fig 3 in the main text). The arrows in panels A-D represent spatial averages of cell polarization; they point to the side of the cell with the least amount of bound Ft. Panels E-H show the magnitude of Dachs polarization in the wing disc; blue represents weaker polarization, red stronger polarization. Dachs localization is a vector that varies from zero, for an isotropic distribution of Dachs, to unity, if Dachs is all on one side. The solid black circle is the approximate edge of the entire disc. Note that Dachs throughout the disc, both inside and outside the boundary, is randomly localized with a low average magnitude of polarization, since all cells have similar amounts of Ft, Ds, and Fj.
(EPS)

S17 Fig Dachs polarization produced when Fj is absent. The direction (panels A-D) and magnitude (panels E-H) of Dachs polarization at 110 (A, E), 700 (B, F), 1400 (C, G) and 2100 (D, H) time steps (minutes) after the beginning of a single run are shown. The black dotted line (A-D) and white circle (E-H) represent the location of the critical concentration of morphogen and the Ds front. This front moves radially outward over time, encompassing an increasing fraction of the whole disc. All other details are the same as in wild-type (Fig 3 in the main text). The arrows in panels A-D represent spatial averages of cell polarization; they point to the side of the cell with the least amount of bound Ft. Panels E-H show the magnitude of Dachs polarization in the wing disc; blue represents weaker polarization, red stronger polarization. Dachs localization is a vector that varies from zero, for an isotropic distribution of Dachs, to unity, if Dachs is all on one side. The solid black circle is the approximate edge of the entire disc. The overall magnitude of polarization is very small compared to a wild-type disc, although Dachs still localizes to the distal side of cells near the front. This suggests that the presence of Fj is necessary for the production and retention of polarization.
(EPS)

S18 Fig Dachs localization under different conditions of Fj expression. We examine the average magnitude (panels A, C, and E) and direction (panels B, D, and F) of Dachs localization in all cells of the disc at time step (minute) 2100 of the simulation, using different Fj profiles. The magnitude of Dachs polarization is defined as one minus the minimum fraction of bound Ft, as in previous figures, and is always between 0 and 1. The direction of Dachs polarization is measured with respect to the P-D (radial) axis, from 0 (proximal direction) to π (distal direction) radians (B, inset). Error bars represent one standard deviation. Except for the single parameter varied (Fj slope, minimum, and uniform level), all parameters are the same as in wild-type. (A-B) Dachs polarization when the Fj slope is equal to 0, 2.5x10^-3^, 1x10^-2^, 2.5x10^-2^ (the wild-type value), 0.1, and 0.25 μm^-1^. The minimum value of Fj is held constant at 0.1. (C-D) Dachs polarization when the minimum value of Fj is equal to 0, 1x10^-2^, 4x10^-2^, 0.1 (the wild-type value), 0.4, and 1. The slope of Fj is held constant at 2.5 x 10^−2^ μm^-1^. (E-F) Dachs polarization when Fj is expressed uniformly with a concentration of 0, 0.4, 1, 4, and 10. Note that the magnitude of Dachs localization is lowest when Fj levels are low (i.e., on the order of 0.1 or lower) throughout the disc, but is otherwise robust to changes in the Fj profile.
(EPS)

S19 Fig Dachs polarization produced when the Ds expression front is stationary and Fj is expressed uniformly. The direction (panels A-D) and magnitude (panels E-H) of Dachs polarization at 110 (A, E), 700 (B, F), 1400 (C, G) and 2100 (D, H) time steps (minutes) after the beginning of a single run are shown. The black dotted line (A-D) and white circle (E-H) represent the location of the Ds front. The relative radial position of the front does not change during a run. All other details are the same as in Fig 5 in the main text. The arrows in panels A-D represent spatial averages of cell polarization; they point to the side of the cell with the least amount of bound Ft. Panels E-H show the magnitude of Dachs polarization in the wing disc; blue represents weaker polarization, red stronger polarization. Dachs localization is a vector that varies from zero, for an isotropic distribution of Dachs, to unity, if Dachs is all on one side. The solid black circle is the approximate edge of the entire disc. Although the average magnitude is smaller, the overall pattern of polarization is similar to that in Fig 5 in the main text, with greater Dachs localization among cells near the Ds expression front.
(EPS)

S20 Fig Dachs polarization produced when the Ds expression front is stationary and Fj is absent. The direction (panels A-D) and magnitude (panels E-H) of Dachs polarization at 110 (A, E), 700 (B, F), 1400 (C, G) and 2100 (D, H) time steps (minutes) after the beginning of a single run are shown. The black dotted line (A-D) and white circle (E-H) represent the location of the Ds front. The relative radial position of the front does not change during a run. All other details are the same as in Fig 5 in the main text. The arrows in panels A-D represent spatial averages of cell polarization; they point to the side of the cell with the least amount of bound Ft. Panels E-H show the magnitude of Dachs polarization in the wing disc; blue represents weaker polarization, red stronger polarization. Dachs localization is a vector that varies from zero, for an isotropic distribution of Dachs, to unity, if Dachs is all on one side. The solid black circle is the approximate edge of the entire disc. In the absence of Fj, cells near the Ds front have only a very weak average distal Dachs localization, suggesting that the presence of Fj is necessary to give rise to substantial polarization.
(EPS)

S21 Fig Dachs polarization produced when the length of the time step is doubled to 2 minutes. The direction (panels A-D) and magnitude (panels E-H) of Dachs polarization at 110 (A, E), 700 (B, F), 1400 (C, G) and 2100 (D, H) minutes (55, 350, 700, and 1050 time steps) after the beginning of a single run are shown. The black dotted line (A-D) and white circle (E-H) represent the location of the front. Other details are the same as in S9 Fig.
(EPS)

S22 Fig Dachs polarization produced when the length of the time step is halved to 30 seconds. The direction (panels A-D) and magnitude (panels E-H) of Dachs polarization at 110 (A, E), 700 (B, F), 1400 (C, G) and 2100 (D, H) minutes (220, 1400, 2800, and 4200 time steps) after the beginning of a single run are shown. The black dotted line (A-D) and white circle (E-H) represent the location of the front. Other details are the same as in S9 Fig.
(EPS)

S23 Fig Growth rates due to morphogen signaling and Ft-Ds bond asymmetry form complementary patterns. The horizontal axis is the absolute position of cells within the disc, measured from the center (left) to the edge (right). The vertical axis shows the actual growth rate in terms of the ratio by which a cell’s radius increases in one hour (black), along with the value of the growth rate due only to bond asymmetry (blue) or morphogen signaling (green), averaged over 20 runs of the simulation. The transition region (vertical black line), which corresponds to the border of the prospective wing blade, moves radially outward over time, so that the wing primordium takes up an increasing fraction of the disc. Cells that have had this front pass over them recently (i.e., cells near the front) have the highest growth rate due to bond asymmetry. Cells near the disc’s center experience the most morphogen signaling and the highest associated growth rate. The two patterns are complementary, such that their product (essentially, the overall growth rate) is more uniform throughout the wing pouch than either profile alone.
(EPS)

S24 Fig Growth rate when the Ds expression front is stationary. The horizontal axis is the radial position of cells in the disc, measured from the disc's center. The vertical axis represents the growth rate in terms of the ratio by which a cell's radius increases in one hour (black), and the growth rates due solely to bond asymmetry (blue) and morphogen signaling (green), averaged over 20 runs. When the Ds expression front (vertical black line) is stationary in terms of its relative position between the cell's center and edge, the effect of bond asymmetry is most prominent near the front, forming a small peak (seen at approximately 85% of the disc's overall radius). This effect fades over time due to the adaptation mechanism, since the cells near the front are experiencing a fairly constant amount of bond asymmetry. The resulting pattern of cell growth is then dominated by morphogen signaling, rather than by counterbalancing patterns of morphogen signaling and bond asymmetry.
(EPS)

S25 Fig Bond asymmetry in a disc with a clone whose morphogen signaling is elevated. The asymmetry in the disc with the clone (red dots), 200 time steps (i.e., 200 minutes) after induction, is compared to bond asymmetry in a wild-type disc without a clone (blue diamonds). In separate runs, circular clones concentric with the disc were induced at the same time step with different radii (A: 0.2R, B: 0.4R, C: 0.6R, and D: 0.8R where R is the radius of the disc). Each panel is taken from a single run. This allows the disc to be roughly radially symmetric in terms of morphogen, Ft, Ds, and Fj expression. The location of the Ds expression front is indicated by the dashed vertical blue line. The horizontal axis indicates the distance in cells from the clone boundary (vertical black line); negative values are inside the clone (shaded area), and positive values are outside the clone (unshaded area). The center of the disc is at the left edge of each plot. The vertical axis is the average degree of asymmetry in bound Ft among all cells with a given distance from the clone boundary. We measure asymmetry as one minus the adjusted minimum fraction of bound Ft; thus, an asymmetry of 0 indicates of perfectly even distribution of bound Ft (leading to slower growth), while an asymmetry of 1 indicates at least one neighbor with no bonds (leading to faster growth). Note that the nonautonomous effect on bond asymmetry, both inside and outside the clone boundary, is most pronounced when the clone boundary is near or outside the Ds expression front (B, C, and D), where endogenous morphogen expression is lowest. Also note that the effect extends multiple cells from the boundary, particularly in D.
(EPS)

S26 Fig Cell growth rate in a disc with a clone whose morphogen signaling is elevated. The asymmetry in the disc with the clone (red dots) is compared to that in a wild-type disc (blue diamonds). Vertical blue dashed line is the transition region, or the boundary of the wing primordium. Vertical solid black line denotes the clone boundary. Growth rate is measured as the ratio of the cells new radius to the cells old radius in the previous time step. A time step corresponds to 1 minute of real time. Each panel is taken from a single run. Conditions are the same as in S25 Fig. Again, the nonautonomous effect on growth is strongest when the clone boundary is near the discs periphery (C and D), where the normal morphogen concentration is lowest. Note that the nonautonomous effect on growth extends over a distance involving multiple cells from the clones boundary. Also note the autonomous effect on growth within the clone compared to wild-type due to the increased degree of morphogen signaling.
(EPS)

S27 Fig Bond asymmetry near a Fj-overexpressing clone. In separate runs, circular clusters or “clones" of cells concentric with the disc were induced at the same time step with different radii (0.2 that of the disc in A, 0.4 in B, 0.6 in C, and 0.8 in D). These clones express Fj at a uniformly high rate, roughly 10 times the rate of Fj expression at the center of a normal disc. Other details are the same as in S25 Fig. The vertical axis is the average degree of asymmetry in bound Ft among all cells with a given distance from the clone boundary. An asymmetry of 0 indicates a perfectly even distribution of bound Ft among all of a cell's neighbors, and an asymmetry of 1 indicates at least one neighbor with no bonds. (A) Cells in a disc with a Fj-expressing clone (red dots) have similar amounts of bond asymmetry as those in a wild-type disc (blue diamonds) when the clone is within the Ds expression front (dashed vertical blue line), since this region of the disc already has a high Fj concentration. (B, C, D) When the clone boundary is outside the front, however, the steep discrepancy in Fj between adjacent cells leads to greater bond asymmetry both inside and outside the clone boundary.
(EPS)

S28 Fig Cell growth rate in a disc with a Fj-overexpressing clone. Conditions are the same as in S27 Fig, except that growth rate is plotted on the vertical axis. The growth rate here is expressed in terms of the ratio of a cell's radius at one time step to its radius at the previous time step. When the clone boundary (solid vertical black line) is outside the front (vertical dashed blue line), the growth rate is elevated near the clone's boundary on either side. This is because the Fj-overexpressing cells of the clone are next to the Ds-expressing cells of the disc's periphery.
(EPS)

S29 Fig Bond asymmetry near a Ds-expressing clone. All cells in the clone express 200 Ds per minute (compare to about 20 Ds per minute for peripheral cells in a normal disc). The asymmetry in the disc with the clone (red dots) is compared to that in a wild-type disc (blue diamonds). Again, the clone's radius is 0.2, 0.4, 0.6, and 0.8 that of the disc in A, B, C, and D, respectively. (A, B) Note that the nonautonomous effect on growth is strongest when the clone boundary (vertical black line) is in the central region of the disc, where endogenous Ds expression is lowest. In the interior of the clone (negative numbers) far from the boundary, bond asymmetry is low, since Ds is available uniformly within the clone. (C) When the clone boundary nearly coincides with the location of the front (vertical dashed blue line), the peak of bond asymmetry is smaller because cell-cell differences in Ds expression are less pronounced. (D) When the boundary of the clone is near the Ds expression front, bond asymmetry is uniformly low, because the disc then has uniformly high Ds expression.
(EPS)

S30 Fig Growth rate near a Ds-expressing clone. Conditions are the same as in S29 Fig, except that the vertical axis now represents growth rate. (A, B) The proliferation rate is fastest at the clone border when it lies within the wing primordium due to the increased bond asymmetry. (C) When the clone is large enough that its boundary is near the expression front, growth is slower because bonds are symmetric compared to those in a wild-type disc. (D) When the clone is large enough to span the entire wing pouch, Ds expression is uniformly high, leading to symmetrically distributed bonds and a low growth rate throughout the disc.
(EPS)

S31 Fig The distribution of neighbor numbers. Statistics were drawn from a wild-type disc at time step 1700.
(EPS)

S1 Table Parameters used in the morphogen profile and for specific disc genotypes. (PDF)

S2 Table Parameters used for cell growth, division, and interaction. (PDF)

S3 Table Parameters used in the expression and binding of Ft, Ds, and Fj. (PDF)
