# Supplementary material for: Expanding signaling-molecule wavefront model of cell polarization in the Drosophila wing primordium
Source: PLoS Comput Biol. 2017 Jul 3;13(7):e1005610. doi: 10.1371/journal.pcbi.1005610 (PMC5515495; doi:10.1371/journal.pcbi.1005610)
Supplement: S1 Text — We also discuss the details of the simulation, including the mechanical interactions between cells, the rules governing cell growth and division, and the algorithms for forming Ft-Ds bonds and for determining the neighbor list. Finally, we present additional results on cell polarization and growth. (PDF) [file pcbi.1005610.s001.pdf]

Supporting Information

In this supplement we discuss the details of the simulation, including the mechanical interactions between cells, the rules governing cell growth and division, and the algorithms for forming Ft-Ds bonds and for determining the neighbor list. Finally, we present additional results on cell polarization and growth.

1 Summary of simulation

Here is a overview of the operations that the simulation performs, at the beginning of a run and at each time step.

At the beginning of a run:

- Read in the locations and sizes of the starting population of cells from an external file. This file was originally produced by running the simulation starting with a single cell and allowing it to proliferate, then recording the locations and sizes (but not the Ft-Ds bond distributions or growth rates) of the resulting cells.
- Create the neighbor list for the starting cells (see section 7, "Building the neighbor list", and Figure S1).
- Calculate initial values of morphogen, Ft, Ds, and Fj for all cells, including phosphorylated and unphosphorylated subpopulations of Ft and Ds (see section 3.1, "Initial conditions and morphogen dynamics").
- Create a starting population of Ft-Ds bonds for all neighboring cells.

Then, at each time step:

- Handle mechanical interactions, growth and division. For each cell:
  - Update the position and velocity of the cell (see section 2, "Mechanical interactions").
  - Calculate the cell's growth rate and update its radius accordingly (see section 5, "Growth rate and adaptation").
  - Check to see if the cell divides this step (see section 2.2, "Cell division"). If so, create a new cell and update the neighbor list as well as the set of Ft-Ds bonds.
- Unbind a fraction of existing Ft-Ds bonds for all cells (see section 3.3, "Binding algorithm"). Binding is handled separately.
- Degrade a fraction of the unbound Ft and Ds on all cells.
- Dephosphorylate a fraction of the phosphorylated Ft and Ds on all cells.

- Degrade a fraction of the Fj in all cells.
- Express new Ft, Ds, Fj, and morphogen for each cell (see section 3.2, "Updating Ft, Ds, and Fj").
- Build new Ft-Ds bonds. For each cell:
  - Partition the cell's free Ft and Ds evenly among all its neighbors.
  - Check the corresponding free Ds and Ft on each of the cell's neighbors, and partition them similarly.
  - Form Ft-Ds bonds at each of the cell's interfaces. The probability of forming a particular bond depends on the phosphorylation states of its constituent Ft and Ds.

2 Mechanical interactions

In this section we present the details of our simulations of cells in the wing disc of the *Drosophila* larva. Because our model focuses on protein expression and dynamics on the scale of individual cells, the cell is the basic unit of our model. However, other models (e.g., [1]) have used the vertex as the basic unit, with cells as the polygons defined by these vertices. Such a model could conceivably also be used to study this system and would likely yield similar results.

In our model, cells exert forces on their neighboring cells, and can move accordingly. Each cell is represented mechanically by a point in two dimensions and a radius. The probability that a cell divides in a particular time step is given by a Hill function of its radius.

2.1 Intercellular forces

Cells move in response to the forces exerted by the neighboring cells. These forces prevent cells from excessive overlapping and from separating. One subroutine in the simulation is responsible for updating the acceleration of a cell given its location with respect to its neighbors. It steps through each neighbor of the given cell, calculating the distance between the centers of the cell and each neighbor. Thus, for two cells i and j whose centers are at (x_i, y_i) and (x_j, y_j), the distance between the centers is:

$$d_{ij} = \sqrt{(x_j - x_i)^2 + (y_j - y_i)^2} \quad \text{(S1)}$$

If two neighboring cells are separated by a gap, they will attract; if they are overlapping, they will repel (Figure S2). The equilibrium distance from a cell's center to the edge it shares with a given neighbor is equal to the cell's radius. Thus, the equilibrium separation between the centers of two neighboring cells with indices i and j is

$$d_{eq,ij} = r_i + r_j \quad \text{(S2)}$$

where r_i and r_j are the radii of the two cells; in other words, the cells are at equilibrium if their edges are just touching. If the distance between these two cells is greater than $d_{eq,ij}$, the two cells are separated by a gap and should attract. If the distance is less than $d_{eq,ij}$, the cells are overlapping and should repel. The force between the cells should be zero if the cells are just touching at the edge—in other words, if $d_{ij} = d_{eq,ij}$.

For the sake of simplicity, we treat all the cells as having the same mass (set equal to 1), so a cell's acceleration only depends on the sum of the forces on it. The base acceleration $\vec{a}$ is then calculated given the distance between the two cells. For adjacent cells with a gap between them (i.e., when $d_{ij} > d_{eq,ij}$), a Lennard-Jones-like acceleration is used for attraction:

$$|\vec{a}_i| = C_{gap}\left(\frac{d_{eq,ij}^6}{d_{ij}^7} - \frac{d_{eq,ij}^{12}}{d_{ij}^{13}}\right) \tag{S3}$$

where C_{gap} is a constant equal to 3×10^{-7} μm^2. This expression represents an attractive force that acts over small gaps ($d_{ij} \approx d_{eq,ij}$) and becomes negligible at longer distances ($d_{ij} \gg d_{eq,ij}$), since we would not expect cells that are far from one another to interact. Overlapping cells ($d_{ij} < d_{eq,ij}$) repel with a Hookean acceleration:

$$|\vec{a}_i| = C_{over}(d_{eq,ij} - d_{ij}) \tag{S4}$$

Here, C_{over} is a constant equal to 1×10^{-6} min^{-2}. The overall acceleration is plotted in Figure S2. Note that in both cases, acceleration is zero when $d_{ij} = d_{eq,ij}$. We use a Hookean force rather than a Lennard-Jones force for overlapping cells because the springlike force does not approach infinity for small distances between cells. Because cells proliferate and the simulation uses discrete time steps, there is a small chance that cells will suddenly substantially overlap without the opportunity to repel one another first. Furthermore, cells in the wing disc can deform when compressed, allowing adjacent cells to be closer than their equilibrium separation, so this approach is more realistic than using the Lennard-Jones expression for all separation distances.

The x and y components of this acceleration are calculated given the relative positions of the original cell and its neighbor. For example, if the cells are separated only in the x direction, then the acceleration will only have an x component. These components are then summed over all the cell's neighbors to yield the total acceleration of the cell at this time step. We call this acceleration $\vec{a}_{raw,i}$, where i is the index of each individual cell.

This acceleration, $\vec{a}_{raw,i}$, is used by a second subroutine, which updates the cell's velocity and actually moves the cell. First, a drag term γ (equal to 2×10^{-3} min^{-1}) is applied to the newly calculated acceleration to damp out oscillations:

$$\vec{a}_{n+1,i} = \vec{a}_{raw,i} - \gamma\vec{v}_i \tag{S5}$$

where $\vec{v}_i$ is the current velocity of cell i. This new acceleration $\vec{a}_{n+1,i}$ is then used along with the cell's acceleration $\vec{a}_{n,i}$ from the previous time step to find the new position and velocity using leapfrog integration [2]:

$$\vec{x}_{n+1,i} = \vec{x}_{n,i} + \vec{v}_{n,i}\Delta t + \frac{\vec{a}_{n,i}}{2}(\Delta t)^2 \tag{S6}$$

$$\vec{v}_{n+1,i} = \vec{v}_{n,i} + \frac{\vec{a}_{n,i} + \vec{a}_{n+1,i}}{2}\Delta t \tag{S7}$$

Here, $\vec{x}_{n+1,i}$ and $\vec{v}_{n+1,i}$ are the new values of position and velocity for cell i at step $n+1$, while $\vec{x}_{n,i}$ and $\vec{v}_{n,i}$ are the values of position and velocity for cell i at step n. The time step Δt is 1 minute. The cell is then updated with this new position and velocity.

2.2 Cell division

A cell grows by increasing its equilibrium size, pushing its neighbors back. A given cell's probability of dividing during any given time step is determined by its area:

$$P(\text{division}, i) = 1 - \left(\frac{1}{1 + \left(\frac{\pi {r_i}^2}{A_c}\right)^{\ell}} \right)^{\Delta t} \tag{S8}$$

where r_i is the radius of cell i, A_c is a critical area of 4π μm^2, the exponent ℓ is 100, and Δt is the length of a time step in minutes (normally 1). Thus, a cell becomes increasingly likely to divide as its area approaches A_c. Although this probabilty has some dependence on the length of the time step Δt, it is much more strongly dependent on the size of the cell because of the large value of ℓ. This means that the areas of the largest and smallest cells differ by roughly a factor of 2, a narrower range than the approximate factor of 3 measured experimentally [1].

To find the angle at which a cell divides, we find the cell's single closest neighbor (in terms of distance between centers) and its angular position (i.e., the angle associated with a vector drawn from the dividing cell's center to the center of the nearest neighboring cell), then set the division plane at that angle. Thus, cells with an elongated shape will tend not to divide lengthwise, consistent with observed results [3]. This method of selecting the division angle has the added effect of minimizing overlap between the daughter cells and existing neighbors, allowing the new cells to begin closer to mechanical equilibrium. However, we have also tried different criteria for determining the division angle, including selecting it at random and selecting it based on the angle of Dachs localization, with minimal effect on the overall results.

When the cell divides, the neighbor lists of the daughter cells are rebuilt. The radii of the daughter cells are chosen such that each one has half the area that its progenitor did when it divided. These radii are then each adjusted by a small amount (randomly chosen between -1% and +1%) so that the areas are slightly different. This prevents entire clones of cells from dividing at nearly the same time.

3 Ft-Ds-Fj kinetics

In this section we discuss the expression of Fat (Ft), Dachsous (Ds), and Four-jointed (Fj), as well as the formation and dissolution of Ft-Ds heterodimers between adjacent cells. The

asymmetry of the distribution of these bonds around a cell contributes to the cell's growth rate and polarity.

3.1 Initial conditions and morphogen dynamics

In the model, Dpp and Wg are conflated into a single quantity that we call the morphogen. When the simulation starts, each cell is given values for its starting amounts of morphogen, Ft, Ds, and Fj. The morphogen concentration decays exponentially with radial distance r from the center of the wing disc; it is greatest in the center of the disc. The most important feature of the morphogen profile in this model is that the amount of morphogen signaling in any given cell increases over time. We see similar results regardless of whether the decay length remains constant or scales with the size of the disc, as long as each cell experiences an overall increase in morphogen throughout the run. The amplitude of the profile increases over time, and the decay length scales with the overall radius of the disc. The morphogen concentration in the disc as a function of time and position is given by

$$[M] = C_0 e^{\left(\frac{t}{t_0} - \frac{Ar}{R}\right)} \tag{S9}$$

Here, $[M]$ is the morphogen concentration, and $C_0 = 1.5$ is the starting morphogen amplitude. t is the current time in minutes (starting with 0) and $t_0 = 1200$ minutes represents the length of time over which the morphogen concentration increases by a factor of e at a given point [4]. r is the distance from the disc's center in μm, R is the radius of the disc in μm, and A is a constant, set to 4. This means that the decay length of the morphogen (i.e., the distance over which the morphogen concentration decreases by a factor of e) is 1/4 the radius of the simulated disc, and scales as the disc grows, as experimental results suggest [4].

Each cell is also given a starting pool of 1×10^3 Ft. The starting amounts of Ds and Fj in a given cell are based on the cell's amount of morphogen:

$$\mathrm{Ds}_{initial,i} = A_{Ds,initial}\left(\mathrm{Ds}_{min} + \mathrm{Ds}_{range}\left(\frac{1}{1+\left(\frac{[M]_i}{[M]_c}\right)^m}\right)\right) \tag{S10}$$

Here, $\mathrm{Ds}_{initial,i}$ is the number of Ds proteins that cell i initially has. $A_{Ds,initial}$ is a constant coefficient of 1×10^3, Ds_{min} is 0.2, and Ds_{range} is 0.8. $[M]_i$ is the cell's morphogen concentration as calculated in Equation (S9) using the cell's radial position for r, $[M]_c$ is a constant critical morphogen concentration of 0.5, and the exponent m is 10. We choose this function for its sigmoidal dependence on $[M]_i$, causing cells with higher concentrations of morphogen (those near the disc's center) to have a low level of Ds and cells with less morphogen (those near the disc's edge) to have a high level of Ds, with a relatively narrow transition region between the two. The function also has a constant offset Ds_{min}, allowing cells near the center of the disc to have a small but nonzero amount of Ds.

According to experimental data, Fj is present in a roughly linear profile with a high concentration at the center of the disc that decreases with radial position [5]. Our model,

then, uses a linear Fj concentration profile:

$$[\mathrm{Fj}]_{initial,i} = [\mathrm{Fj}]_{min} + \mathrm{Fj}_{slope}(r_{\max} - r_i) \tag{S11}$$

where $[\mathrm{Fj}]_{initial,i}$ is the initial Fj concentration in cell i. (Unlike with Ft and Ds, where we track individual proteins, [Fj] in our model is a continuous quantity, like morphogen.) Here, $[\mathrm{Fj}]_{min}$ is set to 0.1 and Fj_{slope} is set to 0.025 $\mu\mathrm{m}^{-1}$. r_i is the radial position of the cell, and $r_{\max}$ is the radial position of the edge of the disc, both measured from the center. Thus, the Fj concentration at a given radial position increases over time as the disc grows. In the case of discs or clones that express no Fj, $[\mathrm{Fj}]_{initial,i}$ is directly set to 0. Discs or clones that overexpress Fj have $[\mathrm{Fj}]_{initial,i}$ set to 10, considerably higher than the typical Fj concentration during a normal run.

The overall result for a wild-type disc is an exponential morphogen profile, a sigmoid profile of Ds, and a linear profile of Fj as shown in Fig 2 of the main text. Fj is expressed at high levels in the center of the disc and decreases with radial distance. The Ds profile is fairly flat in the wing pouch and the periphery, with a steep transition region at the edge of the wing pouch.

Ft and Ds can be phosphorylated by Fj, affecting their binding affinities. Specifically, phosphorylation by Fj makes Ft more likely and Ds less likely to bind [5–7]. In our simulation, Ft and Ds are divided into phosphorylated and unphosphorylated pools. The fraction of starting phosphorylated Ft in cell i is:

$$\frac{\text{initial phosphorylated Ft}, i}{\text{initial total Ft}, i} = C_1(1 - P_{\text{base},i}) + P_{\text{offset}} \tag{S12}$$

Similarly, the fraction of starting phosphorylated Ds in cell i is:

$$\frac{\text{initial phosphorylated Ds}, i}{\text{initial total Ds}, i} = C_2(1 - P_{\text{base},i}) + P_{\text{offset}} \tag{S13}$$

Here, C_1 and C_2 are both set to 0.8. (This value is chosen simply because it is less than but close to 1, allowing the range of phosphorylation fractions to be fairly large.) P_{offset}, set to 0.1, represents the fraction of phosphorylated Ft or Ds in the absence of Fj. Thus, the phosphorylation fractions range from 0.1 to 0.9 for both Ft and Ds. The base probability $P_{\text{base},i}$ is lower at high Fj concentrations:

$$P_{\text{base},i} = \frac{1}{1 + \left(\frac{[\mathrm{Fj}]_i}{[\mathrm{Fj}]_c}\right)^n} \tag{S14}$$

Here, $[\mathrm{Fj}]_i$ is the amount of Fj in cell i, $[\mathrm{Fj}]_c$ is a constant critical quantity of Fj (set to 0.3), and the exponent $n = 2$ determines the steepness of the dependence.

Once amounts of Fj and phosphorylated and unphosphorylated Ft and Ds are established for each cell, the Ft-Ds binding algorithm is run once, so each cell begins the simulation proper with a set of Ft-Ds bonds. As the simulation continues, the number and distribution of these bonds can change.

3.2 Updating Ft, Ds, and Fj

During each time step, new Fj and free Ft and Ds can be expressed and degrade, Ft-Ds bonds can form and dissolve, and free Ft and Ds can become unphosphorylated. The chance of a new Ft-Ds bond forming depends on the phosphorylation states of the individual Ft and Ds proteins. The Fj and Ds expression rates remain dependent on a cell's position within the disc.

At each time step, a cell's concentration of morphogen is updated based on its radial position within the disc and the current time using Equation (S9). All cells have the same constant production and degradation rates of free Ft, since Ft is uniformly expressed in the wing disc compared to Ds and Fj [8, 9]. Ft can also bind and unbind with Ds. The rate of change of free Ft in cell i is given by:

$$\frac{d(\text{free Ft})_i}{dt} = R_{Ft} - k_{d,Ft}(\text{free Ft})_i - \frac{d(\text{bound Ft})_i}{dt} \tag{S15}$$

R_{Ft} is the rate of Ft expression, 20 molecules per minute; the degradation rate $k_{d,Ft}$ is 0.08 min^{-1}. The process of Ft-Ds binding and unbinding determines the rate of change of bound Ft in each cell, and is described in the next subsection.

The production rate of free Ds is based on each cell's amount of morphogen such that Ds expression is highest near the edge of the disc. Ds can bind and unbind, and free Ds can degrade:

$$\frac{d(\text{free Ds})_i}{dt} = R_{Ds,max}\left(\text{Ds}_{min} + \text{Ds}_{range}\left(\frac{1}{1+\left(\frac{[M]_i}{[M]_c}\right)^m}\right)\right) - k_{d,Ds}(\text{free Ds})_i - \frac{d(\text{bound Ds})_i}{dt} \tag{S16}$$

$R_{Ds,max}$ is the maximum rate of Ds production; it is set to 20 molecules per minute. Ds_{min}, Ds_{range}, $[M]_i$, $[M]_c$, and m are as in Equation (S10). Thus, the expression of new Ds largely parallels the starting concentration profile; cells near the center of the disc will have a high amount of morphogen and their rate of Ds production will be low, while cells closer to the edge will have less morphogen and express Ds faster. Free Ds also degrades at a rate $k_{d,Ds}$ equal to 0.08 min^{-1}, like Ft. As with Ft, the rate of change of bound Ds in a given cell is determined by the formation and dissolution of Ft-Ds bonds, described in the next subsection.

The production rate of Fj in each cell varies linearly with radial position:

$$\frac{d[\text{Fj}]_i}{dt} = R_{Fj,max}\left([\text{Fj}]_{min} + \text{Fj}_{slope}(r_{\max} - r_i)\right) - k_{d,Fj}[\text{Fj}]_i \tag{S17}$$

The constants $[\text{Fj}]_{min}$ and Fj_{slope} are as in Equation (S11), so again, the rate of new Fj production parallels the initial Fj profile. r_i is the radial position of cell i and $r_{\max}$ is the overall radius of the disc. $R_{Fj,max}$ is the maximum rate of Fj production, set to 0.05 min^{-1}. The degradation rate $k_{d,Fj}$ is 0.05 min^{-1}. Unlike the number of Ft and Ds on a cell, the amount of Fj is not an integer in our model; it is normally of order 1. As in the initial

conditions, newly expressed free Ft and Ds are immediately divided into phosphorylated and unphosphorylated pools using the same ratios as in Equations (S12) and (S13), with "new phosphorylated Ft/Ds, i ÷ total new Ft/Ds, i" in place of "initial phosphorylated Ft/Ds, i ÷ initial total Ft/Ds, i". The same degradation rate applies to both phosphorylated and unphosphorylated free Ft and Ds each time step. Ft and Ds proteins that are newly freed (i.e., from a bond that was just dissolved) retain their phosphorylation state and move to their respective free pools. As a separate mechanism, phosphorylated Ft and Ds on a cell can also become unphosphorylated:

$$\frac{d(\text{phosphorylated Ft})_i}{dt} = -D(\text{phosphorylated Ft})_i \tag{S18}$$

Note that this only describes the dephosphorylation of existing Ft, and is handled separately from Ft expression and degradation. Here, D is a dephosphorylation rate for phosphorylated Ft, set to 1.28×10^{-3} min^{-1}. The rate of change of unbindable Ft is simply the negative of this equation.

Ds behaves similarly:

$$\frac{d(\text{phosphorylated Ds})_i}{dt} = -D(\text{phosphorylated Ds})_i \tag{S19}$$

D, the dephosphorylation rate, is the same as in Equation (S18). Again, this equation only applies to existing Ds and does not include Ds expression and degradation. The rate of change of unphosphorylated Ds is the negative of this equation.

3.3 Binding algorithm

We now describe the algorithm by which Ft, Ds and Fj interact to form bonds. The following process is run for all cells during each time step:

- Unbind a certain fraction of all bound Ft and bound Ds on all cells, specifically 0.08 per minute. If either number (0.08 times the number of bound Ft or Ds) is less than 1 for a given cell, use it instead as the probability of undoing a single bond on that cell. The phosphorylation states of the now-unbound Ft and Ds are retained.

- Degrade a certain fraction of all free Ft and Ds on all cells, specifically $k_{d,Ft} = k_{d,Ds} = 0.08$ min^{-1}. If this number (0.08 times the number of free Ft or Ds) is less than 1 for a given cell, use it instead as the probability of degrading one Ft or Ds on that cell. This degradation is independent of phosphorylation state.

- Handle dephosphorylation of free Ft and Ds on all cells based on Equations (S18) and (S19) above. If less than 1 free Ft or Ds is to be dephosphorylated in a given cell, use this number instead as the probability of dephosphorylating a free Ft or Ds on that cell.

- Degrade a certain fraction, specifically $k_{d,Fj} = 0.05$ min^{-1}, of the Fj in each cell.

- Update each cell's morphogen concentration based on the cell's position within the disc and the current time, using Equation (S9).
- Produce new (free) Ft and Ds, as well as new Fj, in each cell. The Ft production rate is constant, the Fj production rate depends on radial position, and the Ds production rate depends on the morphogen level, as shown in Equations (S16) and (S17).
- Separate the newly expressed free Ft and Ds into phosphorylated and unphosphorylated pools in each cell. A fraction equal to $(1 - P_{\text{base},i}) + P_{\text{offset}}$ of the new free Ft and Ds becomes phosphorylated. $P_{\text{base},i}$ is given in Equation (S14). If the number of Ft or Ds entering either pool in a given cell is less than 1, use this number instead as the probability of moving a single Ft or Ds into this pool for that cell.
- Now, iterate the following for all pairs ij of adjacent cells (as determined by the Delaunay triangulation):
  - If the two cells are too far apart, do not count them as adjacent for purposes of Ft/Ds binding. (In certain cases, the neighbor-list algorithm can make cells near the disc's edge neighbors even when they are not touching.) In particular, if the edges of the cells are separated by a gap of at least 0.8 μm, skip this pair of cells and move on.
  - Note the current number of free Ft and Ds of all phosphorylation states on these two cells.
  - Determine how much of the available Ft and Ds on cell i to allot to cell j and how much of the available Ds and Ft on cell j to allot to cell i. Each cell divides its number of Ft and Ds by its number of neighbors to determine the number to allot to each neighbor. Thus, a cell will not risk binding more Ft or Ds than it has. When this quantity is calculated for the Ft on cell i, a complementary quantity is calculated for the Ds on cell j and vice versa.
  - Form new Ft-Ds heterodimers. Note that bonds of different types (Ft-Ds, FtP-Ds, Ft-DsP, and FtP-DsP) can be more or less likely to form, depending on the phosphorylation states of the Ft and Ds proteins involved. For example, the number of new FtP-Ds bonds formed is the lesser of the phosphorylated Ft on cell i allotted to cell j and the unphosphorylated Ds on cell j allotted to cell i, multiplied by a weight parameter specific to the bond type (FtP-Ds). New Ds-FtP bonds are formed from the unphosphorylated Ds on cell i and the phosphorylated Ft on cell j using a similar method, and so on.
  - The newly bound Ft and Ds are then moved from the free to the bound pool on their corresponding cells. The simulation continues to track their phosphorylation states, should they become unbound later.

The weight parameters for the four bond types are:

- 0.7 for FtP-Ds (phosphorylated Ft with unphosphorylated Ds)
- 0.2 for FtP-DsP
- 0.05 for Ft-Ds
- 0.05 for Ft-DsP

Since this process is run for every cell at every time step, cells quickly bind as many of their Ft and Ds as possible. Note that the formation of new bonds at each time step is independent of the arrangement of existing bonds. In other words, if cell i already has many Ft bound to the Ds on cell j, it still does not show any special preference for cell j when binding additional Ft. Only the number of available Ft and Ds on each cell, their phosphorylation states, and the neighbor list, determine where new bonds are formed.

4 Nondeterministic aspects

Only a few minor aspects of the model are nondeterministic. The size at which a cell divides is not fixed; a cell's probability of dividing in a given time step is given by a Hill function that rises very steeply as the cell reaches a certain size. As mentioned earlier, the relative areas of the two daughter cells are distributed slightly unevenly to prevent their possible simultaneous division later. Ft-Ds kinetics (binding, unbinding, dephosphorylation, expression, and degradation) are also handled stochastically when the number of proteins expected to change in a single time step is less than one. These aspects, when averaged over many cells and many time steps, have little effect on the overall growth rate of the simulated disc, so the eventual size of the disc typically varies by less than 1% between runs using the same set of parameters.

5 Growth rate and adaptation

Here, we describe in detail the model for the growth rate of a cell, as well as the integral feedback mechanism that allows cells in our model to adapt to consistent signals.

The growth rate of a cell in our model depends on three factors: the distribution of its Ft-Ds bonds, its amount of morphogen signaling, and its number of leftover free Ft and Ds. The more asymmetrically distributed a cell's Ft-Ds bonds, the faster it grows. This asymmetry arises as a result of the spatially graded expression of Dachsous and Four-jointed. Experimentally, discs that express Ds or Fj uniformly are observed to undergrow, while discs with a single clone that overexpresses Ds or Fj show growth around the edge of the clone [10]. Thus, a cell whose bound Ft is distributed very nonuniformly among its neighbors should grow faster than a cell whose bonds are distributed evenly. Also, discs mutant for both Ft and Ds overgrow faster than discs mutant for only one or the other [11], suggesting that the number of free Ft and Ds should also affect the growth rate to some degree. In experiments,

the disc also appears to adapt to consistently strong signaling both in and near a clone of cells with elevated Dpp signaling, with the result that the response dies away after multiple days [12]. This result suggests that the cells in the disc may use some form of integral feedback [13] to adapt to unchanging signaling, both from the morphogen and from Ft-Ds bond asymmetry. Here, then, is a model for cell growth that reflects all of these results.

At every time step, each cell grows by a certain ratio; that is, its new radius $r_{n+1,i}$ becomes a certain multiple of its radius at the previous time step $r_{n,i}$:

$$\frac{r_{n+1,i}}{r_{n,i}} = 1 + G_0 \frac{(1 + C_M x_{M,i})(1 + C_{Ft} x_{Ft,i})(1 + C_{Ds} x_{Ds,i})}{(1 + U_i)} \tag{S20}$$

G_0 is a constant base growth rate of 4.3×10^{-4} min^{-1}. C_M, C_{Ft}, and C_{Ds} are constant coefficients with units of time representing the relative strengths of the responses to morphogen signaling and to asymmetric bound Ft and Ds; they are equal to 40 min, 3000 min, and 3000 min, respectively. $x_{M,i}$, $x_{Ft,i}$, and $x_{Ds,i}$ represent the disc's responses to morphogen signaling and asymmetry in Ft and Ds bonds, respectively, after applying integral feedback to represent adaptation (explained below). U_i is proportional to the number of leftover free Ft and Ds on cell i; it is 3×10^{-5} times their sum.

5.1 Integral feedback

In experiments, cells in the disc exhibit growth that is autonomously dependent on their amount of Dpp [12,14]. Thus, a cell's growth rate in our model should be partially dependent on the morphogen concentration in that cell. Similarly, the growth rate should depend on the degree of asymmetry in a cell's Ft-Ds bonds. However, the autonomously elevated growth rate within clones of cells with artificially high Dpp signaling wanes over time, as does the nonautonomous effect near steep spatial gradients of Fj or Ds [10,12]. Our model of growth, then, also allows for adaptation to a constant signal via integral feedback [13]. To do this, we consider a cell's "response" to morphogen signaling as separate from its instantaneous morphogen concentration. This response depends both on the local morphogen concentration and on the time integral of the response from the start of the simulation to the current time step.

We use $x_{M,i}(t+1)$ to represent the response of cell i to morphogen signaling at time step $t+1$; it is given by

$$x_{M,i}(t+1) = G_M \left([M]_i(t+1) - \int_0^t x_{M,i}(\tau)\ d\tau \right) \tag{S21}$$

where G_M is a constant parameter and $[M]_i(t+1)$ is the morphogen concentration in cell i given by Equation (S9) at time step $t+1$. Note that if the morphogen concentration in cell i is constant, then $x_{M,i}$ will decrease such that $\int x_{M,i}(\tau)\ d\tau$ will approach the same constant, and the instantaneous value of $x_{M,i}$ will approach zero. The coefficient G_M, representing the speed with which the growth rate adapts to consistent morphogen signaling, is 2.8×10^{-2}

min^{-1}. We choose this value so that the adaptation has a short characteristic time scale (less than an hour), meaning the growth rate is roughly proportional to the rate of change of morphogen concentration, as experimental evidence suggests [4].

Similarly, each cell's growth rate adapts to the degree of asymmetry in its bound Ft:

$$\text{Ft asymmetry}_i = 1 - S_{F,i} \tag{S22}$$

$$x_{Ft,i}(t+1) = G_{Ft}\left(\text{Ft asymmetry}_i(t+1) - \int_0^t x_{Ft,i}(\tau)\ d\tau\right) \tag{S23}$$

The same mechanism applies to the asymmetry of bound Ds:

$$\text{Ds asymmetry}_i = 1 - S_{D,i} \tag{S24}$$

$$x_{Ds,i}(t+1) = G_{Ds}\left(\text{Ds asymmetry}_i(t+1) - \int_0^t x_{Ds,i}(\tau)\ d\tau\right) \tag{S25}$$

The coefficients G_{Ft} and G_{Ds}, representing the speed with which the disc adapts to consistent asymmetry signaling, are both equal to 6×10^{-4} min^{-1}. Experimentally, the nonautonomously elevated growth around clones of cells with activated Tkv decreases with a characteristic time scale on the order of tens of hours [12], so we choose smaller values for G_{Ft} and G_{Ds} than for G_M. $S_{F,i}$ and $S_{D,i}$ are defined in the next subsection where we describe our method for quantifying how symmetrically a cell's protocadherins are distributed.

5.2 The minimum fraction metric for symmetry

The quantities $S_{F,i}$ and $S_{D,i}$ are proportional to a cell's minimum fractions of bound Ft and Ds. To find the minimum fraction of bound Ft for $S_{F,i}$, for example, the cell checks each of its neighbors to find the minimum number of Ft proteins bound to any neighbor, then divides this amount by its total number of bound Ft. We call the result the minimum fraction. (In the event that the cell has no bound Ft whatsoever, it simply sets this fraction to zero to avoid dividing by zero.) It then multiplies this fraction by its number of neighbors to compensate for the fact that cells have different numbers of neighbors. For example, in Figure S3, the central cell has a total of 12 Ds bonds with its six neighbors. The smallest number of bonds it has with any single neighbor is the one Ds bond with the bottom cell. The minimum fraction is then 1/12. If we then multiply by 6 (the number of neighbors), we obtain 6/12 or 1/2, a quantity we refer to as the adjusted minimum fraction. Thus, a cell that divides its bound Ft or Ds evenly among all its neighbors will have this quantity equal to 1, and a cell with a more asymmetric distribution of bonds will have a smaller number. If a cell has no bonds with at least one of its neighbors, its adjusted minimum fraction will be zero.

The adjusted minimum fractions are measures for bond symmetry and are given by:

$$S_{F,i} = \text{min. bound Ft fraction}_i \times \#\text{neighbors of cell } i \tag{S26}$$

$$S_{D,i} = \text{min. bound Ds fraction}_i \times \#\ \text{neighbors of cell } i \tag{S27}$$

Thus, from Equation (S20), cells with uniformly distributed bound Ft and Ds (i.e., higher minimum fractions) grow more slowly than cells whose Ft-Ds bonds are more asymmetrically distributed (i.e., lower minimum fractions). In the case of a disc that is mutant for either Ft or Ds (or both), both $S_{F,i}$ and $S_{D,i}$ will be set to zero for all cells, since no cell has any bound protocadherins. If this case were not treated specially, the minimum fraction would be undefined, since its denominator is the total number of bound Ft or Ds on a cell. Note that this special case only applies if a cell has no bonds with one or more of its neighbors. In our simulations, this situation only occurs in discs that are completely missing Ft and/or Ds, or where Ft or Ds lack the ability to bind. In wild-type discs and most other cases, every cell will have enough Ft and Ds to form at least one bond with each of its neighbors. Under normal conditions, cells in the model will always have at least one bond with each of their neighbors. (Experimentally, both Ft and Ds are detected at all cell-cell interfaces in a wild-type disc [15].)

We note that the asymmetry of Ft-Ds bonds around a cell may be defined in a number of ways. However, in order to relate bond asymmetry to Dachs localization on the side with the least bound Ft [10, 16, 17], we chose to measure the asymmetry of the bond distribution using only the neighbor with the fewest bonds. This was primarily so that a cell with no Ft-Ds bonds at all (e.g., a *ft-* or *ds-* cell) would exhibit the strong overgrowth seen experimentally [11]. Cells with no bonds in our model would be treated as having a minimum fraction of zero, and thus very high asymmetry.

One could also argue that cells lacking Ft-Ds bonds have very low asymmetry, since they have the same number of bonds (i.e., zero) with each of their neighbors. However, other factors also make our minimum-fraction mechanism a plausible one. In particular, the myosin Dachs is observed to localize to the side of each cell with the least bound Ft [10], suggesting that Ft signaling may involve mechanotransduction. Ft signaling is also known to affect microtubule orientation and mitotic angle [18], indicating a link with cytoskeletal organization. In addition, Yorkie, which is downstream of Dachs in the Ft pathway, is homologous to YAP (Yes-associated protein). YAP is a transcriptional coactivator associated with cell-ECM interactions and mechanotransduction [19]. We speculate that the Ft pathway may be involved in sensing the mechanical stresses that a cell experiences from its neighbors, and in how cells proliferate in response to these stresses. However, our model does not address these effects directly. Rather, it is primarily the asymmetry in the distribution of Ft-Ds bonds around a cell that affects growth. We speculate that this asymmetry may be sensed via mechanical tension in the cytoskeleton from the Ft-Ds bonds. This is a separate mechanism from the bulk mechanical forces in our model that only prevent adjacent cells from overlapping or separating. In our model, these mechanical forces do not directly affect a cell's growth rate.

When a cell divides, the mother and daughter cells will initially have no Ft-Ds bonds between them, since their interface is newly formed. Similarly, cells may rearrange as a result of mechanical forces from their neighbors, forming new interfaces. However, pairs of adjacent cells that formed within the last 100 minutes are ignored for the purpose of calculating bond

asymmetry in the model. Otherwise, any pair of cells resulting from a recent division would have very small minimum fractions because they have very few bonds with one another, and thus they would have a very high growth rate due to bond asymmetry. The daughter cell inherits the mother cell's values of Ft and Ds asymmetry until this grace period has passed.

We also include a penalty to a cell's growth rate for having leftover Ft and Ds. In experiments, discs that are mutant for both Ft and Ds are observed to grow faster than either single mutant [11], even though both single mutants should be completely lacking in Ft-Ds bonds. Since the only difference between the single and double mutants is that the single mutant contains a large number of free Ft or Ds, we speculate that free Ft and Ds have a tendency to slow growth. Similarly, cells in a Ft mutant disc proliferate faster in the center (where little Ds is present) than at the edge (where the most Ds is present) [20], suggesting that excess unbound Ds may be associated with slower growth.

6 Dachs localization

Here, we describe our model for the magnitude and direction of Dachs localization within each cell. We can use Dachs localization as an indicator of Ft-Ds bond asymmetry, allowing us to see which cell-cell interfaces form bonds and what polarity those bonds have. We implement a model of Dachs localization as driven by Ft bond distribution asymmetry. Each cell has a vector $\overrightarrow{\mathrm{Dachs}_i}$ representing its Dachs localization whose magnitude varies between 0 (isotropically distributed) and 1 (fully localized to one side), and whose direction gradually changes to point towards the side of the cell with the least bound Ft:

$$\frac{d}{dt}(\overrightarrow{\mathrm{Dachs}_i}) = (1 - S_{F,i})\hat{p}_i - \overrightarrow{\mathrm{Dachs}_i} \tag{S28}$$

Here $S_{F,i}$ is the cell's adjusted minimum fraction of bound Ft (i.e., adjusted for the number of neighbors) as in Equation (S26). It varies from 1 (evenly distributed bonds) to 0 (at least one neighbor with no bonds). The quantity $(1 - S_{F,i})$ is a measure of bond asymmetry, causing Dachs to become more strongly polarized the more asymmetrically the bonds are distributed. The quantity $\hat{p}_i$ is simply a unit vector pointing toward the neighbor with which the cell has the least bound Ft. Thus, Dachs moves toward the side of a cell with the least bound Ft. Note that Dachs localization matches the magnitude and direction of Ft asymmetry at every time step. Because there is a "grace period" before newly formed neighbors can contribute to the minimum fraction of bound Ft, Dachs polarization is not instantaneously affected by changes in a cell's set of neighbors caused by cell division.

7 Building the neighbor list

In this section, we explain how the neighbor list is built at the beginning of a run, and how it is maintained as the cells grow and divide. When the simulation is launched, the full neighbor list must be determined. The neighbor list does not need to be entirely rebuilt

at every time step, since the cells in the simulation do not rearrange substantially except to accommodate their neighbors' growth and proliferation. (This appears to be the case experimentally as well, as suggested by the distribution of the neighbor counts of cells in a tissue [1, 21]). However, cells do move with respect to one another fast enough that the neighbor list must be rebuilt occasionally. The main loop of the program keeps track of how fast its cells are moving as well as the time since the neighbor list was last rebuilt. Whenever enough time steps have passed that a cell can have moved a distance equal to 0.2 times the average cell radius at that time step, or if 10 time steps have passed since the list was rebuilt, then the entire neighbor list will be rebuilt.

Furthermore, a cell's set of neighbors can change when it or an adjacent cell divides. So a cell that divides will need to partition its neighbors between the two daughter cells. Thus, the simulation is also written to handle rebuilding the neighbor list for a small subset of the disc's cells (i.e., those adjacent to a cell that has just divided), and then reintegrating the results into the main neighbor list. This improves the running time by avoiding rebuilding the full list every time a cell divides, which can happen many times per time step when the disc is large.

7.1 Delaunay triangulation

The neighbor list is built by a separate routine that constructs a Delaunay triangulation given a set of points in two dimensions. A Delaunay triangulation is a mesh of triangles drawn on a set of vertices such that no triangle's circumscribed circle contains any other vertex besides the three used to construct it (Figure S4). Here, each point represents the center of a cell, and only pairs of points (i.e., pairs of cells) connected by an edge are considered neighbors.

When the neighbor list must be rebuilt, the program steps through the list of cells and stores the coordinates and label of each one together. The resulting data structure is then passed to a separate function, which builds a triangulation recursively [22]. The algorithm works as follows:

- If there is only one vertex, no edges need to be constructed. If there are exactly two vertices, draw an edge between them. If there are three vertices, draw three edges to form a triangle. This is the base case.

- If there are more than three vertices, separate the vertices evenly into two sets by x-coordinate, leaving a left and a right half. (If there is an odd number of vertices, we allow the right half to have one more vertex than the left half.) Recursively, i.e., using this algorithm, build a triangulation from each half.

- To merge the left and right halves, first draw the bottom-most edge connecting the halves that lies on the overall convex hull of all the points. (The convex hull is the convex polygon made by connecting all of the outermost points; it contains all of the points.) In other words, all points on both halves of the triangulation should lie on only one side of this edge. We call this the "base edge" and its vertices A and B (Figure S5, panel 1).

- The next edge connecting the two triangulations will connect one vertex of the base edge with a vertex that is adjacent to the base edge's other vertex. In other words, if the vertices of the current base edge are labeled A and B, the next edge will either connect A with a neighbor of B, or connect B with a neighbor of A. This edge must not intersect any existing edges. In addition, the triangle formed by A, B, and this chosen vertex must contain no other vertices within its circumscribed circle (i.e., it must satisfy the main Delaunay condition). There should be no more than one vertex that fulfills all these conditions; there may be none.

- Find candidate vertices for this next edge. Check each neighbor of A and B, in ascending order of angle made with the base edge. Exclude any neighbors that lie below the base edge. If the triangle formed by the base edge and the current candidate contains the next candidate within its circumscribed circle, delete the edge between the candidate and its base vertex, and then move on to the next candidate (Figure S5, panel 2). Otherwise, choose this candidate. Do this for both the left and right sides. It is possible for a side to have no candidates.

- Choose the next step depending on whether two, one, or zero candidates have been selected:

  - If each side chooses a candidate, say C and E, look at the triangles formed by each candidate and the base edge. If the circumscribed circle of triangle ABC does not contain E, choose C; otherwise, choose E (Figure S5, panel 3). Draw an edge between the chosen candidate and the vertex of the base edge lying on the other side of the triangulation. This edge becomes the new base edge and the process is repeated (Figure S5, panel 4).
  - If only one side chooses a candidate, an edge is drawn from it to the vertex of the base edge that lies on the other side of the triangulation. This edge becomes the new base edge and the process is repeated.
  - If no candidate is found from either side, the two triangulations are now fully merged (Figure S5, panel 5).

- The process continues recursively, building and merging triangulations. Once all merges are finished, the resulting triangulation represents the complete neighbor list.

7.2 Building a subset of the list

Whenever a cell divides, the two daughter cells will likely have different sets of neighbors. To update the neighbor list efficiently, the main program sends the locations of the daughter cells as well as those of all the mother cell's original neighbors to the triangulation subroutine. The resulting neighbors are then integrated into the main neighbor list. Because cell divisions happen frequently, building only a small subset of the neighbor list, and then splicing this

subset into the main list greatly lowers the running time compared to rebuilding the full list every time a cell divides.

A dividing cell will generally not retain the exact set of neighbors that it had before division. The mother cell's neighbors are either shared between the two daughter cells or are retained by only one of the two daughter cells (Figure S6). Because each pair of neighboring cells (or "neighbor pair") is associated with a number of Ft-Ds bonds, these bonds must be accounted for each time a cell divides. When a cell divides, it becomes one of the two daughter cells (retaining its old numerical index) and a new daughter cell with a new index is created. Thus, several scenarios are possible for each neighbor of a dividing cell:

- If a neighbor stays with the original mother cell and is not shared with the newly created daughter, no action is necessary. Its bound Ft and Ds are retained.
- If a neighbor of the mother cell is now a neighbor of both daughters, its bound Ft and Ds are split evenly between the two. In other words, half the Ft-Ds bonds that this cell had with the mother cell are transferred to the new daughter cell. The other half are retained by the mother cell. The phosphorylation states of the constituent Ft and Ds are still tracked.
- If a neighbor of the mother cell is now only a neighbor of the newly created daughter, the Ft-Ds bonds between the mother and that neighbor are all transferred to the daughter.
- If a neighbor pair is formed between cells that are neither the mother nor daughter (and this pair is not already part of the main neighbor list), it is expunged, as these two cells are not actually adjacent. No action involving Ft and Ds is necessary, since the neighbor list has not changed.

The mother and daughter cells will initially have no Ft-Ds bonds between them, since their interface is newly formed. However, neighbor pairs formed within the last 100 minutes are ignored for the purpose of calculating bond asymmetry. Otherwise, any pair of cells resulting from a recent division would have very small minimum fractions because they have very few bonds with one another, and thus they would have a very high growth rate due to bond asymmetry. The daughter cell inherits the mother cell's values of Ft and Ds asymmetry until this grace period has passed.

8 Additional results

In this section, we present results beyond those presented in the main text, including patterns of cell growth when the Ds expression front is stationary, as well as patterns of growth and bond asymmetry near Fj-overexpressing and Ds-overexpressing clones.

8.1 Overall growth of the disc under various conditions

A number of experimental results on disc growth are given in terms of the eventual size of the wing disc. We show a summary of the results of our model in Figure S7, where the number of cells in the disc is given for various conditions. In some runs, we remove Ft, Ds, both Ft and Ds, or Fj. In other runs, we examine discs that express Fj, Ds, or both Fj and Ds at uniformly high levels. We simulate a disc that underexpresses Ft without lacking it entirely. We also simulate discs in which Ft is lacking its intra- or extracellular domain. Finally, we examine discs that either lack the morphogen or have uniformly high morphogen signaling. We compare each of these cases to the growth of a wild-type case as a control. All other parameters are fixed between runs. The results of our model are qualitatively consistent with a variety of experiments, as we describe in detail below.

The dramatic overgrowth of mutant *ft-* discs is widely observed experimentally [11,23–25]. Discs mutant for *ds* overgrow to a lesser degree [11, 26, 27], although the size of adult *ds-* wings depends on the exact allele used. Furthermore, *ft- ds-* double mutants overgrow more than either single mutant [11].

In our model, discs that are mutant for Ft and/or Ds overgrow substantially compared to wild-type discs (Figure S7, *"ft-"*, *"ds-"*, and *"ft- ds-"*). When a cell lacks Ft-Ds bonds with at least one of its neighbors, its bond distribution is considered to be completely asymmetric, and bond asymmetry is associated with faster growth as given in Equation (2) in the main text. However, discs lacking either Ft or Ds will have their growth rate somewhat penalized by the substantial amount of free, or unbound, Ds or Ft (respectively) remaining. In particular, discs mutant for Ft grow slightly faster than discs mutant for Ds in our model simply because a disc normally contains more Ft than Ds. In other words, a *ds-* disc will have more free Ft than a *ft-* disc will have free Ds, and free Ft and Ds hinder growth to the same extent. Discs that are mutant for both Ft and Ds will grow fastest, since they have no free Ft or Ds.

Experimentally, discs that uniformly express Ds or Fj tend to undergrow compared to wild-type discs, and discs expressing both Ds and Fj uniformly undergrow even more [10,28]. This is consistent with our model. The main drivers of Ft-Ds bond asymmetry in our model are the Ds and Fj gradients as well as the movement of the Ds front, which lead to a spatially nonuniform availability of Ds as well as nonuniform binding probabilities for Ft and Ds. (Recall that Fj affects the binding probabilities of Ft and Ds [5–7].) Because the model's growth rate is heavily dependent on Ft-Ds bond distribution asymmetry, making the Ds or Fj concentrations spatially uniform will lead to slower growth (Figure S7, "uniform Fj" and "uniform Ds"). If both Fj and Ds are expressed uniformly, the growth rate will be slower still, because both the availability and the binding probabilities of Ft and Ds will be spatially uniform (Figure S7, "uniform Fj and Ds").

Flies mutant for Fj tend to have smaller wings than wild-type flies [28–31]. In our model discs that lack Fj grow more slowly than wild-type discs, since the Ft and Ds binding affinities do not vary across the disc, leading to more uniform bond distributions on each cell (Figure S7, "fj-").

When we simulate different forms of Ft and their effects on the growth rate, we find

results that are consistent with experiment. For example, discs that express FtΔICD, a form of Ft missing its intracellular domain (ICD), overgrow more than *ft-* discs and slightly less than *ft- ds-* double mutant discs [11]. In our model, this form of Ft still binds to Ds but is not counted by its cell as either bound or free. Similarly, the Ds bound to this form of Ft is also not recognized by its cell because we assume that the Ft-Ds bonds need to be tethered to the cytoskeleton on both ends of the bond for mechanotransduction to occur. The result is a disc that grows very quickly, as each cell recognizes no bound Ft or Ds, experiences no growth penalty for having free Ft, and has only a small penalty for its free Ds (Figure S7, "no Ft ICD"). So this type of disc acts like a *ft- ds-* double mutant with free Ds. Simulated discs with this form of Ft overgrow more than *ft-* mutants but less than *ft- ds-* double mutants, consistent with experimental results.

Similarly, we can consider FtΔECD, a form of Ft that has its intracellular domain but is missing its extracellular domain (ECD), presumably preventing it from binding to Ds. Experimentally, discs that express this form of Ft rather than wild-type Ft grow slightly larger than wild-type discs [11]. In our model, this version cannot form bonds with Ds (leading to a high effective bond distribution asymmetry), but all of its Ft and Ds will be free (resulting in a strong penalty for leftover Ft and Ds). Discs with this form of Ft, then, will overgrow due to the lack of Ft-Ds binding, but the large amounts of free Ft and Ds mean that they will grow more slowly than *ft-* discs (Figure S7, "no Ft ECD").

Experimentally, wing discs that are mutant for Dpp undergrow [32, 33]. In the model, the morphogen plays a dual role in the disc's growth rate. Cells grow in direct response to their level of morphogen signaling. In addition, morphogen signaling affects the expression pattern of Ds, which in turn affect the degree of Ft-Ds bond asymmetry in a given cell. Discs with a dramatically decreased morphogen concentration, then, will tend to undergrow. The reason for this is twofold; first, the lack of autonomous morphogen signaling will lower the growth rate. Second, because morphogen signaling is low, these discs will express Ds at a uniformly high rate, leading to highly symmetric bond distributions and thus dramatic undergrowth (Figure S7, "no morphogen") in agreement with experiment [34].

Experimentally, discs with uniformly high Dpp signaling overgrow, suggesting that the autonomous effects of Dpp signaling dominate in this case [10, 20, 33]. In our model, a disc with uniformly high morphogen expression will experience more overall morphogen signaling, but it will also lack a spatial gradient in Ds expression, as it will express Ds at uniformly low levels. We would expect the net effect on the disc's growth rate to be parameter-dependent, as the two effects oppose one another. For the set of parameters we use in the simulation, discs with uniform morphogen signaling overgrow compared to the wild-type disc which is qualitatively consistent with experiment [20, 33] ("uniform morphogen" in Figure S7). We also found that both Ft-Ds bond asymmetry and growth rate were elevated near steep discontinuities in morphogen, Fj, or Ds activity (see Figures S26-S31), in agreement with experimental results [10, 12, 35].

8.2 Bond asymmetry with moving and stationary fronts

The outward movement of the front can polarize cells throughout the entire wing pouch, causing an increasing fraction of the tissue to have asymmetrically distributed bonds. Figure S8 shows the degree of bond asymmetry as a function of radial position at different times in the simulation. At the beginning of a run of the simulation, the concentration profile of Ds will be steepest near the front and spatially flat far from the front. This means that initially, Ft-Ds bonds will be asymmetrically distributed only for cells near the front (Figure S8A). As the front moves outward, cells near the front's new position will form new bonds with an asymmetric distribution because their expression rate of Ds has a large spatial gradient. Cells inside the front (i.e., within the central plateau) will have Ds expression rates that vary more slowly in space as the front moves away from them, but their asymmetric bond arrangement will persist after the front has passed. Over the course of the run, more and more cells will have asymmetrically distributed bonds because the front has passed over them (Figure S8B-C). Eventually, this region of higher bond asymmetry will encompass most of the wing primordium (Figure S8D). This result is consistent with experiments, which show that cells throughout the wing pouch, not only those near the edge of the primordium, exhibit an asymmetric distribution of Ft and Ds, as well as a localization of Dachs to the distal side [15, 16].

For the sake of comparison, we also show a simulated disc with a stationary Ds expression front that stays roughly two-thirds of the way between the disc's center and edge. Again, cells that begin near this front exhibit high bond asymmetry (Figure S8E), but now the front does not sweep over a large fraction of the disc. (The front does gradually move with respect to some individual cells due to growth of the wing disc and faster proliferation near the front, as shown in Figure S8F-G). At the end of the run (Figure S8H), the bump in bond asymmetry has shrunk over time, leaving cells near the front with relatively symmetrically distributed bonds. This indicates that movement of the front is necessary to induce bond asymmetry in the entire wing pouch, particularly near the front.

8.3 Dependence on bond dissociation and free Ft/Ds degradation

Under normal conditions in our model, the distribution of Ft-Ds bonds around the periphery of a cell persists well after the Ds front has moved away, constituting a memory of local Ds and Fj expression patterns. This occurs because the processes of Ft/Ds/Fj expression and degradation, bond formation and dissolution, and Ft/Ds phosphorylation and dephosphorylation happen at finite rates, with time scales on the order of hours. We assume that dissolution of the Ft-Ds bond is necessary for, and independent of, Ft and Ds degradation. (This assumption is based on the idea that Ft and Ds molecules would need to dissociate before they could be endocytosed and degraded.) So the rate at which bound Ft and Ds are degraded goes as the product of the rate of dissolving existing bonds and the degradation rate of free Ft and Ds. In our model this product is $(0.08)^2 = 6.4 \times 10^{-3}$ per minute, which means that less than 1% of the bound Ft and Ds are unbound and then degraded per time step, so it takes on the order of hundreds of time steps (i.e., several hours) to turn over all

the bonds on a cell. This time scale is comparable to the time scales of the movement of the front and of the cell cycle. This means that the arrangement of bonds around a cell could persist well after the cell was no longer near the front. So even when a cell and its neighbors are in a region where the local Ds concentration is spatially rather uniform, the cells can still have asymmetrically distributed bonds that were formed when the local Ds gradient was steep. In our model, this allows Ft-Ds bonds to be asymmetrically distributed on cells throughout the wing primordium, not just on cells near the front. As we will show, if the time scales of bond dissociation or free Ft/Ds degradation are substantially altered, the disc may respond differently to the movement of the Ds front.

The overall growth rate of the disc is fairly robust to changes in the bond dissociation rate, largely because the binding algorithm will cause most bonds to reform quickly. The major effects of changes in this parameter, then, are to alter the rate at which Ft and Ds that are less likely to form bonds (i.e., unphosphorylated Ft and phosphorylated Ds) are lost and to alter the responsiveness of cells to the movement of the Ds front. When the dissociation rate is increased by a factor of 10 as in Figure S9, the pattern of polarization is very similar to that seen in wild-type (see Fig 3 in the main text), but the overall growth rate is slightly faster due to increased average bond asymmetry.

When the bond dissociation is decreased by a factor of 10 (Figure S10, panels A-H), the disc is slower to respond to both the movement of the front and the creation of new cell-cell interfaces. Thus, the region of strongest polarization does not move with the front and the populations of unphosphorylated Ft and phosphorylated Ds have longer average lifetimes. However, the average degree of polarization increases over time as cells divide. These opposing effects lead to a slightly faster growth rate than in wild-type. Note that the net change in growth rate depends on the degree to which the dissociation rate is reduced. For example, if the rate is reduced by a factor of 2 rather than 10 (Figure S10, panels I-P), the average growth rate is slower than in wild-type.

The strongly increased polarization in Figure S11 (missing arrows in panels A-D, dark blue regions in panels E-H) is due to some cell-cell interfaces lacking Ft-Ds bonds entirely because of the increased degradation rate. Because Ft and Ds are depleted in some cells faster than they are expressed, particularly near the center of the pouch where Ds expression is slower, these cells are treated by the model as having very strong bond distribution asymmetry and thus proliferate much faster than other cells. Experimentally, Ft and Ds are observed at all cell-cell boundaries in the disc [15], so this figure is a less accurate picture of the wild-type disc.

When the degradation rate is slowed (Figure S12), the overall pattern of polarization is similar to wild-type, but the maximum value of bond asymmetry is lower. Because degradation happens more slowly in this case, individual cells experience slower turnover of their Ft and Ds populations, making them less responsive to the movement of the Ds expression front and slowing proliferation slightly.

The main constraints on the bond dissociation and free Ft/Ds degradation rates are then twofold. These rates should be slow compared to bond formation and Ft/Ds expression (which in the simulation occur on the order of minutes), so that a cell's populations of Ft

and Ds experience little turnover on short time scales and do not decline to zero. The rates of dissociation and degradation should be fast compared to the cell cycle and the movement of the front (which in the simulation occur on the order of hundreds of minutes), so that a cell's bond arrangement can respond to the creation of new interfaces and changes in Ds expression. Thus, as long as these rates fall between these two time scales (e.g., on the order of 10 minutes), the simulation is fairly robust to changes in these parameters. The sole exception is increases in the free Ft/Ds degradation rate, which cannot be substantially faster than the rate of new Ft/Ds expression without eventually reducing these populations to zero.

8.4 Dependence on the Ft-Ds binding rate

The retention of a cell's Ft-Ds bond arrangement after division depends in part on the assumption that the number of Ft-Ds bonds at a new interface quickly reaches a steady state. This assumption in turn requires the time scale of Ft-Ds binding to be fast compared to the length of a cell cycle. Here, we examine what happens when this restriction is loosened.

Figure S13 shows the direction and magnitude of Dachs localization in discs where the binding probabilities are divided by 10, greatly reducing the number of bonds formed per time step. The bond weight parameters described in Section 3.3 are reduced by a factor of 10, thus increasing by roughly an order of magnitude the time scale on which the number of bonds at a given interface reaches a steady state. All other parameters remain the same as in wild-type, including the rate at which free Ft and Ds are degraded; this means that the degradation rate of Ft and Ds is fast relative to the binding rate. The overall pattern of Dachs localization remains distal near the Ds front. However, many cells within the front have a much greater magnitude of Dachs polarization compared to wild-type, likely because newly formed cell-cell interfaces spend more time having far fewer Ft-Ds bonds than other interfaces, which lowers our "minimum fraction" metric for Dachs polarization. Furthermore, cells near the center of the disc lack Ft-Ds bonds altogether, since they have very little Ds (particularly phosphorylated Ds) available to form bonds. This suggests that the rate of bond formation should be comparable to the rate of Ft and Ds expression and degradation, so that a large fraction of a cell's Ft and Ds populations forms bonds with adjacent cells.

8.5 Dachs localization under altered Fj and Ds expression

As shown in Figure S7, discs that uniformly overexpress Fj, Ds, or both tend to undergrow compared to wild-type discs. We show here that this undergrowth is the result of a more symmetric distribution of Ft-Ds bonds and consequently weaker Dachs polarization.

Figures S14, S15, and S16 show the direction and magnitude of Dachs localization in discs that uniformly overexpress Fj, Ds, or both, respectively. In all three cases, the overall magnitude of Dachs polarization is smaller than that in the wild-type disc (see Fig 3 in the main text). As in Figs 3 and 4 in the main text, the arrowheads in panels A-D in these figures represent the average direction of Dachs localization in nearby cells, while the colored

markers in panels E-H represent the magnitude of Dachs polarization. In Figure S14, cells in a disc uniformly overexpressing Fj still exhibit a distal localization of Dachs, especially near the front of Ds expression, but this effect is weaker than in a wild-type disc. (Altering the rate of uniform overexpression gives similar results of lowered bond distribution asymmetry but distally localized Dachs near and within the Ds expression front.) In Figure S15, when Ds is uniformly overexpressed, cells show only a weak proximal-distal polarization resulting from the graded Fj profile alone. When both Fj and Ds are uniformly overexpressed (Figure S16), Dachs localization is weak and randomly oriented, since all cells have similar amounts of Ft, Ds, and Fj. Note that for these figures, we deliberately use expression rates of Fj and Ds that are much higher than in wild-type, so that we can compare the results to experiments in which Ds and Fj are strongly overexpressed via coexpression with tubulin [10]. However, in general, the degree of polarization likely exhibits some dependence on the overall levels of Fj or Ds [36].

Figure S17 shows the direction and magnitude of Dachs localization in a disc with no Fj. The average magnitude of polarization is very small, since cells with no Fj (and thus no phosphorylated Ft) cannot form bonds as readily as cells with Fj. Note that cell polarization is still distal in the vicinity of the Ds front, but is not retained for long after the front has passed. Because much less of the Ft in these cells is phosphorylated, the overall rate at which new Ft-Ds bonds are formed is much slower than in a wild-type disc, leading to weaker polarization.

To examine the effects of the Fj profile on Dachs localization in finer detail, we calculated the average Dachs localization under different conditions of Fj expression. Figure S18 shows the average magnitude (panels A, C, and E) and direction (panels B, D, and F) of Dachs localization with a variety of Fj profiles. In particular, we varied the spatial gradient of Fj (panels A-B), minimum concentration of Fj at the edge of the disc (panels C-D), and concentration when Fj is expressed uniformly (panels E-F). We also examined these Fj profiles with a moving Ds front (black), a stationary Ds front (blue), and uniform Ds expression (red). All parameters besides those mentioned were the same as in wild-type. The average magnitude of Dachs polarization was substantially lower when Fj levels were low (i.e., on the order of 0.1) throughout the disc, but was otherwise fairly robust to changes in the Fj profile. Furthermore, Dachs polarization was substantially stronger with a moving front than with a stationary front, and weakest when Ds was expressed uniformly, suggesting that the movement of the Ds front is a major contributing factor to Dachs polarization.

8.6 Contribution of Fj to Dachs localization when the Ds front is stationary

To differentiate the effects of Ds and Fj when the Ds front is stationary, we removed the spatial gradient of Fj in runs with a stationary Ds front. Figure S19 shows the pattern of Dachs localization when the Ds front is stationary and Fj is expressed uniformly. The overall polarization pattern is broadly similar to that shown in Fig 4 in the main text, but with a lower average magnitude of polarization. Dachs polarization remains most pronounced

near the Ds front, while cells that have never had the front pass over them have randomly localized Dachs. In contrast, Figure S20 shows the pattern of Dachs localization when the Ds front is stationary and Fj is absent. Although cells near the Ds front do, on average, have distally localized Dachs, the magnitude of this polarization is very weak. Again, cells further from the front have randomly localized Ds. These results suggest that the Ds front alone is sufficient to give rise to distalward Dachs localization nearby, but the magnitude of this polarization is higher when Fj is present and higher still when Fj is spatially graded, with high concentrations in the center of the disc and low concentrations at the edge.

8.7 Varying the length of the time step

Some aspects of the model have a dependence on the length of the time step. In particular, the growth rate is defined in terms of the ratio of a cell's radius at a given time step to its radius at the previous step. Similarly, the integral feedback mechanism involves integrating the value of a function at all previous time steps to calculate its value at the next time step. Furthermore, the formation of new Ft-Ds bonds takes different amounts of time depending on the phosphorylation state of the Ft and Ds proteins involved. Thus, the length of a time step needs to be small compared to the time scale for bond formation, so that bonds that are less likely to form, e.g., Ft-DsP, will require many more time steps than the other types of bonds to reach its steady-state number at each interface. The relative rates of bond formation (measured in inverse time steps) can then be affected if the length of the time step is not much smaller than the time scale of bond formation. For example, Ft-DsP bonds normally take much longer than the other bond types to reach a steady-state number at each interface. When the time steps are not small enough, this distinction diminishes. In particular, if the time steps were much longer than 1 minute, e.g., 100 minutes, then we would expect all four bond types to form at the same effective rate, and reach a steady-state number in a single time step. Thus, the longer the time step, the less the difference in the number of time steps required to reach steady state values for the different types of bonds and thus, the less pronounced the effects of Fj.

Figure S21 shows the direction and magnitude of Dachs localization when the length of the time step is doubled from 1 minute to 2 minutes. The simulation is then run for 2100 simulated minutes or 1050 time steps. The pattern of Dachs localization is very similar to that under normal conditions (i.e., 1-minute time steps) but the average growth rate is slightly slower, resulting in an average cell cycle time of roughly 680 minutes (compare to 600 minutes for normal time steps).

Figure S22 shows the direction and magnitude of Dachs localization when the length of the time step is halved from 1 minute to 30 seconds. The simulation is then run for 2100 simulated minutes or 4200 time steps. The overall pattern of Dachs localization is again retained, but the average magnitude of polarization is higher. The average growth rate is faster as a result, resulting in an average cell cycle time of roughly 530 minutes.

8.8 Patterns of cell proliferation with a moving front

Figure S23 shows the overall growth rate (black) as well as the contribution to the growth rate due solely to morphogen signaling (green) or solely to bond asymmetry (blue) as a function of radial position within the disc. The contribution of morphogen signaling corresponds to the value of the growth rate in the absence of bond asymmetry, i.e., if the expression $(1+C_{Ft}x_{Ft,i})(1+C_{Ds}x_{Ds,i})$ in Eq. (S20) were equal to 1 for all cells. Similarly, the contribution of bond asymmetry corresponds to the value of the growth rate in the absence of morphogen, i.e., if the expression $(1 + C_M x_{M,i})$ in Eq. (S20) were equal to 1 for all cells. (These growth rates are still penalized for having leftover free Ft and Ds, in both cases.) The quantities plotted in Figure S23, then, are the hypothetical frequency of cell divisions if the growth rate had these values.

Cells near the Ds expression front (vertical black line) exhibit faster growth due to bond asymmetry, while cells near the center exhibit faster growth due to morphogen signaling. As the expression front moves outward, the wing primordium (i.e., the area of the disc lying inside the front) exhibits complementary patterns of growth signaling due to bond asymmetry and morphogen concentration. The net effect from these two signals is comparatively uniform growth throughout the wing primordium. Because these patterns counterbalance each other, the overall growth rate should be more uniform throughout the wing pouch than the contributions from either morphogen signaling or bond asymmetry alone.

The profile of growth rate is not a direct reflection of the spatial profile of the morphogen concentration or of the bond asymmetry because the growth rate is subject to integral feedback. As a result the growth rate gradually adapts to a constant signal. So cells that have had asymmetric bonds for most of the run will grow more slowly than cells that have recently undergone an increase in bond asymmetry. This means that the cells in the center (the distal cells) will respond less to their bond distribution asymmetry than the cells at the front (the proximal cells). The overall effect is a signaling gradient; growth due to bond asymmetry will be stronger near the front and weaker at the center of the disc.

Morphogen signaling in our model is strongest at the center of the disc and weakest at the edge, and its amplitude increases over time. The growth rate used in the simulation is affected by the integral feedback adaptation mechanism described above. This means that regions where the morphogen concentration increases rapidly (i.e., the center of the disc) experience more growth due to morphogen signaling than regions where the concentration increases slowly (i.e., the disc's periphery). The result is a profile of morphogen-driven growth that is still fastest in the center and slowest at the edge. This pattern is roughly complementary to the pattern of growth induced by bond asymmetry.

8.9 Patterns of cell proliferation with a stationary front

When the Ds expression front is stationary (in terms of its relative position between the disc's center and edge), it does not sweep over a large fraction of the disc. Rather, it remains close to the same set of cells and their descendants for most of the run. The only relative motion between the cells and the front in this case is due to cell proliferation; cells can push

their neighbors aside as they grow and divide. A cell in this disc, then, will express roughly constant amounts of Ds for the duration of the run. In this case, only an annulus of cells near the front experience elevated bond asymmetry and the resulting growth. This growth is further reduced due to the adaptation mechanism, since bond asymmetry is consistently high in these cells. If the morphogen profile is unchanged from wild-type, however, morphogen signaling will continue to drive cell growth near the disc's center. The result is a pattern of cell proliferation that is dominated by morphogen signaling with relatively little contribution from bond asymmetry (Figure S24).

8.10 Clones with elevated morphogen signaling

We also examined how bond asymmetry and growth rate might be affected by artificial expression boundaries. To this end, we marked a circular cluster of cells in the simulation that was concentric with the whole disc, and altered either morphogen concentration or the Fj or Ds expression rate in the marked cells. We then examined the degree of bond asymmetry and the growth rate as functions of distance from the cluster's edge. Furthermore, we varied the radius of this "clone" and compared the results when the clone boundary lay inside versus outside the wing primordium boundary.

Experimentally, when elevated Dpp signaling is induced in a clone of cells (e.g., via constitutively active Tkv), nonautonomous effects on cell proliferation are visible via BrdU incorporation [12]. Cells in the clone express both more Fj and less Ds than cells adjacent to the clone [10], presumably leading to elevated bond distribution asymmetry and thus faster proliferation near the clone boundary. To study this, we did simulations to compare a wild-type disc to a disc with a circular clone. The edge of the clone and the wing disc boundary are concentric circles. The clone has morphogen signaling at a level, constant in space and time, that is higher than that of wild-type (analogous to cells expressing activated Tkv). Outside the clone, the cells are wild-type and are subjected to an exponentially decaying morphogen profile. Figure S25 shows the degree of bond distribution asymmetry at different radial positions within the simulated discs. When the clone is large enough that its boundary lies outside where the Ds expression front would be in the wild-type disc, a dramatic increase in bond distribution asymmetry can be seen both inside and outside the vicinity of the clone's border. This is because the cells of the clone, which have low levels of Ds, are juxtaposed next to the Ds-expressing cells of the disc's periphery, rather than the cells in the wing primordium as is the case when the clone boundary is inside the Ds expression front. Figure S26 shows the corresponding cell growth rates when a morphogen-overexpressing clone is induced; in addition to the increased growth seen due to the autonomous effects of morphogen signaling, regions with increased bond asymmetry also proliferate faster.

8.11 Fj-expressing clones

Discs containing clones of cells that overexpress Fj or Ds exhibit nonautonomous proliferation near their edges [10]. This effect is most pronounced in the region of the disc that does not

normally produce much of the protein that the clone is overexpressing. (For example, clones that overexpress Fj exhibit more nonautonomous growth when they are in the periphery of the disc, which normally expresses low levels of Fj.) We increased (and made uniform) the rate of Fj expression in a circular cluster of cells concentric with the whole disc, i.e., a circle whose center coincides with the center of the disc. We then examined the degree of bond asymmetry (Figure S27) and the growth rate (Figure S28) as functions of distance from the circle's edge. By altering the radius of this "clone", we can examine how the position of the clone boundary changes the nonautonomous effects on growth. In our model, clusters of cells overexpressing Fj show the most nonautonomous growth in the outer (proximal) regions of the disc that express little endogenous Fj.

When elevated Fj expression is induced in a clone concentric with the disc, the resulting Ft-Ds bond asymmetry and cell growth are comparable to those in the wild-type disc when the clone's edge is within the Ds expression front. In this case, the cells in the clone do not have much more Fj than is expressed endogenously in this region, so the behavior is much like that in the wild-type disc (Figures S27 and S28, panels A and B). However, when the clone's boundary lies outside the usual Ds expression front, we see increased bond asymmetry and faster growth rates near the clone's boundary (Figures S27 and S28, panels C and D). In this case, the cells outside the clone's boundary have much less Fj than those within the clone, leading to an increase in bond asymmetry and an increase of growth rate among cells near the boundary, consistent with experimental results [10].

8.12 Ds-expressing clones

In our model, a uniformly Ds-expressing clone concentric with the disc exhibits increased bond asymmetry (Figure S29) and increased growth (Figure S30) when the clone's boundary is within the Ds expression front. In this case, the cells just outside the clone's boundary express much less Ds than the cells inside the clone, leading to an increase in bond asymmetry near the boundary. Experimentally, cells near the edge of a Ds-expressing clone are observed to proliferate faster when they are near the disc's center [10]. Our simulations are consistent with this result; Ds clones whose boundaries are near the center of the disc exhibit more proliferation near their edges than Ds clones whose boundaries are near the disc's periphery.

8.13 Neighbor statistics

Gibson *et al.* [21] presented compelling mathematical arguments together with experimental confirmation for why epithelial cells have an average of 6 adjacent neighbors. We examined the distribution in our model of neighbor numbers in a wild-type disc and found that on average, a cell has 6.0 adjacent neighbors (Figure S31). This distribution is broadly similar to the experimental results and simulation cases I and V studied by [1], although the proportion of 5-sided cells is slightly lower at 26.5% (compare to roughly 32% in case I and 33% in case V) and the proportion of 7-sided cells is slightly higher at 21.9% (compare to roughly 17% in case I and 19% in case V). These results suggest that cell growth and division in our model

are indeed fairly rapid compared to cell rearrangement [21].

9 Input and output

Here we describe the information that the simulation needs to begin a run, as well as the information the simulation records for later analysis.

Starting conditions for the simulation, including the progenitors, spatial coordinates, sizes, and velocities of each cell, are read in from an existing text file when the simulation begins. The program then calculates disc-wide quantities, including the disc's starting radius and the location of its center of mass, and builds the neighbor list for all the cells. (For purposes of finding the disc's radius, it assumes a roughly circular cluster of cells; since cell divisions are oriented in all directions and cells stay in a single contiguous cluster, the disc remains roughly circular throughout a run.) Finally, it assigns starting quantities of morphogen, Ft, Ds, and Fj to all cells, assigns each cell's Ft and Ds to phosphorylated and unphosphorylated pools depending on the cell's starting amount of Fj, and creates a starting set of Ft-Ds bonds as described above.

At each time step, the simulation records information about all the cells, including their indices, spatial coordinates, and radii, and the indices of each cell's parent. If the simulation involves marking certain cells as part of a "clone" for later manipulation, it records which cells are in the clone, and the distance of each cell from the clone's edge (in terms of cells). The simulation also records each cell's growth rate and associated quantities, including its amounts of free and bound Ft and Ds; its amount of Fj; the minimum fractions of bound Ft and Ds as used in Equations (S26) and (S27); and $x_{M,i}$, $x_{Ft,i}$, and $x_{Ds,i}$, the contributions of morphogen signaling and Ft and Ds bond asymmetry to the growth rate. It also records the angular position of Dachs localization (i.e., the direction of the neighbor with which the cell has the least bound Ft). Because it contains information on every cell at every time step, this file can become very large, especially for longer runs and runs involving larger discs.

We also need a more compact way to record general information about a single run. The overall size of the disc is fairly consistent from one run to another under the same conditions. However, the location, bond arrangement, and growth rate of any given cell will not be fixed between runs. To determine the overall pattern of bond asymmetry and growth, then, it can be useful to collect information over many runs. To do this, the simulation can record more general data intended to be averaged. Every 100 time steps, the simulation records the location of the front (i.e., the point where the Ds gradient is steepest), and then divides the disc into 50 evenly spaced concentric rings. For each of the rings, it calculates the average growth rate, the average contributions of morphogen signaling and bond distribution asymmetry to the growth rate, and the average Ft asymmetry (i.e., the minimum fraction of bound Ft, adjusted for the number of neighbors). Since the overall size of the disc typically varies by less than 1% between runs, the absolute size of each ring is fairly consistent from one run to another. If this information is appended to an existing file rather than written to a new file each time the program is run, we can examine the average spatial patterns of signaling and growth over many runs. Similarly, every time a cell divides, the location and

time of the division are also recorded in a separate file. Again, by appending this information to an existing file during each run, we can find an average pattern of cell proliferation over multiple runs.

The simulation can be set to run for either a certain number of time steps or until the disc has a certain number of cells. In either case, when the program finishes, the final state of all cells is recorded in the same format used for the input file, so a run can be stopped and then resumed in a later session. The records made during the run can be read by a set of MATLAB scripts to generate graphs and movies. Using these scripts, we can show the spatial patterns of, for example, Dachs localization or cell proliferation.

10 Parameters

These are the parameters used in the simulation, along with their values and the equations in which they appear.

The parameters most responsible for the "memory" effect are the rates of bond dissolution and free Ft/Ds degradation, which are discussed in detail in the main text. Here, we examine the remaining parameters, our rationales for choosing their values, and their effects on our results.

10.1 Morphogen profile

t_0, the characteristic time for the increase of the morphogen amplitude, is based on experimental results [4] that suggest that the Dpp concentration in a given cell increases by roughly 50% every several hours. The parameters A, C_0, and M_c determine the spatial scale of the morphogen profile and the starting location of the front. A, set to 4, is the ratio of the radius of the disc R to the morphogen profile's decay length; in other words, the decay length is always 1/4 the radius of the disc. This is based on results [4] that show that the decay length is roughly 1/9 the radius of the disc; however, our simulation primarily represents cells that go on to form the wing pouch, rather than the entire disc.

The location of the front is the radial distance at which the morphogen concentration $[M]$ is equal to M_c. By choosing the ratio $C_0/M_c = 3$, we set the starting location of the front to $\ln(3)/A$ times the disc's radius, or roughly 0.275 times the disc's radius. We choose this position so that the front can sweep over the majority of the disc during the simulation while acknowledging that the wing primordium is already present at the beginning of the third instar [37].

The overall amplitude of the morphogen profile C_0, along with the parameters involved in calculating the growth rate, determines how cells respond to local morphogen signaling.

10.2 Growth rate

The parameters G_M, G_{Ft}, and G_{Ds} determine how growth signaling from the morphogen and from the Ft and Ds bond distribution asymmetry are affected by adaptation. More

specifically, these parameters determine the time scales over which the integral feedback mechanism takes effect. Lower values here mean that adaptation is slow (so the growth rate responds largely to the amount of the signal), while higher values mean that adaptation is fast (so the growth rate responds largely to the rate of change of the signal).

Experimentally, the nonautonomous effects on the growth rate near clones with elevated Dpp signaling or Fj or Ds expression are weaker but still present after roughly two days [10, 12]. This suggests that we should use similar time scales for the adaptation response to Ft and Ds bond asymmetry. For this reason, we set the integral feedback parameters G_{Ft} and G_{Ds} equal to 6×10^{-4} min^{-1}, roughly equal to (28 hours)$^{-1}$. These parameters affect how long the elevated growth rate persists after a cell's bonds become asymmetric (shown in Figure S23), but do not affect the bonds themselves.

Since the growth rate appears to respond to the rate of change of Dpp [4], we choose a higher value of G_M, equal to 2.8×10^{-2} min^{-1}. However, if this value is too high, cells with elevated morphogen signaling will not overgrow, since their amount of morphogen signaling is not changing in time.

We want morphogen signaling and bond asymmetry to have similar contributions to the growth rate. The contribution from morphogen signaling, for example, scales with the product $C_M G_M$; similarly, the contributions from Ft and Ds asymmetry scale with $C_{Ft} G_{Ft}$ and $C_{Ds} G_{Ds}$. We choose values of the C coefficients so that these products each have a similar order of magnitude. For this reason, we use $C_M = 40$ min, $C_{Ft} = C_{Ds} = 3000$ min. Changing these coefficients would affect only the relative contributions of morphogen signaling and bond asymmetry to the growth rate, and again would not alter the bonds themselves.

We want a fairly small penalty for leftover unbound Ft and Ds, because *ft- ds-* double mutant discs overgrow slightly more than either the *ft-* or the *ds-* single mutant. The growth rate for each cell is divided by $1 + U_i$, where U_i is the total number of free Ft and Ds on cell i multiplied by 3×10^{-5}. Since the number of free protocadherins on a cell is typically on the order of 10-100, this means that U is normally much less than 1, making this a very small adjustment to the growth rate.

Finally, the coefficient G_0, which scales the overall growth rate, was chosen to give a cell cycle time of roughly 600 time steps (10 hours), as seen experimentally.

10.3 Cell division

The critical cell area for division is set to 4π μm^2, corresponding to a radius of 2 μm, which is the approximate width of cells in the disc. Altering this parameter would change the average cell size, but not the length of the cell cycle, since cells in the simulation divide when they double in size and then conserve their total area upon division. (Recall that the growth rate for a given cell is defined in terms of the ratio of the cell's radius at time step $n+1$ to its radius at time step n, so the number of steps needed for a cell to double in size should remain constant.) The Hill exponent ℓ is set to the fairly high value of 400 primarily to make the cell division probability function steep, so that cells only divide when they are close to

the critical size. Making this parameter smaller would lead to a slightly greater variety of mitotic cell sizes, but would have little effect otherwise.

The sizes of the daughter cells upon division are adjusted by a random amount between -1% and +1%. This is only to prevent entire clones of cells from dividing simultaneously when every cell in the clone has an equal growth rate. However, this situation only arises in very specific cases (e.g., uniform morphogen signaling in a *ft- ds-* double mutant). So this adjustment has very little effect on the growth of cells under most conditions.

10.4 Neighbor list

The neighbor list is rebuilt when any one cell has moved a total distance equal to 0.2 cell radii, or when 10 time steps have passed, whichever is less. This simply allows the program to avoid rebuilding the neighbor list when it is unlikely to have changed. In practice, this typically means that the neighbor list is rebuilt every 2-3 time steps instead of every time step. Rebuilding the neighbor list more often than once every 2-3 time steps affects only the running time of the simulation, not its results. Rebuilding the neighbor list less frequently means that the adjacency list may fail to reflect the actual cell geometry. (Note that this applies only to rebuilding the entire neighbor list. When a cell divides, the neighbor list will be refreshed only for the potentially affected cells, which is much more efficient than rebuilding the entire list.)

Neighbor pairs that are less than 100 minutes old are not counted for the purposes of calculating bond asymmetry. This is to prevent new interfaces with no Ft-Ds bonds from dramatically increasing the growth rate immediately after cell division, leading to a positive feedback loop. We choose 100 minutes for this parameter because it allows time for the number of bonds at the new interface to reach a steady state but is substantially shorter than the cell cycle time (roughly 600 minutes).

10.5 Bulk forces

The parameters C_{gap} and C_{overlap} determine, respectively, the magnitude of the attractive and repulsive forces between cells. The drag parameter γ is present largely to keep cells from separating too far for the attractive force to take effect, and also damps oscillations. These parameters serve only to keep the cells in a single contiguous cluster and affect the arrangement of Ft-Ds bonds only inasmuch as they maintain cell adjacency.

Note that the relative strengths of these two forces do help determine the effective size of a cell (i.e., the distance between a cell and its neighbor). The size of a cell compared to the length scale of the Ds expression pattern is what gives rise to bond distribution asymmetry. However, as long as the transition region (where the Ds gradient is steepest) has a size on the order of several cell diameters or smaller, the Ft-Ds bonds around a cell do become more asymmetrically distributed while that cell is within the transition region.

We also specify that neighboring cells with a gap (surface-to-surface distance) greater than 0.8 μm do not interact, whether to attract one another or to form Ft-Ds bonds. Neigh-

bors with a gap this large only occur near the edge of the disc, where the Delaunay triangulation algorithm can occasionally consider cells to be adjacent when they are not touching. Making this parameter larger would cause the program to ignore more of these cases and form bonds between cells that are not actually adjacent. If it were substantially smaller, the program would ignore pairs of cells that were adjacent but had a small gap between them due to local tension.

10.6 Ft/Ds expression

The parameters Ft_{max}, $A_{Ds,initial}$, $R_{Ds,max}$, Ds_{min}, and Ds_{range} determine the initial amounts of Ft and Ds in all cells and the amounts of additional Ft and Ds expressed per time step. The initial amount of Ft, Ft_{max}, is 1000 proteins per cell, and the initial amount of Ds in a cell is between 200 and 1000 proteins, depending on the cell's location. These amounts allow for $\sim$100 proteins to be present at each cell-cell interface. If there are substantially fewer proteins than this, individual interfaces may have no Ft or Ds present, a situation not seen experimentally in wild-type discs [15, 16]. Having substantially more Ft or Ds proteins at each interface has little effect on the overall distribution pattern of Ft-Ds bonds, but may be unrealistic.

The rate of Ft expression is 20 proteins per cell per minute, while the rate of Ds expression is between 4-20 proteins per cell per minute. We choose these expression rates in part because they are comparable to the degradation rates of free Ft and Ds that we use (8% of the free Ft and Ds pools per minute), which means that the number of Ft and Ds on each cell starts fairly close to its steady-state value. Furthermore, the expression rates of new Ft and Ds also affect the time scale on which a cell can build up new Ft-Ds bonds, e.g., at new cell-cell interfaces created by cell division. In the model, this affects the bond distribution asymmetry and proliferation rates. For cells to recover their polarization after dividing, the time scale over which Ft-Ds bonds accumulate at a new interface needs to be short compared to the cell cycle. Similarly, for cells to respond to the movement of the Ds expression front, the time scale over which the number of Ft-Ds bonds at an interface changes needs to be short compared to the time scale for the movement of the front. Thus, the time scales for Ft and Ds expression are restricted in much the same way as the time scales for Ft-Ds bond dissociation and free Ft/Ds degradation.

If the expression rate is increased substantially, e.g., by a factor of 10, then the typical number of free Ft and Ds per cell will also be greater by an order of magnitude, tending to slow cell proliferation due to the growth rate penalty for free Ft and Ds. Furthermore, when the expression rate is faster, the number of bonds at an interface takes longer to reach a steady state, meaning that cells are slower to respond to changes in Ds expression (with time scales $\sim$100 minutes rather than $\sim$10 minutes). As a result, the movement of the Ds expression front has a weaker effect on Dachs polarization, and the overall cell proliferation rate is slower.

If the expression rate is slowed by a factor of 10, the number of bonds at a typical interface is $\sim$10, and the number of bonds at an interface reaches a steady state in < 10 minutes.

In this case, cells respond quickly to the movement of the Ds expression front, but also to the bond distribution asymmetry in their neighbors caused by cell division. The result is dramatically increased average bond asymmetry and irregular Dachs localization.

The Hill exponent m describing the dependence of the starting amount and expression rate of Ds on the morphogen concentration is set to 10. This gives rise to a fairly steep gradient of Ds, giving the transition region a width on the order of 10 μm, consistent with images of Ds expression in the disc. If this exponent is much smaller (e.g., unity), the Ds profile will resemble a straight line rather than a sigmoid curve in much of the disc, and the transition region will effectively span the entire disc. If the exponent is much greater (e.g., 100), the sigmoid curve will be much steeper, resembling a step function.

10.7 Fj expression

The parameters $R_{Fj,max}$, $[\text{Fj}]_{min}$, and Fj_{slope} determine the rate of Fj expression. Fj is present only to determine the phosphorylation rates of Ft and Ds, and does not directly affect the growth rate. Our choices of $[\text{Fj}]_{min}$ and Fj_{slope} mean that the starting amounts of Fj in the center and edge of the disc differ by roughly a factor of 10, with the critical value $[\text{Fj}]_c$ in between. This allows the phosphorylation states of Ft and Ds to vary spatially across the disc. Similarly, the expression rate of Fj (determined by $R_{Fj,max}$, $[\text{Fj}]_{min}$, and Fj_{slope}) also varies spatially by a factor of 10, increasing as the disc grows. Changing these parameters would change the phosphorylation profile, altering the radial position at which Ft and Ds go from being mostly phosphorylated to being mostly unphosphorylated. If this radial position lies near the center of the disc or outside its edge, then most cells in the disc will have the same phosphorylation state, causing an overall reduction in Ft-Ds bond asymmetry.

The degradation rate of Fj, $k_{d,Fj}$, is equal to 0.05 min^{-1}. Since the maximum rate of Fj production, $R_{Fj,max}$, is also 0.05 min^{-1}, the starting concentrations of Fj are then fairly close to their steady-state values. (We measure the Fj concentration in a cell on a continuous scale, rather than tracking individual proteins as with Ft and Ds.)

Note that because the base expression and degradation rates ($R_{Fj,max}$ and $k_{d,Fj}$) are equal, each cell's starting concentration of Fj is already close to its steady-state value. In other words, the steady-state amount of Fj is very close to $[\text{Fj}]_{min} + \text{Fj}_{slope}(r_{\max} - r_i)$. As long as the degradation and base expression rates are equal, our overall results are robust to changes in their value. The main effect of changing the values of these two rates (while keeping them equal) would be to alter the time scale on which a cell reaches a steady-state concentration of Fj.

10.8 Ft-Ds phosphorylation and dephosphorylation

We choose the parameters P_{offset}, C_1, and C_2 such that the probability of individual Ft or Ds proteins being bindable at the beginning of the simulation is between 0.1 and 0.9. These probabilities need to be substantially different (close to 0 and 1, respectively) in order for the Fj profile to have a significant effect on bond asymmetry.

The critical value of [Fj], $[\text{Fj}]_c$, determines the rates of Ft/Ds phosphorylation as a function of the local Fj concentration. (The phosphorylation profile is sigmoidal with an inflection point where $[\text{Fj}] = [\text{Fj}]_c$). The radial position of a cell where $[\text{Fj}]_i = [\text{Fj}]_c$ should lie at some intermediate position between the center and the edge of the disc, so that the Ft/Ds phosphorylation profiles can vary appreciably with radial position. Similarly, the Hill exponent in Equation (S14) partially determines the effective spatial steepness of the phosphorylation profile. However, the steepness of the Fj expression front also determines this.

The parameter D determines the dephosphorylation rates of Ft and Ds. A fraction equal to D times the amount of phosphorylated Ft and Ds is dephosphorylated each minute. Since $D = 1.28 \times 10^{-3}$ min^{-1}, dephosphorylation is fairly slow compared to Ft/Ds expression, binding, and degradation. (To our knowledge, there is no phosphatase that acts specifically on Ft and Ds, so we would expect dephosphorylation to be slow.)

10.9 Ft-Ds binding

The rate of Ft-Ds binding in our model depends on the phosphorylation states of the Ft and Ds proteins involved, meaning that bonds of different types are formed on different time scales. Past experiments have shown that the presence of Fj makes Ft more likely and Ds less likely to form bonds, suggesting that phosphorylated Ft (FtP) and unphosphorylated Ds form bonds more readily [5–7]. However, more recent experiments have shown that when Fj is removed, Ds becomes less, not more, stable at junctions [5]. This suggests that the effect of Fj on Ft is more important than its effect on Ds, and that Ft-P and Ds-P can still bind to one another. Furthermore, cells whose Ft was unable to be phosphorylated formed far fewer bonds than cells whose Ft could be phosphorylated, and this result was not significantly affected by the presence or absence of Fj [5].

We then choose our bond weights such that the phosphorylation state of Ft is the most important factor, followed by the phosphorylation state of Ds. Thus, FtP-Ds bonds form most readily, followed by FtP-DsP bonds. Bonds with unphosphorylated Ft (Ft-Ds and Ft-DsP) are much less likely to form, regardless of the phosphorylation state of Ds.

10.10 Specific conditions

When Ds is overexpressed in the simulation, we use starting populations and expression rates of Ds that are 10 times their maximum wild-type values. In corresponding experiments, Ds is typically coexpressed with tubulin, allowing it to be expressed at many times its normal rate [10, 11]. Overexpressing Ds at a higher rate in our simulation would lead to slightly slower growth due to the excess of free Ds, but would have no other effect, because the number of Ft-Ds heterodimers would be limited by the amount of available Ft.

When Fj is overexpressed, we use a starting amount and expression rate that is roughly 10 times the maximum starting wild-type value. As with Ds, this is intended to parallel experiments in which Fj is coexpressed with tubulin. This means that nearly all Ft and Ds will be phosphorylated at steady state. Higher rates of Fj expression give very similar

results due to this saturation. Lower rates of uniform Fj expression (e.g., comparable to wild-type Fj expression rates) give rise to discs with slightly lowered bond asymmetry and proliferation rates compared to wild-type.

When the front is stationary, we choose a location that is equal (in terms of relative radial distance) to the ending location of the front in the corresponding wild-type run. This is so that the results from both sets of runs can be compared more directly.

References

[1] Farhadifar R, Röper JC, Aigouy B, Eaton S, Jülicher F (2007) The influence of cell mechanics, cell-cell interactions, and proliferation on epithelial packing. Current Biology 17: 2095–2104.

[2] Rapaport D (1997) The Art of Molecular Dynamics Simulation. Cambridge University Press.

[3] Mao Y, Tournier AL, Bates PA, Gale JE, Tapon N, et al. (2011) Planar polarization of the atypical myosin Dachs orients cell divisions in *Drosophila*. Genes and Development 25: 131–136.

[4] Wartlick O, Mumcu P, Kicheva A, Bittig T, Seum C, et al. (2011) Dynamics of Dpp signaling and proliferation control. Science 331: 1154–1159.

[5] Hale R, Brittle AL, Fisher KH, Monk NAM, Strutt D (2015) Cellular interpretation of the long-range gradient of Four-jointed activity in the *Drosophila* wing. eLife 4: e05789.

[6] Brittle AL, Repiso A, Casal J, Lawrence PA, Strutt D (2010) Four-jointed modulates growth and planar polarity by reducing the affinity of Dachsous for Fat. Current Biology 20: 803–810.

[7] Simon MA, Xu A, Ishikawa HO, Irvine KD (2010) Modulation of Fat:Dachsous binding by the cadherin domain kinase Four-jointed. Current Biology 20: 811–817.

[8] Mao Y, Kucuk B, Irvine KD (2009) *Drosophila* lowfat, a novel modulator of Fat signaling. Development 136: 3223–3233.

[9] Strutt H, Strutt D (2002) Nonautonomous planar polarity patterning in *Drosophila*: Dishevelled-independent functions of Frizzled. Developmental Cell 3: 851–863.

[10] Rogulja D, Rauskolb C, Irvine KD (2008) Morphogen control of wing growth through the Fat signaling pathway. Developmental Cell 15: 309–321.

[11] Matakatsu H, Blair S (2006) Separating the adhesive and signaling functions of the Fat and Dachsous protocadherins. Development 133: 2315–2324.

[12] Rogulja D, Irvine KD (2005) Regulation of cell proliferation by a morphogen gradient. Cell 123: 449–461.

[13] Yi TM, Huang Y, Simon MI, Doyle J (2000) Robust perfect adaptation in bacterial chemotaxis through integral feedback control. Proceedings of the National Academy of Sciences 97: 4649–4653.

[14] Burke R, Basler K (1996) Dpp receptors are autonomously required for cell proliferation in the entire developing *Drosophila* wing. Development 122: 2261–2269.

[15] Ambegaonkar AA, Pan G, Mani M, Feng Y, Irvine KD (2012) Propagation of dachsous-fat planar cell polarity. Current Biology 22: 1302–1308.

[16] Brittle A, Thomas C, Strutt D (2012) Planar polarity specification through asymmetric subcellular localization of Fat and Dachsous. Current Biology 22: 108.

[17] Mao Y, Rauskolb C, Cho E, Hu WL, Hayter H, et al. (2006) Dachs: an unconventional myosin that functions downstream of Fat to regulate growth, affinity and gene expression in *Drosophila*. Development 133: 2539–2551.

[18] Baena-López LA, Baonza A, García-Bellido A (2005) The orientation of cell divisions determines the shape of *Drosophila* organs. Current Biology 15: 1640–1644.

[19] Dupont S, Morsut L, Aragona M, Enzo E, Giulitti S, et al. (2011) Role of YAP/TAZ in mechanotransduction. Nature 474: 179–183.

[20] Schwank G, Tauriello G, Yagi R, Kranz E, Koumoutsakos P, et al. (2011) Antagonistic growth regulation by Dpp and Fat drives uniform cell proliferation. Developmental Cell 20: 123–130.

[21] Gibson MC, Patel AB, Nagpal R, Perrimon N (2006) The emergence of geometric order in proliferating metazoan epithelia. Nature 442: 1038–1041.

[22] Guibas L, Stolfi J (1985) Primitives for the manipulation of general subdivisions and the computation of Voronoi diagrams. ACM Transactions on Graphics 4: 74-123.

[23] Mahoney PA, Weber U, Onofrechuk P, Biessmann H, Bryant PJ, et al. (1991) The *fat* tumor suppressor gene in *Drosophila* encodes a novel member of the cadherin gene superfamily. Cell 67: 853–868.

[24] Bryant PJ, Huettner B, Held LI, Ryerse J, Szidonya J (1988) Mutations at the *fat* locus interfere with cell proliferation control and epithelial morphogenesis in *Drosophila*. Developmental Biology 129: 541-554.

[25] Garoia F, Guerra D, Pezzoli MC, López-Varea A, Cavicchi S, et al. (2000) Cell behaviour of *Drosophila fat* cadherin mutations in wing development. Mechanisms of Development 94: 95–109.

[26] Clark HF, Brentrup D, Schneitz K, Bieber A, Goodman C, et al. (1995) *Dachsous* encodes a member of the cadherin superfamily that controls imaginal disc morphogenesis in *Drosophila*. Genes & Development 9: 1530–1542.

[27] Rodríguez I (2004) The *dachsous* gene, a member of the cadherin family, is required for Wg-dependent pattern formation in the *Drosophila* wing disc. Development 131: 3195–3206.

[28] Strutt H, Mundy J, Hofstra K, Strutt D (2004) Cleavage and secretion is not required for Four-jointed function in *Drosophila* patterning. Development 131: 881–890.

[29] Brodsky MH, Steller H (1996) Positional information along the dorsal-ventral axis of the *Drosophila* eye: graded expression of the *four-jointed* gene. Developmental Biology 173: 428–446.

[30] Villano JL, Katz FN (1995) Four-jointed is required for intermediate growth in the proximal-distal axis in *Drosophila*. Development 121: 2767–2777.

[31] Zeidler MP, Perrimon N, Strutt DI (2000) Multiple roles for *four-jointed* in planar polarity and limb patterning. Developmental Biology 228: 181–196.

[32] Campbell G, Tomlinson A (1999) Transducing the Dpp morphogen gradient in the wing of *Drosophila*: Regulation of dpp targets by *brinker*. Cell 96: 553–562.

[33] Schwank G, Restrepo S, Basler K (2008) Growth regulation by Dpp: an essential role for Brinker and a non-essential role for graded signaling levels. Development 135: 4003–4013.

[34] Posakony LG, Raftery LA, Gelbart WM (1991) Wing formation in *Drosophila melanogaster* requires *decapentaplegic* gene function along the anterior-posterior compartment boundary. Mechanisms of Development 33: 69–82.

[35] Willecke M, Hamaratoglu F, Sansores-Garcia L, Tao C, Halder G (2008) Boundaries of Dachsous cadherin activity modulate the Hippo signaling pathway to induce cell proliferation. Proceedings of the National Academy of Sciences 105: 14897–14902.

[36] Mani M, Goyal S, Irvine KD, Shraiman BI (2013) Collective polarization model for gradient sensing via Dachsous-Fat intercellular signaling. Proceedings of the National Academy of Sciences 110: 20420–20425.

[37] Wu J, Cohen SM (2002) Repression of Teashirt marks the initiation of wing development. Development 129: 2411–2418.
