# Supplementary material for: Expanding signaling-molecule wavefront model of cell polarization in the Drosophila wing primordium
Source: PLoS Comput Biol. 2017 Jul 3;13(7):e1005610. doi: 10.1371/journal.pcbi.1005610 (PMC5515495; doi:10.1371/journal.pcbi.1005610)
Supplement: S1 Table — (PDF) [file pcbi.1005610.s033.pdf]

**Name**	**Equation**	**Description**	**Value**
		Morphogen profile	
C_0	(S9)	Beginning amplitude of the morphogen profile	1.5
t_0	(S9)	Time over which the amplitude increases	1200 min
$[M]_c$	(S10), (S11)	Morphogen concentration at which the Ds profile has an inflection point	0.5
A	(S9)	Ratio of disc radius to morphogen decay length	4
m	(S10)	Steepness of dependence of Ds expression on morphogen concentration	10
		Specific conditions	
–	–	Overall multiplier for Ds expression when Ds is ubiquitous	10
–	–	Fj expression level when Fj is ubiquitous	10
–	–	Starting front location (radial distance) when the front is stationary	30 μm

Table S1: Parameters used in the morphogen profile and for specific disc genotypes.
