# Supplementary material for: Expanding signaling-molecule wavefront model of cell polarization in the Drosophila wing primordium
Source: PLoS Comput Biol. 2017 Jul 3;13(7):e1005610. doi: 10.1371/journal.pcbi.1005610 (PMC5515495; doi:10.1371/journal.pcbi.1005610)
Supplement: S2 Table — (PDF) [file pcbi.1005610.s034.pdf]

Name	Equation	Description	Value
		Growth rate	
G_0	(S20)	Overall multiplier to the growth rate	4.3×10^{-4} min^{-1}
C_{Ft}	(S20)	Contribution of Ft asymmetry to the growth rate	3×10^3 min
C_{Ds}	(S20)	Contribution of Ds asymmetry to the growth rate	3×10^3 min
C_M	(S20)	Contribution of morphogen signaling to the growth rate	40 min
U_i	(S20)	Penalty for leftover free Ft and Ds on cell i	$3 \times 10^{-5} \times$ (free Ft_i + free Ds_i)
–	–	Grace period before counting asymmetry from a new neighbor	100 min
		Cell division	
A_c	(S8)	Critical area for cell division	4π μm^2
–	–	Maximum amount by which the radii of daughter cells differ after division	1%
ℓ	(S8)	Steepness of division probability vs. radius	400
		Integral feedback	
G_{Ft}	(S23)	Time scale for Ft asymmetry feedback	6×10^{-4} min^{-1}
G_{Ds}	(S25)	Time scale for Ds asymmetry feedback	6×10^{-4} min^{-1}
G_M	(S21)	Time scale for morphogen signaling feedback	2.8×10^{-2} min^{-1}
		Neighbor list	
–	–	Fraction of a cell's radius a cell must move before a full neighbor rebuild is triggered	0.2 radii
–	–	Time before a full neighbor rebuild is triggered, independent of cell motion	10 min
		Bulk interactions	
–	–	Threshold membrane-membrane distance for interactions	0.8 μm
γ	(S5)	Drag coefficient	2×10^{-3} min^{-1}
C_{gap}	(S3)	Strength of attractive force	3×10^{-7} μm^2 min^{-2}
$C_{overlap}$	(S4)	Strength of repulsive force	1×10^{-6} min^{-2}

Table S2: Parameters used for cell growth, division, and interaction.
