# Supplementary material for: Expanding signaling-molecule wavefront model of cell polarization in the Drosophila wing primordium
Source: PLoS Comput Biol. 2017 Jul 3;13(7):e1005610. doi: 10.1371/journal.pcbi.1005610 (PMC5515495; doi:10.1371/journal.pcbi.1005610)
Supplement: S3 Table — (PDF) [file pcbi.1005610.s035.pdf]

| **Name** | **Appears in** | **Description** | **Value** |
|---|---|---|---|
| | | Ft, Ds, and Fj expression | |
| $\text{Ft}_{max}$ | – | Starting Ft, overall Ft expression level | $1 \times 10^3$ proteins |
| $A_{Ds,initial}$ | (S10) | Scaling coefficient for initial Ds | $1 \times 10^3$ proteins |
| $R_{Ft}$ | (S15) | Ft expression rate | 20 proteins/min |
| $R_{Ds,max}$ | (S16) | Base Ds expression rate | 20 proteins/min |
| $R_{Fj,max}$ | (S17) | Base Fj expression rate | 0.05 $\text{min}^{-1}$ |
| $\text{Ds}_{min}$ | (S16) | Minimum level of Ds expression in terms of $R_{Ds,max}$ | 0.2 |
| $\text{Ds}_{range}$ | (S16) | Range of Ds expression in terms of $R_{Ds,max}$ | 0.8 |
| $[\text{Fj}]_{min}$ | (S11) | Minimum Fj concentration | 0.1 |
| $\text{Fj}_{slope}$ | (S11) | Slope of Fj expression profile | 0.025 $\mu\text{m}^{-1}$ |
| | | Ft-Ds binding | |
| $C_1$ | (S12), (S18) | Maximum chance of Ft being bindable (before offset) | 0.8 |
| $C_2$ | (S13), (S19) | Maximum chance of Ds being bindable (before offset) | 0.8 |
| $[\text{Fj}]_c$ | (S14) | Critical value of Fj (determines phosphorylation probabilities for Ft and Ds) | 0.3 |
| $n$ | (S14) | Steepness of dependence of phosphorylation rates on Fj | 2 |
| $P_{\text{offset}}$ | (S12) | Minimum probability of Ft/Ds being phosphorylated | 0.1 |
| $k_{dissoc}$ | – | Fraction of bonds unbound per step | 0.08 $\text{min}^{-1}$ |
| $k_{d,Ft}$ | (S15) | Fraction of free Ft degraded per unit time | 0.08 $\text{min}^{-1}$ |
| $k_{d,Ds}$ | (S16) | Fraction of free Ds degraded per unit time | 0.08 $\text{min}^{-1}$ |
| $k_{d,Fj}$ | (S17) | Fraction of Fj degraded per unit time | 0.05 $\text{min}^{-1}$ |
| $D$ | (S18), (S19) | Dephosphorylation rate of free Ft and Ds | $1.28 \times 10^{-3}$ $\text{min}^{-1}$ |
| $P_{Ft,Ds}$ | - | Weight for Ft-Ds bond formation | 0.05 |
| $P_{FtP,Ds}$ | - | Weight for FtP-Ds bond formation | 0.7 |
| $P_{Ft,DsP}$ | - | Weight for Ft-DsP bond formation | 0.05 |
| $P_{FtP,DsP}$ | - | Weight for FtP-DsP bond formation | 0.2 |

Table S3: Parameters used in the expression and binding of Ft, Ds, and Fj.
